# Ichthyofauna of the Kubo, Tochikura, and Ichinono river systems (Kitakami River drainage, northern Japan), with a comparison of predicted and surveyed species richness

**DOI:** 10.3897/BDJ.2.e1093

**Published:** 2014-11-07

**Authors:** Yusuke Miyazaki, Masanori Nakae, Hiroshi Senou

**Affiliations:** † Kanagawa Prefectural Museum of Natural History, Kanagawa, Japan; ‡ The University of Tokyo, Tokyo, Japan; § National Museum of Nature and Science, Ibaraki, Japan

**Keywords:** Act on the Promotion of Nature Restoration, identification, nature restoration committee, potential species pool

## Abstract

The potential fish species pool of the Kubo, Tochikura, and Ichinono river systems (tributaries of the Iwai River, Kitakami River drainage), Iwate Prefecture, northern Japan, was compared with the observed ichthyofauna by using historical records and new field surveys. Based on the literature survey, the potential species pool comprised 24 species/subspecies but only 20, including 7 non-native taxa, were recorded during the fieldwork. The absence during the survey of 11 species/subspecies from the potential species pool suggested either that sampling effort was insufficient, or that accurate determination of the potential species pool was hindered by lack of biogeographic data and ecological data related to the habitat use of the species. With respect to freshwater fish conservation in the area, *Lethenteron reissneri*, *Carassius auratus buergeri*, *Pseudorasbora pumila*, *Tachysurus tokiensis*, *Oryzias latipes*, and *Cottus nozawae* are regarded as priority species, and *Cyprinus rubrofuscus*, *Pseudorasbora parva*, and *Micropterus salmoides* as targets for removal.

## Introduction

Biodiversity is rapidly declining at a global level due to a variety of anthropogenic pressures (Myers et al. 2000, Roberts et al. 2002, Brooks et al. 2006). Japan’s biodiversity is no exception, primarily because of the country’s high human population density and its status as a biodiversity hotspot with high endemism, including among freshwater fishes (Boufford et al. 2004, Brooks et al. 2006). Against this background, activities for the conservation and/or preservation of freshwater fishes have expanded in many regions of Japan. These include a number of nature restoration projects based on the Law for the Promotion of Nature Restoration, a Japanese domestic law that took effect in 2003 (Fujimoto et al. 2008, Matsuzaki et al. 2011).

Understanding a region’s biota is critical for successful nature restoration projects, particularly with respect to biodiversity conservation. When detailed survey data are lacking, determining a region’s potential species pool (Lessard et al. 2012) can provide an estimate of local biodiversity status by application of the ecological filter concept (Fattorini and Halle 2004, Hobbs and Norton 2004, Funk et al. 2008). Based on this concept, species diversity at the local scale is presumed to be constrained by the regional species pool, and therefore filtered by stochastic and deterministic processes including both non-anthropogenic and/or anthropogenic factors. Such factors exert pressures on regional species diversity over longer time frames than more recent influences such as the present biodiversity crisis. An understanding of the biogeography of a region can allow us to compile a list of potential species that provides baseline data for the evaluation and design of an action plan for regional biodiversity conservation and restoration.

The nature restoration committee of the Kubo-gawa Ihatov area represents one of the projects based on the Law for the Promotion of Nature Restoration, but is unique in being overseen by private sector administration (Chisaka 2010). Part of this project has focused on the conservation and restoration of the freshwater fishes of the region, which includes most of the Kubo, Tochikura and Ichinono river systems.

In this study, we consulted historical literature to determine the potential species pool of this region (i.e., the species we predict to occur there), and carried out comprehensive field surveys to "ground truth" this list, as well as to identify fish species of conservation and restoration concern.

## Materials and methods

**Figure 1.**
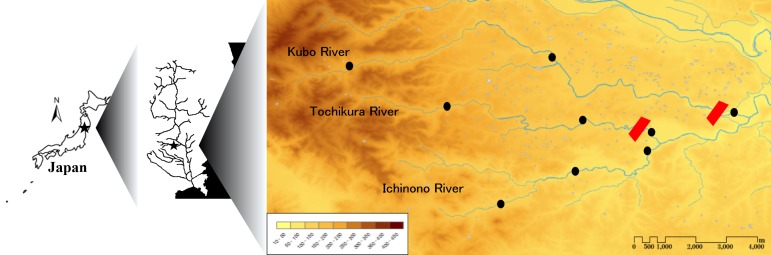
Map of the Kubo, Tochikura, and Ichinono river systems. Black circles and red trapezoids indicate the main survey points where fish sampling was conducted and sediment control dams, respectively.

The study region is located in the Kubo, Tochikura and Ichinono river systems (38°52′–38°55′ N, 140°56′–141°03′ E; Fig. 1), which are all tributaries of the Iwai River within the Kitakami River drainage that empties into the Pacific Ocean (Ichinoseki City, Iwate Prefecture, northern Japan). It includes the headwaters of these rivers, and headwater areas in smaller valleys (Fig. 1). The Kubo and Tochikura rivers each have a sediment control dam constructed in the lower reaches (Fig. 1). Rural cultivation and the typical ecological networks created by rice paddy ecosystems (traditional secondary nature, “*satoyama*” landscape: Washitani 2008) are widely established in the region (Ohtani et al. 2013; Fig. 2a).

The potential fish species pool of the study region was determined by a review of existing literature (Kawanabe et al. 2001, Ozawa et al. 2003, Matsuzawa and Senou 2008, Kumagai and Katsushima 2012, Nakabo 2013) as follows: the freshwater fishes with a natural distributional range located entirely or partly within Iwate Prefecture were catalogued, and the subsequent list was refined by considering ecology of the individual species and geography of the study region (i.e., whether they are known to inhabit headwaters and/or upper reaches of Japanese rivers or not). In other words, the fish species included in the potential species pool were determined by literature reports recording the species from the headwaters and/or upper reaches of rivers in Japan. For example, Kawanabe et al. (2001) reported that the Japanese eel, *Anguilla japonica*, often inhabits the upper reaches of Japanese rivers including Iwate Prefecture, and therefore we included *A. japonica* in our potential species pool of the region.

The fish fauna was assessed during field surveys using hand nets, minnow traps, cast nets, set nets, and hook-and-line angling. More than 200 irrigation ponds and three sampling sites (upper, middle and lower reaches) in each of the three rivers were sampled from April to October in 2008, 2009 and 2010 (Fig. 1). The substratum of the irrigation ponds consisted mainly of silt and organic litter with aquatic plants (Fig. 2b, c). The substratum of the river sites consisted mainly of cobble and gravel, often with boulders, as evident in the structure of one pool and riffle unit (Fig. 2d, e, f).

Voucher specimens and photographs are deposited in the Kanagawa Prefectural Museum of Natural History, Odawara, Japan (KPM) and the National Museum of Nature and Science, Tsukuba, Japan (NSMT). Unless otherwise stated, the systematic arrangement of families and scientific names follows Eschmeyer (2014), while that of standard Japanese names follows Nakabo (2013).

To evaluate the accuracy of the potential species pool (the predicted ichthyofauna), we made rarefaction curves based on presence/absence data from our field surveys. This allows us to estimate the number of additional species that our surveys may have missed. These analyses were conducted using EstimateS with the bias-corrected formula (Chao et al. 2004, Colwell et al. 2004, Colwell et al. 2012).

## Data resources

The voucher specimens and photographs are deposited and registered as KPM-NI 19440, 19447–19454, 211181–21203, 21217–21227, 21398–21413, 22248–22313, 22429–22432, 23639–23659, 23738–23745, 23933–24000, 24396–24398, 24407–24419, 24466–24476, 24988–24994, 30997–30999, 31003, 31005–31007, 31009, 31011, 31018, 31715–31811, and 35077–35105, KPM-NR 43909–43911, and NSMT-P 90703–90723, 91185–91208, 91638–91652, 92194–92195, 96055–96061, 96828–96833, 96836–96860, 96890–96902, 97045, 97084, and 97109–97111.

The information of these vouchers has been submitted to the Global Information Facility (http://www.gbif.org/) via the museums.

## Checklists

### Checklist of the fishes of the Kubo, Tochikura, and Ichinono River Basins

#### Lethenteron reissneri

(Dybowski, 1869)

##### Materials

- **Type status:** Other material. **Occurrence:** catalogNumber: KPM-NI 21186; recordedBy: Shin-ichi Suda; individualCount: 1; **Taxon:** scientificName: Lethenteron reissneri; **Location:** country: Japan; stateProvince: Iwate; locality: Ichinono River; verbatimLatitude: 38°54′19″N; verbatimLongitude: 141°02′41″E; **Identification:** identifiedBy: Yusuke Miyazaki; dateIdentified: 2008; **Event:** year: 2008; month: 5; day: 3; **Record Level:** basisOfRecord: PreservedSpecimen
- **Type status:** Other material. **Occurrence:** catalogNumber: KPM-NI 21195; recordedBy: Hiroshi Senou; individualCount: 1; **Taxon:** scientificName: Lethenteron reissneri; **Location:** country: Japan; stateProvince: Iwate; locality: Tochikura River; verbatimLatitude: 38°54′43.05″N; verbatimLongitude: 141°01′24.39″E; **Identification:** identifiedBy: Hiroshi Senou; dateIdentified: 2008; **Event:** year: 2008; month: 5; day: 2; **Record Level:** basisOfRecord: PreservedSpecimen
- **Type status:** Other material. **Occurrence:** catalogNumber: KPM-NI 23996; recordedBy: Yusuke Miyazaki; individualCount: 1; **Taxon:** scientificName: Lethenteron reissneri; **Location:** country: Japan; stateProvince: Iwate; locality: Kubo River; verbatimLatitude: 38°55′38″N; verbatimLongitude: 140°56′57″E; **Identification:** identifiedBy: Yusuke Miyazaki; dateIdentified: 2009; **Event:** year: 2009; month: 4; day: 30; **Record Level:** basisOfRecord: PreservedSpecimen
- **Type status:** Other material. **Occurrence:** catalogNumber: KPM-NI 23997; recordedBy: Yusuke Miyazaki; individualCount: 1; **Taxon:** scientificName: Lethenteron reissneri; **Location:** country: Japan; stateProvince: Iwate; locality: Ichinono River; verbatimLatitude: 38°54′16″N; verbatimLongitude: 141°02′43″E; **Identification:** identifiedBy: Yusuke Miyazaki; dateIdentified: 2009; **Event:** year: 2009; month: 5; day: 1; **Record Level:** basisOfRecord: PreservedSpecimen
- **Type status:** Other material. **Occurrence:** catalogNumber: KPM-NI 23998; recordedBy: Yusuke Miyazaki; individualCount: 1; **Taxon:** scientificName: Lethenteron reissneri; **Location:** country: Japan; stateProvince: Iwate; locality: Kubo River; verbatimLatitude: 38°54′55″N; verbatimLongitude: 141°04′24″E; **Identification:** identifiedBy: Yusuke Miyazaki; dateIdentified: 2009; **Event:** year: 2009; month: 5; day: 2; **Record Level:** basisOfRecord: PreservedSpecimen
- **Type status:** Other material. **Occurrence:** catalogNumber: NSMT-P 96842; recordedBy: Yusuke Miyazaki and Yoko Takata; individualCount: 1; **Taxon:** scientificName: Lethenteron reissneri; **Location:** country: Japan; stateProvince: Iwate; locality: Ichinono River; **Identification:** identifiedBy: Yoko Takata; dateIdentified: 2010; **Event:** year: 2008; month: 10; day: 9; **Record Level:** basisOfRecord: PreservedSpecimen
- **Type status:** Other material. **Occurrence:** catalogNumber: NSMT-P 96900; recordedBy: Yoko Takata; individualCount: 1; **Taxon:** scientificName: Lethenteron reissneri; **Location:** country: Japan; stateProvince: Iwate; locality: Ichinono River; verbatimLatitude: 38°54′03″N; verbatimLongitude: 141°02′07″E; **Identification:** identifiedBy: Yoko Takata; dateIdentified: 2010; **Event:** year: 2008; month: 11; day: 8; **Record Level:** basisOfRecord: PreservedSpecimen

##### Ecological interactions

###### Conservation status

National: VU (Ministry of the Environment, Japan 2013); Prefectural: NT (Iwate Prefecture 2014).

##### Distribution

Arctic and Pacific Ocean drainage.

##### Notes

This taxon is morphologically identical to *L.* sp. N and *L.* sp. S of Yamazaki et al. (2006). These two putative species of *Lethenteron* are recognized only by a molecular analysis (Yamazaki et al. 2006), so we treated them as one species complex here because of the difficulty in differentiating the species on morphological characters alone (see Nakabo and Kai 2013 and its citations). Adults were observed in lotic environments, while larvae were observed in lentic environments such as in the silty/sandy bottom of floodplains.

#### Cyprinus rubrofuscus

Lacepède, 1803

##### Materials

- **Type status:** Other material. **Occurrence:** catalogNumber: KPM-NI 22277; recordedBy: Yusuke Miyazaki; individualCount: 1; **Taxon:** scientificName: Cyprinus rubrofuscus; **Location:** country: Japan; stateProvince: Iwate; locality: irrigation pond of the Kubo River Basin; verbatimLatitude: 38°55′17.3″N; verbatimLongitude: 140°59′26.7″E; **Identification:** identifiedBy: Yusuke Miyazaki; dateIdentified: 2008; **Event:** year: 2008; month: 8; day: 27; **Record Level:** basisOfRecord: PreservedSpecimen
- **Type status:** Other material. **Occurrence:** catalogNumber: KPM-NI 22278; recordedBy: Yusuke Miyazaki; individualCount: 1; **Taxon:** scientificName: Cyprinus rubrofuscus; **Location:** country: Japan; stateProvince: Iwate; locality: irrigation pond of the Kubo River Basin; verbatimLatitude: 38°56′20.3″N; verbatimLongitude: 140°59′18.8″E; **Identification:** identifiedBy: Yusuke Miyazaki; dateIdentified: 2008; **Event:** year: 2008; month: 8; day: 29; **Record Level:** basisOfRecord: PreservedSpecimen
- **Type status:** Other material. **Occurrence:** catalogNumber: NSMT-P 96829; recordedBy: Yusuke Miyazaki and Yoko Takata; individualCount: 1; **Taxon:** scientificName: Cyprinus rubrofuscus; **Location:** country: Japan; stateProvince: Iwate; locality: Tochikura River; **Identification:** identifiedBy: Mikumi Takada; dateIdentified: 2010; **Event:** year: 2008; month: 10; day: 9; **Record Level:** basisOfRecord: PreservedSpecimen
- **Type status:** Other material. **Occurrence:** catalogNumber: NSMT-P 96836; recordedBy: Yusuke Miyazaki; individualCount: 5; **Taxon:** scientificName: Cyprinus rubrofuscus; **Location:** country: Japan; stateProvince: Iwate; locality: irrigation pond of the Kubo River Basin; **Identification:** identifiedBy: Yoko Takata; dateIdentified: 2010; **Event:** year: 2008; month: 10; day: 10; **Record Level:** basisOfRecord: PreservedSpecimen
- **Type status:** Other material. **Occurrence:** catalogNumber: NSMT-P 96850; recordedBy: Kazuo Satou; individualCount: 1; **Taxon:** scientificName: Cyprinus rubrofuscus; **Location:** country: Japan; stateProvince: Iwate; locality: irrigation pond of the Ichinono River Basin; **Identification:** identifiedBy: Kaoru Kuriiwa; dateIdentified: 2010; **Event:** year: 2008; month: 10; day: 9; **Record Level:** basisOfRecord: PreservedSpecimen

##### Ecological interactions

###### Native status

Non-native (100 of the World's Worst Invasive Alien Species: Lowe et al. 2000, Matsuzawa and Senou 2008). Native Japanese populations of the species have been widely affected by introgression due to human introductions, except for the population of north Lake Biwa (Mabuchi et al. 2005, Mabuchi et al. 2006, Mabuchi et al. 2008, Mabuchi et al. 2010), so therefore we regarded the carp populations of the region as non-native species.

##### Distribution

Laos, Vietnam, and China (Amur to Red River drainages).

##### Notes

This taxon is identical with *Cyprinus carpio* of Hosoya (2013). This species was observed almost always from lentic environments, but occasionally from lotic environments.

#### Carassius cuvieri

Temminck & Schlegel, 1846

##### Materials

- **Type status:** Other material. **Occurrence:** catalogNumber: KPM-NI 30997; recordedBy: Yusuke Miyazaki; individualCount: 1; **Taxon:** scientificName: Carassius cuvieri; **Location:** country: Japan; stateProvince: Iwate; locality: irrigation pond of the Kubo River Basin; verbatimLatitude: 38°55′49″N; verbatimLongitude: 141°01′33″E; **Identification:** identifiedBy: Yusuke Miyazaki; dateIdentified: 2010; **Event:** year: 2010; month: 9; day: 5; **Record Level:** basisOfRecord: PreservedSpecimen

##### Ecological interactions

###### Native status

Non-native (domestic non-native Species in Iwate Prefecture: Matsuzawa and Senou 2008). Native Japanese populations of the species have been widely affected by introgression due to human introductions except for the population of north Lake Biwa (Mabuchi et al. 2005, Mabuchi et al. 2006, Mabuchi et al. 2008, Mabuchi et al. 2010), so therefore we regarded the populations of the region as non-native species.

##### Distribution

Endemic to Lake Biwa, Japan.

##### Notes

This species was captured an irrigation pond with dense aquatic plants.

#### Carassius auratus buergeri

(Temminck & Schlegel, 1846)

##### Materials

- **Type status:** Other material. **Occurrence:** catalogNumber: KPM-NI 21196; recordedBy: Yusuke Miyazaki; individualCount: 1; **Taxon:** scientificName: Carassius auratus buergeri; **Location:** country: Japan; stateProvince: Iwate; locality: irrigation pond of the Ichinono River Basin; verbatimLatitude: 38°53′43″N; verbatimLongitude: 140°58′49″E; **Identification:** identifiedBy: Yusuke Miyazaki; dateIdentified: 2013; **Event:** year: 2008; month: 5; day: 5; **Record Level:** basisOfRecord: PreservedSpecimen
- **Type status:** Other material. **Occurrence:** catalogNumber: KPM-NI 21199; recordedBy: Yusuke Miyazaki; individualCount: 1; **Taxon:** scientificName: Carassius auratus buergeri; **Location:** country: Japan; stateProvince: Iwate; locality: irrigation pond of the Ichinono River Basin; verbatimLatitude: 38°53′51″N; verbatimLongitude: 140°59′55″E; **Identification:** identifiedBy: Yusuke Miyazaki; dateIdentified: 2013; **Event:** year: 2008; month: 5; day: 6; **Record Level:** basisOfRecord: PreservedSpecimen
- **Type status:** Other material. **Occurrence:** catalogNumber: KPM-NI 21200; recordedBy: Yusuke Miyazaki; individualCount: 1; **Taxon:** scientificName: Carassius auratus buergeri; **Location:** country: Japan; stateProvince: Iwate; locality: irrigation pond of the Ichinono River Basin; verbatimLatitude: 38°53′51″N; verbatimLongitude: 140°59′55″E; **Identification:** identifiedBy: Yusuke Miyazaki; dateIdentified: 2013; **Event:** year: 2008; month: 5; day: 6; **Record Level:** basisOfRecord: PreservedSpecimen
- **Type status:** Other material. **Occurrence:** catalogNumber: KPM-NI 21402; recordedBy: Yusuke Miyazaki; individualCount: 1; **Taxon:** scientificName: Carassius auratus buergeri; **Location:** country: Japan; stateProvince: Iwate; locality: irrigation pond of the Tochikura River Basin; verbatimLatitude: 38°54′35″N; verbatimLongitude: 141°01′07″E; **Identification:** identifiedBy: Yusuke Miyazaki; dateIdentified: 2013; **Event:** year: 2008; month: 6; day: 8; **Record Level:** basisOfRecord: PreservedSpecimen
- **Type status:** Other material. **Occurrence:** catalogNumber: KPM-NI 21404; recordedBy: Yusuke Miyazaki; individualCount: 1; **Taxon:** scientificName: Carassius auratus buergeri; **Location:** country: Japan; stateProvince: Iwate; locality: irrigation pond of the Kubo River Basin; verbatimLatitude: 38°55′28″N; verbatimLongitude: 141°00′03″E; **Identification:** identifiedBy: Yusuke Miyazaki; dateIdentified: 2013; **Event:** year: 2008; month: 6; day: 4; **Record Level:** basisOfRecord: PreservedSpecimen
- **Type status:** Other material. **Occurrence:** catalogNumber: KPM-NI 21406; recordedBy: Yusuke Miyazaki; individualCount: 1; **Taxon:** scientificName: Carassius auratus buergeri; **Location:** country: Japan; stateProvince: Iwate; locality: irrigation pond of the Ichinono River Basin; verbatimLatitude: 38°53′48″N; verbatimLongitude: 141°01′31″E; **Identification:** identifiedBy: Yusuke Miyazaki; dateIdentified: 2013; **Event:** year: 2008; month: 6; day: 4; **Record Level:** basisOfRecord: PreservedSpecimen
- **Type status:** Other material. **Occurrence:** catalogNumber: KPM-NI 21407; recordedBy: Yusuke Miyazaki; individualCount: 1; **Taxon:** scientificName: Carassius auratus buergeri; **Location:** country: Japan; stateProvince: Iwate; locality: irrigation pond of the Ichinono River Basin; verbatimLatitude: 38°54′10″N; verbatimLongitude: 140°59′51″E; **Identification:** identifiedBy: Yusuke Miyazaki; dateIdentified: 2013; **Event:** year: 2008; month: 6; day: 5; **Record Level:** basisOfRecord: PreservedSpecimen
- **Type status:** Other material. **Occurrence:** catalogNumber: KPM-NI 22248; recordedBy: Yusuke Miyazaki; individualCount: 1; **Taxon:** scientificName: Carassius auratus buergeri; **Location:** country: Japan; stateProvince: Iwate; locality: irrigation pond of the Ichinono River Basin; verbatimLatitude: 38°53′25.7″N; verbatimLongitude: 141°00′18.0″E; **Identification:** identifiedBy: Yusuke Miyazaki; dateIdentified: 2013; **Event:** year: 2008; month: 7; day: 10; **Record Level:** basisOfRecord: PreservedSpecimen
- **Type status:** Other material. **Occurrence:** catalogNumber: KPM-NI 22249; recordedBy: Yusuke Miyazaki; individualCount: 1; **Taxon:** scientificName: Carassius auratus buergeri; **Location:** country: Japan; stateProvince: Iwate; locality: irrigation pond of the Ichinono River Basin; verbatimLatitude: 38°53′25.7″N; verbatimLongitude: 141°00′18.0″E; **Identification:** identifiedBy: Yusuke Miyazaki; dateIdentified: 2013; **Event:** year: 2008; month: 7; day: 10; **Record Level:** basisOfRecord: PreservedSpecimen
- **Type status:** Other material. **Occurrence:** catalogNumber: KPM-NI 22300; recordedBy: Yusuke Miyazaki; individualCount: 1; **Taxon:** scientificName: Carassius auratus buergeri; **Location:** country: Japan; stateProvince: Iwate; locality: irrigation pond of the Ichinono River Basin; verbatimLatitude: 38°53′48.4″N; verbatimLongitude: 141°01′30.2″E; **Identification:** identifiedBy: Yusuke Miyazaki; dateIdentified: 2013; **Event:** year: 2008; month: 9; day: 14; **Record Level:** basisOfRecord: PreservedSpecimen
- **Type status:** Other material. **Occurrence:** catalogNumber: KPM-NI 22301; recordedBy: Yusuke Miyazaki; individualCount: 1; **Taxon:** scientificName: Carassius auratus buergeri; **Location:** country: Japan; stateProvince: Iwate; locality: irrigation pond of the Ichinono River Basin; verbatimLatitude: 38°53′48.4″N; verbatimLongitude: 141°01′30.2″E; **Identification:** identifiedBy: Yusuke Miyazaki; dateIdentified: 2013; **Event:** year: 2008; month: 9; day: 14; **Record Level:** basisOfRecord: PreservedSpecimen
- **Type status:** Other material. **Occurrence:** catalogNumber: KPM-NI 22305; recordedBy: Yusuke Miyazaki; individualCount: 1; **Taxon:** scientificName: Carassius auratus buergeri; **Location:** country: Japan; stateProvince: Iwate; locality: irrigation pond of the Tochikura River Basin; verbatimLatitude: 38°54′36″N; verbatimLongitude: 141°01′07″E; **Identification:** identifiedBy: Yusuke Miyazaki; dateIdentified: 2013; **Event:** year: 2008; month: 9; day: 15; **Record Level:** basisOfRecord: PreservedSpecimen
- **Type status:** Other material. **Occurrence:** catalogNumber: KPM-NI 22306; recordedBy: Yusuke Miyazaki; individualCount: 1; **Taxon:** scientificName: Carassius auratus buergeri; **Location:** country: Japan; stateProvince: Iwate; locality: irrigation pond of the Ichinono River Basin; verbatimLatitude: 38°53′48.9″N; verbatimLongitude: 141°01′43.5″E; **Identification:** identifiedBy: Yusuke Miyazaki; dateIdentified: 2013; **Event:** year: 2008; month: 9; day: 14; **Record Level:** basisOfRecord: PreservedSpecimen
- **Type status:** Other material. **Occurrence:** catalogNumber: KPM-NI 22307; recordedBy: Yusuke Miyazaki; individualCount: 1; **Taxon:** scientificName: Carassius auratus buergeri; **Location:** country: Japan; stateProvince: Iwate; locality: irrigation pond of the Ichinono River Basin; verbatimLatitude: 38°53′48.9″N; verbatimLongitude: 141°01′43.5″E; **Identification:** identifiedBy: Yusuke Miyazaki; dateIdentified: 2013; **Event:** year: 2008; month: 9; day: 14; **Record Level:** basisOfRecord: PreservedSpecimen
- **Type status:** Other material. **Occurrence:** catalogNumber: KPM-NI 22308; recordedBy: Yusuke Miyazaki; individualCount: 1; **Taxon:** scientificName: Carassius auratus buergeri; **Location:** country: Japan; stateProvince: Iwate; locality: irrigation pond of the Ichinono River Basin; verbatimLatitude: 38°53′48.9″N; verbatimLongitude: 141°01′43.5″E; **Identification:** identifiedBy: Yusuke Miyazaki; dateIdentified: 2013; **Event:** year: 2008; month: 9; day: 14; **Record Level:** basisOfRecord: PreservedSpecimen
- **Type status:** Other material. **Occurrence:** catalogNumber: KPM-NI 22309; recordedBy: Yusuke Miyazaki; individualCount: 1; **Taxon:** scientificName: Carassius auratus buergeri; **Location:** country: Japan; stateProvince: Iwate; locality: irrigation pond of the Ichinono River Basin; verbatimLatitude: 38°53′51.5″N; verbatimLongitude: 140°59′55.4″E; **Identification:** identifiedBy: Yusuke Miyazaki; dateIdentified: 2013; **Event:** year: 2008; month: 9; day: 17; **Record Level:** basisOfRecord: PreservedSpecimen
- **Type status:** Other material. **Occurrence:** catalogNumber: KPM-NI 22310; recordedBy: Yusuke Miyazaki; individualCount: 1; **Taxon:** scientificName: Carassius auratus buergeri; **Location:** country: Japan; stateProvince: Iwate; locality: irrigation pond of the Ichinono River Basin; verbatimLatitude: 38°53′51.5″N; verbatimLongitude: 140°59′55.4″E; **Identification:** identifiedBy: Yusuke Miyazaki; dateIdentified: 2013; **Event:** year: 2008; month: 9; day: 17; **Record Level:** basisOfRecord: PreservedSpecimen
- **Type status:** Other material. **Occurrence:** catalogNumber: KPM-NI 22311; recordedBy: Yusuke Miyazaki; individualCount: 1; **Taxon:** scientificName: Carassius auratus buergeri; **Location:** country: Japan; stateProvince: Iwate; locality: irrigation pond of the Ichinono River Basin; verbatimLatitude: 38°53′51.5″N; verbatimLongitude: 140°59′55.4″E; **Identification:** identifiedBy: Yusuke Miyazaki; dateIdentified: 2013; **Event:** year: 2008; month: 9; day: 17; **Record Level:** basisOfRecord: PreservedSpecimen
- **Type status:** Other material. **Occurrence:** catalogNumber: KPM-NI 22312; recordedBy: Yusuke Miyazaki; individualCount: 1; **Taxon:** scientificName: Carassius auratus buergeri; **Location:** country: Japan; stateProvince: Iwate; locality: irrigation pond of the Kubo River Basin; verbatimLatitude: 38°56′15.9″N; verbatimLongitude: 141°01′01.0″E; **Identification:** identifiedBy: Yusuke Miyazaki; dateIdentified: 2013; **Event:** year: 2008; month: 8; day: 25; **Record Level:** basisOfRecord: PreservedSpecimen
- **Type status:** Other material. **Occurrence:** catalogNumber: KPM-NI 23647; recordedBy: Yusuke Miyazaki; individualCount: 1; **Taxon:** scientificName: Carassius auratus buergeri; **Location:** country: Japan; stateProvince: Iwate; locality: irrigation pond of the Kubo River Basin; verbatimLatitude: 38°55′19.4″N; verbatimLongitude: 140°59′29.1″E; **Identification:** identifiedBy: Yusuke Miyazaki; dateIdentified: 2013; **Event:** year: 2008; month: 6; day: 4; **Record Level:** basisOfRecord: PreservedSpecimen
- **Type status:** Other material. **Occurrence:** catalogNumber: KPM-NI 23744; recordedBy: Yusuke Miyazaki; individualCount: 1; **Taxon:** scientificName: Carassius auratus buergeri; **Location:** country: Japan; stateProvince: Iwate; locality: Kubo River; locationRemarks: the middle reach; **Identification:** identifiedBy: Yusuke Miyazaki; dateIdentified: 2013; **Event:** year: 2009; month: 5; day: 3; **Record Level:** basisOfRecord: PreservedSpecimen
- **Type status:** Other material. **Occurrence:** catalogNumber: KPM-NI 23745; recordedBy: Yusuke Miyazaki; individualCount: 1; **Taxon:** scientificName: Carassius auratus buergeri; **Location:** country: Japan; stateProvince: Iwate; locality: Kubo River; locationRemarks: the middle reach; **Identification:** identifiedBy: Yusuke Miyazaki; dateIdentified: 2013; **Event:** year: 2009; month: 5; day: 3; **Record Level:** basisOfRecord: PreservedSpecimen
- **Type status:** Other material. **Occurrence:** catalogNumber: KPM-NI 23984; recordedBy: Yusuke Miyazaki; individualCount: 1; **Taxon:** scientificName: Carassius auratus buergeri; **Location:** country: Japan; stateProvince: Iwate; locality: irrigation pond of the Kubo River Basin; verbatimLatitude: 38°55′14″N; verbatimLongitude: 141°01′13″E; **Identification:** identifiedBy: Yusuke Miyazaki; dateIdentified: 2013; **Event:** year: 2009; month: 5; day: 19; **Record Level:** basisOfRecord: PreservedSpecimen
- **Type status:** Other material. **Occurrence:** catalogNumber: KPM-NI 23985; recordedBy: Yusuke Miyazaki; individualCount: 1; **Taxon:** scientificName: Carassius auratus buergeri; **Location:** country: Japan; stateProvince: Iwate; locality: irrigation pond of the Kubo River Basin; verbatimLatitude: 38°55′14″N; verbatimLongitude: 141°01′13″E; **Identification:** identifiedBy: Yusuke Miyazaki; dateIdentified: 2013; **Event:** year: 2009; month: 5; day: 19; **Record Level:** basisOfRecord: PreservedSpecimen
- **Type status:** Other material. **Occurrence:** catalogNumber: KPM-NI 23986; recordedBy: Yusuke Miyazaki; individualCount: 1; **Taxon:** scientificName: Carassius auratus buergeri; **Location:** country: Japan; stateProvince: Iwate; locality: irrigation pond of the Tochikura River Basin; verbatimLatitude: 38°54′22″N; verbatimLongitude: 141°00′46″E; **Identification:** identifiedBy: Yusuke Miyazaki; dateIdentified: 2013; **Event:** year: 2009; month: 5; day: 20; **Record Level:** basisOfRecord: PreservedSpecimen
- **Type status:** Other material. **Occurrence:** catalogNumber: KPM-NI 24397; recordedBy: Yusuke Miyazaki; individualCount: 1; **Taxon:** scientificName: Carassius auratus buergeri; **Location:** country: Japan; stateProvince: Iwate; locality: irrigation pond of the Kubo River Basin; verbatimLatitude: 38°55′28″N; verbatimLongitude: 141°00′02″E; **Identification:** identifiedBy: Yusuke Miyazaki; dateIdentified: 2013; **Event:** year: 2009; month: 9; day: 24; **Record Level:** basisOfRecord: PreservedSpecimen
- **Type status:** Other material. **Occurrence:** catalogNumber: KPM-NI 24414; recordedBy: Yusuke Miyazaki; individualCount: 1; **Taxon:** scientificName: Carassius auratus buergeri; **Location:** country: Japan; stateProvince: Iwate; locality: channel of rice paddy, Tochikura River Basin; verbatimLatitude: 38°54′31″N; verbatimLongitude: 141°00′48″E; **Identification:** identifiedBy: Yusuke Miyazaki; dateIdentified: 2013; **Event:** year: 2009; month: 9; day: 21; **Record Level:** basisOfRecord: PreservedSpecimen
- **Type status:** Other material. **Occurrence:** catalogNumber: KPM-NI 24415; recordedBy: Yusuke Miyazaki; individualCount: 1; **Taxon:** scientificName: Carassius auratus buergeri; **Location:** country: Japan; stateProvince: Iwate; locality: channel of rice paddy, Tochikura River Basin; verbatimLatitude: 38°54′31″N; verbatimLongitude: 141°00′48″E; **Identification:** identifiedBy: Yusuke Miyazaki; dateIdentified: 2013; **Event:** year: 2009; month: 9; day: 21; **Record Level:** basisOfRecord: PreservedSpecimen
- **Type status:** Other material. **Occurrence:** catalogNumber: KPM-NI 24473; recordedBy: Yusuke Miyazaki; individualCount: 1; **Taxon:** scientificName: Carassius auratus buergeri; **Location:** country: Japan; stateProvince: Iwate; municipality: Genbi Town; verbatimLatitude: 38°56′07″N; verbatimLongitude: 141°01′42″E; **Identification:** identifiedBy: Yusuke Miyazaki; dateIdentified: 2013; **Event:** year: 2009; month: 9; day: 22; **Record Level:** basisOfRecord: PreservedSpecimen
- **Type status:** Other material. **Occurrence:** catalogNumber: KPM-NI 24474; recordedBy: Yusuke Miyazaki; individualCount: 1; **Taxon:** scientificName: Carassius auratus buergeri; **Location:** country: Japan; stateProvince: Iwate; municipality: Genbi Town; verbatimLatitude: 38°56′07″N; verbatimLongitude: 141°01′42″E; **Identification:** identifiedBy: Yusuke Miyazaki; dateIdentified: 2013; **Event:** year: 2009; month: 9; day: 22; **Record Level:** basisOfRecord: PreservedSpecimen
- **Type status:** Other material. **Occurrence:** catalogNumber: KPM-NI 30998; recordedBy: Yusuke Miyazaki; individualCount: 1; **Taxon:** scientificName: Carassius auratus buergeri; **Location:** country: Japan; stateProvince: Iwate; locality: irrigation pond of the Kubo River Basin; verbatimLatitude: 38°55′49″N; verbatimLongitude: 141°01′33″E; **Identification:** identifiedBy: Yusuke Miyazaki; dateIdentified: 2013; **Event:** year: 2010; month: 9; day: 5; **Record Level:** basisOfRecord: PreservedSpecimen
- **Type status:** Other material. **Occurrence:** catalogNumber: KPM-NI 30999; recordedBy: Yusuke Miyazaki; individualCount: 1; **Taxon:** scientificName: Carassius auratus buergeri; **Location:** country: Japan; stateProvince: Iwate; locality: irrigation pond of the Kubo River Basin; verbatimLatitude: 38°55′49″N; verbatimLongitude: 141°01′33″E; **Identification:** identifiedBy: Yusuke Miyazaki; dateIdentified: 2013; **Event:** year: 2010; month: 9; day: 5; **Record Level:** basisOfRecord: PreservedSpecimen
- **Type status:** Other material. **Occurrence:** catalogNumber: KPM-NI 31003; recordedBy: Yusuke Miyazaki; individualCount: 1; **Taxon:** scientificName: Carassius auratus buergeri; **Location:** country: Japan; stateProvince: Iwate; locality: irrigation pond of the Kubo River Basin; verbatimLatitude: 38°55′49″N; verbatimLongitude: 141°01′33″E; **Identification:** identifiedBy: Yusuke Miyazaki; dateIdentified: 2013; **Event:** year: 2010; month: 9; day: 4; **Record Level:** basisOfRecord: PreservedSpecimen
- **Type status:** Other material. **Occurrence:** catalogNumber: KPM-NI 31005; recordedBy: Yusuke Miyazaki; individualCount: 1; **Taxon:** scientificName: Carassius auratus buergeri; **Location:** country: Japan; stateProvince: Iwate; locality: irrigation pond of the Kubo River Basin; verbatimLatitude: 38°56′16″N; verbatimLongitude: 141°01′00″E; **Identification:** identifiedBy: Yusuke Miyazaki; dateIdentified: 2013; **Event:** year: 2010; month: 9; day: 4; **Record Level:** basisOfRecord: PreservedSpecimen
- **Type status:** Other material. **Occurrence:** catalogNumber: KPM-NI 31006; recordedBy: Yusuke Miyazaki; individualCount: 1; **Taxon:** scientificName: Carassius auratus buergeri; **Location:** country: Japan; stateProvince: Iwate; locality: irrigation pond of the Kubo River Basin; verbatimLatitude: 38°56′16″N; verbatimLongitude: 141°01′00″E; **Identification:** identifiedBy: Yusuke Miyazaki; dateIdentified: 2013; **Event:** year: 2010; month: 9; day: 4; **Record Level:** basisOfRecord: PreservedSpecimen
- **Type status:** Other material. **Occurrence:** catalogNumber: KPM-NI 31007; recordedBy: Yusuke Miyazaki; individualCount: 1; **Taxon:** scientificName: Carassius auratus buergeri; **Location:** country: Japan; stateProvince: Iwate; locality: irrigation pond of the Kubo River Basin; verbatimLatitude: 38°56′16″N; verbatimLongitude: 141°01′00″E; **Identification:** identifiedBy: Yusuke Miyazaki; dateIdentified: 2013; **Event:** year: 2010; month: 9; day: 4; **Record Level:** basisOfRecord: PreservedSpecimen
- **Type status:** Other material. **Occurrence:** catalogNumber: KPM-NI 31009; recordedBy: Yusuke Miyazaki; individualCount: 1; **Taxon:** scientificName: Carassius auratus buergeri; **Location:** country: Japan; stateProvince: Iwate; locality: irrigation pond of the Kubo River Basin; verbatimLatitude: 38°56′16″N; verbatimLongitude: 141°01′00″E; **Identification:** identifiedBy: Yusuke Miyazaki; dateIdentified: 2013; **Event:** year: 2010; month: 9; day: 4; **Record Level:** basisOfRecord: PreservedSpecimen
- **Type status:** Other material. **Occurrence:** catalogNumber: KPM-NI 31011; recordedBy: Yusuke Miyazaki; individualCount: 1; **Taxon:** scientificName: Carassius auratus buergeri; **Location:** country: Japan; stateProvince: Iwate; locality: irrigation pond of the Kubo River Basin; verbatimLatitude: 38°56′16″N; verbatimLongitude: 141°01′00″E; **Identification:** identifiedBy: Yusuke Miyazaki; dateIdentified: 2013; **Event:** year: 2010; month: 9; day: 4; **Record Level:** basisOfRecord: PreservedSpecimen
- **Type status:** Other material. **Occurrence:** catalogNumber: NSMT-P 96058; recordedBy: Yusuke Miyazaki; individualCount: 1; **Taxon:** scientificName: Carassius auratus buergeri; **Location:** country: Japan; stateProvince: Iwate; locality: irrigation pond of the Kubo River Basin; verbatimLatitude: 38°55′39″N; verbatimLongitude: 140°59′52″E; **Identification:** identifiedBy: Yusuke Miyazaki; dateIdentified: 2013; **Event:** year: 2008; month: 8; day: 30; **Record Level:** basisOfRecord: PreservedSpecimen
- **Type status:** Other material. **Occurrence:** catalogNumber: NSMT-P 96837; recordedBy: Yusuke Miyazaki; individualCount: 1; **Taxon:** scientificName: Carassius auratus buergeri; **Location:** country: Japan; stateProvince: Iwate; locality: irrigation pond of the Ichinono River Basin; **Identification:** identifiedBy: Yusuke Miyazaki; dateIdentified: 2013; **Event:** year: 2008; month: 10; day: 9; **Record Level:** basisOfRecord: PreservedSpecimen
- **Type status:** Other material. **Occurrence:** catalogNumber: NSMT-P 96838; recordedBy: Yusuke Miyazaki; individualCount: 33; **Taxon:** scientificName: Carassius auratus buergeri; **Location:** country: Japan; stateProvince: Iwate; locality: irrigation pond of the Ichinono River Basin; **Identification:** identifiedBy: Yusuke Miyazaki; dateIdentified: 2013; **Event:** year: 2008; month: 10; day: 9; **Record Level:** basisOfRecord: PreservedSpecimen
- **Type status:** Other material. **Occurrence:** catalogNumber: NSMT-P 96890; recordedBy: Yusuke Miyazaki and Yoko Takata; individualCount: 1; **Taxon:** scientificName: Carassius auratus buergeri; **Location:** country: Japan; stateProvince: Iwate; municipality: Hagishou; **Identification:** identifiedBy: Yusuke Miyazaki; dateIdentified: 2013; **Event:** year: 2008; month: 10; day: 8; **Record Level:** basisOfRecord: PreservedSpecimen
- **Type status:** Other material. **Occurrence:** catalogNumber: NSMT-P 96891; recordedBy: Yusuke Miyazaki; individualCount: 1; **Taxon:** scientificName: Carassius auratus buergeri; **Location:** country: Japan; stateProvince: Iwate; locality: irrigation pond of the Tochikura River Basin; **Identification:** identifiedBy: Yusuke Miyazaki; dateIdentified: 2013; **Event:** year: 2008; month: 10; day: 10; **Record Level:** basisOfRecord: PreservedSpecimen
- **Type status:** Other material. **Occurrence:** catalogNumber: NSMT-P 96892; recordedBy: Yusuke Miyazaki; individualCount: 2; **Taxon:** scientificName: Carassius auratus buergeri; **Location:** country: Japan; stateProvince: Iwate; locality: irrigation pond of the Tochikura River Basin; **Identification:** identifiedBy: Yusuke Miyazaki; dateIdentified: 2013; **Event:** year: 2008; month: 10; day: 10; **Record Level:** basisOfRecord: PreservedSpecimen
- **Type status:** Other material. **Occurrence:** catalogNumber: NSMT-P 96901; recordedBy: Yusuke Miyazaki; individualCount: 1; **Taxon:** scientificName: Carassius auratus buergeri; **Location:** country: Japan; stateProvince: Iwate; locality: irrigation pond of the Kubo River Basin; **Identification:** identifiedBy: Yusuke Miyazaki; dateIdentified: 2013; **Event:** year: 2008; month: 10; day: 8; **Record Level:** basisOfRecord: PreservedSpecimen
- **Type status:** Other material. **Occurrence:** catalogNumber: NSMT-P 97045; recordedBy: Yusuke Miyazaki and Yoko Takata; individualCount: 8; **Taxon:** scientificName: Carassius auratus buergeri; **Location:** country: Japan; stateProvince: Iwate; municipality: Hagishou; **Identification:** identifiedBy: Yusuke Miyazaki; dateIdentified: 2013; **Event:** year: 2008; month: 10; day: 9; **Record Level:** basisOfRecord: PreservedSpecimen

##### Ecological interactions

###### Conservation status

National: VU (Ministry of the Environment, Japan 2013); Prefectural: NT (Iwate Prefecture 2014).

##### Distribution

Japan

##### Notes

This taxon is identical to *Carassius buergeri* subsp. 2 of Hosoya (2013). Hosoya (2002) treated this species as a subspecies of *C. auratus* based on genetics, ecological features and comparison with the types in RMNH (but this work is still under preparation). This taxon was mostly collected from lentic environments, but occasionally from lotic environments.

#### Rhodeus ocellatus ocellatus

(Kner, 1866)

##### Materials

- **Type status:** Other material. **Occurrence:** catalogNumber: KPM-NI 22258; recordedBy: Yusuke Miyazaki; individualCount: 1; **Taxon:** scientificName: Rhodeus ocellatus ocellatus; **Location:** country: Japan; stateProvince: Iwate; locality: Kubo River; verbatimLatitude: 38°54′55.4″N; verbatimLongitude: 141°04′23.7″E; **Identification:** identifiedBy: Yusuke Miyazaki; dateIdentified: 2008; **Event:** year: 2008; month: 7; day: 8; **Record Level:** basisOfRecord: PreservedSpecimen
- **Type status:** Other material. **Occurrence:** catalogNumber: KPM-NI 23982; recordedBy: Yusuke Miyazaki; individualCount: 1; **Taxon:** scientificName: Rhodeus ocellatus ocellatus; **Location:** country: Japan; stateProvince: Iwate; verbatimLatitude: 38°55′14″N; verbatimLongitude: 141°01′13″E; **Identification:** identifiedBy: Yusuke Miyazaki; dateIdentified: 2009; **Event:** year: 2009; month: 5; day: 19; **Record Level:** basisOfRecord: PreservedSpecimen
- **Type status:** Other material. **Occurrence:** catalogNumber: KPM-NI 23983; recordedBy: Yusuke Miyazaki; individualCount: 1; **Taxon:** scientificName: Rhodeus ocellatus ocellatus; **Location:** country: Japan; stateProvince: Iwate; locality: irrigation pond of the Kubo River Basin; verbatimLatitude: 38°55′14″N; verbatimLongitude: 141°01′13″E; **Identification:** identifiedBy: Yusuke Miyazaki; dateIdentified: 2009; **Event:** year: 2009; month: 5; day: 19; **Record Level:** basisOfRecord: PreservedSpecimen
- **Type status:** Other material. **Occurrence:** catalogNumber: NSMT-P 96898; recordedBy: Yusuke Miyazaki and Yoko Takata; individualCount: 1; **Taxon:** scientificName: Rhodeus ocellatus ocellatus; **Location:** country: Japan; stateProvince: Iwate; locality: Kubo River; **Identification:** identifiedBy: Yoko Takata; dateIdentified: 2010; **Event:** year: 2008; month: 10; day: 10; **Record Level:** basisOfRecord: PreservedSpecimen

##### Ecological interactions

###### Native status

Non-native (100 of the Japanese Worst Invasive Alien Species: Murakami and Washitani 2002).

##### Distribution

China and Korea.

#### Zacco platypus

(Temminck & Schlegel, 1846)

##### Materials

- **Type status:** Other material. **Occurrence:** catalogNumber: KPM-NI 22257; recordedBy: Yusuke Miyazaki; individualCount: 1; **Taxon:** scientificName: Zacco platypus; **Location:** country: Japan; stateProvince: Iwate; locality: Tochikura River; verbatimLatitude: 38°54′29.3″N; verbatimLongitude: 141°02′52.8″E; **Identification:** identifiedBy: Yusuke Miyazaki; dateIdentified: 2008; **Event:** year: 2008; month: 7; day: 9; **Record Level:** basisOfRecord: PreservedSpecimen
- **Type status:** Other material. **Occurrence:** catalogNumber: KPM-NI 23994; recordedBy: Yusuke Miyazaki; individualCount: 1; **Taxon:** scientificName: Zacco platypus; **Location:** country: Japan; stateProvince: Iwate; locality: Kubo River; verbatimLatitude: 38°54′55″N; verbatimLongitude: 141°04′24″E; **Identification:** identifiedBy: Yusuke Miyazaki; dateIdentified: 2009; **Event:** year: 2009; month: 5; day: 2; **Record Level:** basisOfRecord: PreservedSpecimen
- **Type status:** Other material. **Occurrence:** catalogNumber: KPM-NI 23995; recordedBy: Yusuke Miyazaki; individualCount: 1; **Taxon:** scientificName: Zacco platypus; **Location:** country: Japan; stateProvince: Iwate; locality: Kubo River; verbatimLatitude: 38°54′55″N; verbatimLongitude: 141°04′24″E; **Identification:** identifiedBy: Yusuke Miyazaki; dateIdentified: 2009; **Event:** year: 2009; month: 5; day: 2; **Record Level:** basisOfRecord: PreservedSpecimen
- **Type status:** Other material. **Occurrence:** catalogNumber: NSMT-P 91200; recordedBy: Yusuke Miyazaki; individualCount: 1; **Taxon:** scientificName: Zacco platypus; **Location:** country: Japan; stateProvince: Iwate; locality: Ichinono River; **Identification:** identifiedBy: Yusuke Miyazaki; dateIdentified: 2008; **Event:** year: 2008; month: 7; day: 6; **Record Level:** basisOfRecord: PreservedSpecimen
- **Type status:** Other material. **Occurrence:** catalogNumber: NSMT-P 91647; recordedBy: Yusuke Miyazaki; individualCount: 4; **Taxon:** scientificName: Zacco platypus; **Location:** country: Japan; stateProvince: Iwate; locality: Tochikura River; **Identification:** identifiedBy: Yusuke Miyazaki; dateIdentified: 2008; **Event:** year: 2008; month: 7; day: 11; **Record Level:** basisOfRecord: PreservedSpecimen
- **Type status:** Other material. **Occurrence:** catalogNumber: NSMT-P 96830; recordedBy: Yusuke Miyazaki and Yoko Takata; individualCount: 1; **Taxon:** scientificName: Zacco platypus; **Location:** country: Japan; stateProvince: Iwate; locality: Tochikura River; **Identification:** identifiedBy: Takashi P. Satoh; dateIdentified: 2010; **Event:** year: 2008; month: 10; day: 9; **Record Level:** basisOfRecord: PreservedSpecimen
- **Type status:** Other material. **Occurrence:** catalogNumber: NSMT-P 96847; recordedBy: Yusuke Miyazaki and Yoko Takata; individualCount: 1; **Taxon:** scientificName: Zacco platypus; **Location:** country: Japan; stateProvince: Iwate; locality: Ichinono River; **Identification:** identifiedBy: Takashi P. Satoh; dateIdentified: 2010; **Event:** year: 2008; month: 10; day: 9; **Record Level:** basisOfRecord: PreservedSpecimen

##### Ecological interactions

###### Native status

Non-native (domestic non-native species in Iwate Prefecture: Matsuzawa and Senou 2008).

##### Distribution

Far East Asia.

##### Notes

This taxon is referred to as *Opsariichthys platypus* by Hosoya (2013). This species was only captured from lotic waters of the rivers in the present study.

#### Rhynchocypris steindachneri

(Sauvage, 1883)

##### Materials

- **Type status:** Other material. **Occurrence:** catalogNumber: KPM-NI 21188; recordedBy: Hiroshi Senou; individualCount: 1; **Taxon:** scientificName: Rhynchocypris steindachneri; **Location:** country: Japan; stateProvince: Iwate; locality: Tochikura River; verbatimLatitude: 38°54′43.05″N; verbatimLongitude: 141°01′24.39″E; **Identification:** identifiedBy: Hiroshi Senou; dateIdentified: 2008; **Event:** year: 2008; month: 5; day: 2; **Record Level:** basisOfRecord: PreservedSpecimen
- **Type status:** Other material. **Occurrence:** catalogNumber: KPM-NI 21189; recordedBy: Hiroshi Senou; individualCount: 1; **Taxon:** scientificName: Rhynchocypris steindachneri; **Location:** country: Japan; stateProvince: Iwate; locality: Tochikura River; verbatimLatitude: 38°54′43.05″N; verbatimLongitude: 141°01′24.39″E; **Identification:** identifiedBy: Hiroshi Senou; dateIdentified: 2008; **Event:** year: 2008; month: 5; day: 2; **Record Level:** basisOfRecord: PreservedSpecimen
- **Type status:** Other material. **Occurrence:** catalogNumber: KPM-NI 21197; recordedBy: Yusuke Miyazaki; individualCount: 1; **Taxon:** scientificName: Rhynchocypris steindachneri; **Location:** country: Japan; stateProvince: Iwate; locality: irrigation pond of the Ichinono River Basin; verbatimLatitude: 38°53′43″N; verbatimLongitude: 140°58′49″E; **Identification:** identifiedBy: Yusuke Miyazaki; dateIdentified: 2008; **Event:** year: 2008; month: 5; day: 5; **Record Level:** basisOfRecord: PreservedSpecimen
- **Type status:** Other material. **Occurrence:** catalogNumber: KPM-NI 21198; recordedBy: Yusuke Miyazaki; individualCount: 1; **Taxon:** scientificName: Rhynchocypris steindachneri; **Location:** country: Japan; stateProvince: Iwate; locality: irrigation pond of the Ichinono River Basin; verbatimLatitude: 38°53′43″N; verbatimLongitude: 140°58′49″E; **Identification:** identifiedBy: Yusuke Miyazaki; dateIdentified: 2008; **Event:** year: 2008; month: 5; day: 5; **Record Level:** basisOfRecord: PreservedSpecimen
- **Type status:** Other material. **Occurrence:** catalogNumber: KPM-NI 21220; recordedBy: Hiroshi Senou and Takumi Senou; individualCount: 2; **Taxon:** scientificName: Rhynchocypris steindachneri; **Location:** country: Japan; stateProvince: Iwate; locality: Tochikura River; verbatimLatitude: 38°54′58.75″N; verbatimLongitude: 140°58′49.07″E; **Identification:** identifiedBy: Hiroshi Senou; dateIdentified: 2008; **Event:** year: 2008; month: 5; day: 2; **Record Level:** basisOfRecord: PreservedSpecimen
- **Type status:** Other material. **Occurrence:** catalogNumber: KPM-NI 21398; recordedBy: Shin-ichi Suda and Yusuke Miyazaki; individualCount: 2; **Taxon:** scientificName: Rhynchocypris steindachneri; **Location:** country: Japan; stateProvince: Iwate; locality: Ichinono River; verbatimLatitude: 38°54′19″N; verbatimLongitude: 141°02′41″E; **Identification:** identifiedBy: Yusuke Miyazaki; dateIdentified: 2008; **Event:** year: 2008; month: 5; day: 3; **Record Level:** basisOfRecord: PreservedSpecimen
- **Type status:** Other material. **Occurrence:** catalogNumber: KPM-NI 22276; recordedBy: Yusuke Miyazaki; individualCount: 1; **Taxon:** scientificName: Rhynchocypris steindachneri; **Location:** country: Japan; stateProvince: Iwate; locality: irrigation pond of the Ichinono River Basin; verbatimLatitude: 38°53′43.8″N; verbatimLongitude: 140°58′42.4″E; **Identification:** identifiedBy: Yusuke Miyazaki; dateIdentified: 2008; **Event:** year: 2008; month: 9; day: 16; **Record Level:** basisOfRecord: PreservedSpecimen
- **Type status:** Other material. **Occurrence:** catalogNumber: KPM-NI 23654; recordedBy: Yusuke Miyazaki; individualCount: 1; **Taxon:** scientificName: Rhynchocypris steindachneri; **Location:** country: Japan; stateProvince: Iwate; locality: Kubo River; verbatimLatitude: 38°54′55.4″N; verbatimLongitude: 141°04′23.7″E; **Identification:** identifiedBy: Yusuke Miyazaki; dateIdentified: 2009; **Event:** year: 2008; month: 7; day: 9; **Record Level:** basisOfRecord: PreservedSpecimen
- **Type status:** Other material. **Occurrence:** catalogNumber: KPM-NI 23655; recordedBy: Yusuke Miyazaki; individualCount: 1; **Taxon:** scientificName: Rhynchocypris steindachneri; **Location:** country: Japan; stateProvince: Iwate; locality: Kubo River; verbatimLatitude: 38°54′55.4″N; verbatimLongitude: 141°04′23.7″E; **Identification:** identifiedBy: Yusuke Miyazaki; dateIdentified: 2009; **Event:** year: 2008; month: 7; day: 9; **Record Level:** basisOfRecord: PreservedSpecimen
- **Type status:** Other material. **Occurrence:** catalogNumber: KPM-NI 23656; recordedBy: Yusuke Miyazaki; individualCount: 1; **Taxon:** scientificName: Rhynchocypris steindachneri; **Location:** country: Japan; stateProvince: Iwate; locality: Kubo River; verbatimLatitude: 38°54′55.4″N; verbatimLongitude: 141°04′23.7″E; **Identification:** identifiedBy: Yusuke Miyazaki; dateIdentified: 2009; **Event:** year: 2008; month: 7; day: 9; **Record Level:** basisOfRecord: PreservedSpecimen
- **Type status:** Other material. **Occurrence:** catalogNumber: KPM-NI 23657; recordedBy: Yusuke Miyazaki; individualCount: 1; **Taxon:** scientificName: Rhynchocypris steindachneri; **Location:** country: Japan; stateProvince: Iwate; locality: Ichinono River; verbatimLatitude: 38°54′17.9″N; verbatimLongitude: 141°02′42.1″E; **Identification:** identifiedBy: Yusuke Miyazaki; dateIdentified: 2009; **Event:** year: 2008; month: 7; day: 5; **Record Level:** basisOfRecord: PreservedSpecimen
- **Type status:** Other material. **Occurrence:** catalogNumber: KPM-NI 23658; recordedBy: Yusuke Miyazaki; individualCount: 1; **Taxon:** scientificName: Rhynchocypris steindachneri; **Location:** country: Japan; stateProvince: Iwate; locality: Ichinono River; verbatimLatitude: 38°54′17.9″N; verbatimLongitude: 141°02′42.1″E; **Identification:** identifiedBy: Yusuke Miyazaki; dateIdentified: 2009; **Event:** year: 2008; month: 7; day: 5; **Record Level:** basisOfRecord: PreservedSpecimen
- **Type status:** Other material. **Occurrence:** catalogNumber: KPM-NI 23659; recordedBy: Yusuke Miyazaki; individualCount: 1; **Taxon:** scientificName: Rhynchocypris steindachneri; **Location:** country: Japan; stateProvince: Iwate; locality: Ichinono River; verbatimLatitude: 38°54′17.9″N; verbatimLongitude: 141°02′42.1″E; **Identification:** identifiedBy: Yusuke Miyazaki; dateIdentified: 2009; **Event:** year: 2008; month: 7; day: 5; **Record Level:** basisOfRecord: PreservedSpecimen
- **Type status:** Other material. **Occurrence:** catalogNumber: KPM-NI 23989; recordedBy: Yusuke Miyazaki; individualCount: 1; **Taxon:** scientificName: Rhynchocypris steindachneri; **Location:** country: Japan; stateProvince: Iwate; locality: irrigation pond of the Kubo River Basin; verbatimLatitude: 38°55′14″N; verbatimLongitude: 141°01′13″E; **Identification:** identifiedBy: Yusuke Miyazaki; dateIdentified: 2009; **Event:** year: 2009; month: 5; day: 3; **Record Level:** basisOfRecord: PreservedSpecimen
- **Type status:** Other material. **Occurrence:** catalogNumber: KPM-NI 23990; recordedBy: Yuichiro Sekizaki; individualCount: 1; **Taxon:** scientificName: Rhynchocypris steindachneri; **Location:** country: Japan; stateProvince: Iwate; locality: irrigation pond of the Kubo River Basin; verbatimLatitude: 38°55′39″N; verbatimLongitude: 140°59′52″E; **Identification:** identifiedBy: Yusuke Miyazaki; dateIdentified: 2009; **Event:** year: 2009; month: 8; day: 5; **Record Level:** basisOfRecord: PreservedSpecimen
- **Type status:** Other material. **Occurrence:** catalogNumber: KPM-NI 24000; recordedBy: Yusuke Miyazaki; individualCount: 1; **Taxon:** scientificName: Rhynchocypris steindachneri; **Location:** country: Japan; stateProvince: Iwate; locality: irrigation pond of the Tochikura River Basin; verbatimLatitude: 38°55′05″N; verbatimLongitude: 140°58′20″E; **Identification:** identifiedBy: Yusuke Miyazaki; dateIdentified: 2009; **Event:** year: 2009; month: 5; day: 2; **Record Level:** basisOfRecord: PreservedSpecimen
- **Type status:** Other material. **Occurrence:** catalogNumber: KPM-NI 24419; recordedBy: Yusuke Miyazaki; individualCount: 1; **Taxon:** scientificName: Rhynchocypris steindachneri; **Location:** country: Japan; stateProvince: Iwate; locality: irrigation pond of the Kubo River Basin; verbatimLatitude: 38°56′33″N; verbatimLongitude: 140°59′49″E; **Identification:** identifiedBy: Yusuke Miyazaki; dateIdentified: 2009; **Event:** year: 2009; month: 9; day: 22; **Record Level:** basisOfRecord: PreservedSpecimen
- **Type status:** Other material. **Occurrence:** catalogNumber: NSMT-P 91185; recordedBy: Yusuke Miyazaki; individualCount: 5; **Taxon:** scientificName: Rhynchocypris steindachneri; **Location:** country: Japan; stateProvince: Iwate; locality: Kubo River; **Identification:** identifiedBy: Yusuke Miyazaki; dateIdentified: 2008; **Event:** year: 2008; month: 7; day: 2; **Record Level:** basisOfRecord: PreservedSpecimen
- **Type status:** Other material. **Occurrence:** catalogNumber: NSMT-P 91188; recordedBy: Yusuke Miyazaki; individualCount: 1; **Taxon:** scientificName: Rhynchocypris steindachneri; **Location:** country: Japan; stateProvince: Iwate; locality: Kubo River; **Identification:** identifiedBy: Yusuke Miyazaki; dateIdentified: 2008; **Event:** year: 2008; month: 7; day: 3; **Record Level:** basisOfRecord: PreservedSpecimen
- **Type status:** Other material. **Occurrence:** catalogNumber: NSMT-P 91191; recordedBy: Yusuke Miyazaki; individualCount: 6; **Taxon:** scientificName: Rhynchocypris steindachneri; **Location:** country: Japan; stateProvince: Iwate; locality: Kubo River; **Identification:** identifiedBy: Yusuke Miyazaki; dateIdentified: 2008; **Event:** year: 2008; month: 7; day: 2; **Record Level:** basisOfRecord: PreservedSpecimen
- **Type status:** Other material. **Occurrence:** catalogNumber: NSMT-P 91192; recordedBy: Yusuke Miyazaki; individualCount: 4; **Taxon:** scientificName: Rhynchocypris steindachneri; **Location:** country: Japan; stateProvince: Iwate; locality: Tochikura River; **Identification:** identifiedBy: Yusuke Miyazaki; dateIdentified: 2008; **Event:** year: 2008; month: 7; day: 4; **Record Level:** basisOfRecord: PreservedSpecimen
- **Type status:** Other material. **Occurrence:** catalogNumber: NSMT-P 91198; recordedBy: Yusuke Miyazaki; individualCount: 1; **Taxon:** scientificName: Rhynchocypris steindachneri; **Location:** country: Japan; stateProvince: Iwate; locality: Kubo River; **Identification:** identifiedBy: Yusuke Miyazaki; dateIdentified: 2008; **Event:** year: 2008; month: 7; day: 7; **Record Level:** basisOfRecord: PreservedSpecimen
- **Type status:** Other material. **Occurrence:** catalogNumber: NSMT-P 91201; recordedBy: Yusuke Miyazaki; individualCount: 1; **Taxon:** scientificName: Rhynchocypris steindachneri; **Location:** country: Japan; stateProvince: Iwate; locality: Ichinono River; **Identification:** identifiedBy: Yusuke Miyazaki; dateIdentified: 2008; **Event:** year: 2008; month: 7; day: 6; **Record Level:** basisOfRecord: PreservedSpecimen
- **Type status:** Other material. **Occurrence:** catalogNumber: NSMT-P 91203; recordedBy: Yusuke Miyazaki; individualCount: 1; **Taxon:** scientificName: Rhynchocypris steindachneri; **Location:** country: Japan; stateProvince: Iwate; locality: Tochikura River; **Identification:** identifiedBy: Yusuke Miyazaki; dateIdentified: 2008; **Event:** year: 2008; month: 7; day: 8; **Record Level:** basisOfRecord: PreservedSpecimen
- **Type status:** Other material. **Occurrence:** catalogNumber: NSMT-P 91206; recordedBy: Yusuke Miyazaki; individualCount: 4; **Taxon:** scientificName: Rhynchocypris steindachneri; **Location:** country: Japan; stateProvince: Iwate; locality: Tochikura River; **Identification:** identifiedBy: Yusuke Miyazaki; dateIdentified: 2008; **Event:** year: 2008; month: 7; day: 5; **Record Level:** basisOfRecord: PreservedSpecimen
- **Type status:** Other material. **Occurrence:** catalogNumber: NSMT-P 91207; recordedBy: Yusuke Miyazaki; individualCount: 1; **Taxon:** scientificName: Rhynchocypris steindachneri; **Location:** country: Japan; stateProvince: Iwate; locality: Ichinono River; **Identification:** identifiedBy: Yusuke Miyazaki; dateIdentified: 2008; **Event:** year: 2008; month: 7; day: 10; **Record Level:** basisOfRecord: PreservedSpecimen
- **Type status:** Other material. **Occurrence:** catalogNumber: NSMT-P 91638; recordedBy: Yusuke Miyazaki; individualCount: 2; **Taxon:** scientificName: Rhynchocypris steindachneri; **Location:** country: Japan; stateProvince: Iwate; locality: Ichinono River; **Identification:** identifiedBy: Yusuke Miyazaki; dateIdentified: 2008; **Event:** year: 2008; month: 7; day: 7; **Record Level:** basisOfRecord: PreservedSpecimen
- **Type status:** Other material. **Occurrence:** catalogNumber: NSMT-P 91643; recordedBy: Yusuke Miyazaki; individualCount: 1; **Taxon:** scientificName: Rhynchocypris steindachneri; **Location:** country: Japan; stateProvince: Iwate; locality: Tochikura River; **Identification:** identifiedBy: Yusuke Miyazaki; dateIdentified: 2008; **Event:** year: 2008; month: 7; day: 9; **Record Level:** basisOfRecord: PreservedSpecimen
- **Type status:** Other material. **Occurrence:** catalogNumber: NSMT-P 91649; recordedBy: Yusuke Miyazaki; individualCount: 1; **Taxon:** scientificName: Rhynchocypris steindachneri; **Location:** country: Japan; stateProvince: Iwate; locality: irrigation pond of the Kubo River Basin; **Identification:** identifiedBy: Yusuke Miyazaki; dateIdentified: 2008; **Event:** year: 2008; month: 7; day: 12; **Record Level:** basisOfRecord: PreservedSpecimen
- **Type status:** Other material. **Occurrence:** catalogNumber: NSMT-P 91650; recordedBy: Yusuke Miyazaki; individualCount: 1; **Taxon:** scientificName: Rhynchocypris steindachneri; **Location:** country: Japan; stateProvince: Iwate; locality: Kubo River; **Identification:** identifiedBy: Yusuke Miyazaki; dateIdentified: 2008; **Event:** year: 1967; month: 1; day: 11; **Record Level:** basisOfRecord: PreservedSpecimen
- **Type status:** Other material. **Occurrence:** catalogNumber: NSMT-P 96057; recordedBy: Yusuke Miyazaki; individualCount: 1; **Taxon:** scientificName: Rhynchocypris steindachneri; **Location:** country: Japan; stateProvince: Iwate; locality: irrigation pond of the Kubo River Basin; verbatimLatitude: 38°55′39″N; verbatimLongitude: 140°59′52″E; **Identification:** identifiedBy: Yusuke Miyazaki; dateIdentified: 2009; **Event:** year: 2008; month: 8; day: 30; **Record Level:** basisOfRecord: PreservedSpecimen
- **Type status:** Other material. **Occurrence:** catalogNumber: NSMT-P 96839; recordedBy: Yusuke Miyazaki and Yoko Takata; individualCount: 1; **Taxon:** scientificName: Rhynchocypris steindachneri; **Location:** country: Japan; stateProvince: Iwate; locality: Tochikura River; **Identification:** identifiedBy: Yoko Takata; dateIdentified: 2010; **Event:** year: 2008; month: 10; day: 8; **Record Level:** basisOfRecord: PreservedSpecimen
- **Type status:** Other material. **Occurrence:** catalogNumber: NSMT-P 96840; recordedBy: Yusuke Miyazaki and Yoko Takata; individualCount: 3; **Taxon:** scientificName: Rhynchocypris steindachneri; **Location:** country: Japan; stateProvince: Iwate; locality: Ichinono River; **Identification:** identifiedBy: Yoko Takata; dateIdentified: 2010; **Event:** year: 2008; month: 10; day: 8; **Record Level:** basisOfRecord: PreservedSpecimen
- **Type status:** Other material. **Occurrence:** catalogNumber: NSMT-P 96844; recordedBy: Yusuke Miyazaki and Yoko Takata; individualCount: 4; **Taxon:** scientificName: Rhynchocypris steindachneri; **Location:** country: Japan; stateProvince: Iwate; locality: Kubo River; **Identification:** identifiedBy: Yoko Takata; dateIdentified: 2010; **Event:** year: 2008; month: 10; day: 9; **Record Level:** basisOfRecord: PreservedSpecimen

##### Distribution

Japan

##### Notes

We do not follow the Catalog of Fishes, but follow Kottelat (2006) in the nomenclature of this species. This taxon is identical with *Phoxinus lagowskii steindachneri* of Hosoya (2013), and was recorded from lotic waters and the inlets to some lentic waters.

#### Tribolodon hakonensis

(Günther, 1880)

##### Materials

- **Type status:** Other material. **Occurrence:** catalogNumber: KPM-NI 21187; recordedBy: Hiroshi Senou; individualCount: 1; **Taxon:** scientificName: Tribolodon hakonensis; **Location:** country: Japan; stateProvince: Iwate; locality: Tochikura River; verbatimLatitude: 38°54′43.05″N; verbatimLongitude: 141°01′24.39″E; **Identification:** identifiedBy: Hiroshi Senou; dateIdentified: 2008; **Event:** year: 2008; month: 5; day: 2; **Record Level:** basisOfRecord: PreservedSpecimen
- **Type status:** Other material. **Occurrence:** catalogNumber: KPM-NI 22254; recordedBy: Yusuke Miyazaki; individualCount: 1; **Taxon:** scientificName: Tribolodon hakonensis; **Location:** country: Japan; stateProvince: Iwate; locality: Tochikura River; verbatimLatitude: 38°54′43.6″N; verbatimLongitude: 141°01′25.1″E; **Identification:** identifiedBy: Yusuke Miyazaki; dateIdentified: 2008; **Event:** year: 2008; month: 7; day: 5; **Record Level:** basisOfRecord: PreservedSpecimen
- **Type status:** Other material. **Occurrence:** catalogNumber: KPM-NI 22260; recordedBy: Yusuke Miyazaki; individualCount: 1; **Taxon:** scientificName: Tribolodon hakonensis; **Location:** country: Japan; stateProvince: Iwate; locality: Kubo River; verbatimLatitude: 38°56′01.6″N; verbatimLongitude: 141°01′01.0″E; **Identification:** identifiedBy: Yusuke Miyazaki; dateIdentified: 2008; **Event:** year: 2008; month: 7; day: 11; **Record Level:** basisOfRecord: PreservedSpecimen
- **Type status:** Other material. **Occurrence:** catalogNumber: KPM-NI 23643; recordedBy: Yusuke Miyazaki; individualCount: 1; **Taxon:** scientificName: Tribolodon hakonensis; **Location:** country: Japan; stateProvince: Iwate; locality: Kubo River; verbatimLatitude: 38°56′01.6″N; verbatimLongitude: 141°01′01.0″E; **Identification:** identifiedBy: Yusuke Miyazaki; dateIdentified: 2008; **Event:** year: 2008; month: 7; day: 11; **Record Level:** basisOfRecord: PreservedSpecimen
- **Type status:** Other material. **Occurrence:** catalogNumber: KPM-NI 23740; recordedBy: Yusuke Miyazaki; individualCount: 1; **Taxon:** scientificName: Tribolodon hakonensis; **Location:** country: Japan; stateProvince: Iwate; locality: Kubo River; verbatimLatitude: 38°53′53.0″N; verbatimLongitude: 141°01′16.8″E; **Identification:** identifiedBy: Hiroshi Senou; dateIdentified: 2009; **Event:** year: 2009; month: 5; day: 3; **Record Level:** basisOfRecord: PreservedSpecimen
- **Type status:** Other material. **Occurrence:** catalogNumber: KPM-NI 23741; recordedBy: Yusuke Miyazaki; individualCount: 1; **Taxon:** scientificName: Tribolodon hakonensis; **Location:** country: Japan; stateProvince: Iwate; locality: Kubo River; verbatimLatitude: 38°56′01.6″N; verbatimLongitude: 141°01′01.0″E; **Identification:** identifiedBy: Yusuke Miyazaki; dateIdentified: 2009; **Event:** year: 2009; month: 5; day: 3; **Record Level:** basisOfRecord: PreservedSpecimen
- **Type status:** Other material. **Occurrence:** catalogNumber: KPM-NI 23993; recordedBy: Yusuke Miyazaki; individualCount: 1; **Taxon:** scientificName: Tribolodon hakonensis; **Location:** country: Japan; stateProvince: Iwate; locality: Tochikura River; verbatimLatitude: 38°54′59″N; verbatimLongitude: 140°58′51″E; **Identification:** identifiedBy: Yusuke Miyazaki; dateIdentified: 2009; **Event:** year: 2009; month: 4; day: 30; **Record Level:** basisOfRecord: PreservedSpecimen
- **Type status:** Other material. **Occurrence:** catalogNumber: NSMT-P 91186; recordedBy: Yusuke Miyazaki; individualCount: 4; **Taxon:** scientificName: Tribolodon hakonensis; **Location:** country: Japan; stateProvince: Iwate; locality: Kubo River; **Identification:** identifiedBy: Yusuke Miyazaki; dateIdentified: 2008; **Event:** year: 2008; month: 7; day: 2; **Record Level:** basisOfRecord: PreservedSpecimen
- **Type status:** Other material. **Occurrence:** catalogNumber: NSMT-P 91187; recordedBy: Yusuke Miyazaki; individualCount: 1; **Taxon:** scientificName: Tribolodon hakonensis; **Location:** country: Japan; stateProvince: Iwate; locality: Kubo River; **Identification:** identifiedBy: Yusuke Miyazaki; dateIdentified: 2008; **Event:** year: 2008; month: 7; day: 2; **Record Level:** basisOfRecord: PreservedSpecimen
- **Type status:** Other material. **Occurrence:** catalogNumber: NSMT-P 91190; recordedBy: Yusuke Miyazaki; individualCount: 1; **Taxon:** scientificName: Tribolodon hakonensis; **Location:** country: Japan; stateProvince: Iwate; locality: Tochikura River; **Identification:** identifiedBy: Yusuke Miyazaki; dateIdentified: 2008; **Event:** year: 2008; month: 7; day: 4; **Record Level:** basisOfRecord: PreservedSpecimen
- **Type status:** Other material. **Occurrence:** catalogNumber: NSMT-P 91204; recordedBy: Yusuke Miyazaki; individualCount: 1; **Taxon:** scientificName: Tribolodon hakonensis; **Location:** country: Japan; stateProvince: Iwate; locality: Tochikura River; **Identification:** identifiedBy: Yusuke Miyazaki; dateIdentified: 2008; **Event:** year: 2008; month: 7; day: 8; **Record Level:** basisOfRecord: PreservedSpecimen
- **Type status:** Other material. **Occurrence:** catalogNumber: NSMT-P 96828; recordedBy: Yusuke Miyazaki and Yoko Takata; individualCount: 1; **Taxon:** scientificName: Tribolodon hakonensis; **Location:** country: Japan; stateProvince: Iwate; locality: Tochikura River; **Identification:** identifiedBy: Takashi P. Satoh; dateIdentified: 2010; **Event:** year: 2008; month: 10; day: 9; **Record Level:** basisOfRecord: PreservedSpecimen
- **Type status:** Other material. **Occurrence:** catalogNumber: NSMT-P 96841; recordedBy: Yusuke Miyazaki and Yoko Takata; individualCount: 1; **Taxon:** scientificName: Tribolodon hakonensis; **Location:** country: Japan; stateProvince: Iwate; locality: Tochikura River; **Identification:** identifiedBy: Yoko Takata; dateIdentified: 2010; **Event:** year: 2008; month: 10; day: 8; **Record Level:** basisOfRecord: PreservedSpecimen
- **Type status:** Other material. **Occurrence:** catalogNumber: NSMT-P 96846; recordedBy: Yusuke Miyazaki and Yoko Takata; individualCount: 3; **Taxon:** scientificName: Tribolodon hakonensis; **Location:** country: Japan; stateProvince: Iwate; locality: Ichinono River; **Identification:** identifiedBy: Takashi P. Satoh; dateIdentified: 2010; **Event:** year: 2008; month: 10; day: 9; **Record Level:** basisOfRecord: PreservedSpecimen

##### Distribution

Korea and Japan.

##### Notes

This species was recorded from fast currents of the lotic environments, but larvae and juveniles were sometimes recorded from slower flowing parts of the rivers.

#### Pseudorasbora parva

(Temminck & Schlegel, 1846)

##### Materials

- **Type status:** Other material. **Occurrence:** catalogNumber: KPM-NI 19450; recordedBy: Yusuke Miyazaki; individualCount: 1; **Taxon:** scientificName: Pseudorasbora parva; **Location:** country: Japan; stateProvince: Iwate; locality: irrigation pond of the Kubo River Basin; verbatimLatitude: 38°55′54″N; verbatimLongitude: 141°01′34″E; **Identification:** identifiedBy: Hiroshi Senou; dateIdentified: 2007; **Event:** year: 2007; month: 9; day: 12; **Record Level:** basisOfRecord: PreservedSpecimen
- **Type status:** Other material. **Occurrence:** catalogNumber: KPM-NI 19451; recordedBy: Yusuke Miyazaki; individualCount: 1; **Taxon:** scientificName: Pseudorasbora parva; **Location:** country: Japan; stateProvince: Iwate; locality: irrigation pond of the Kubo River Basin; verbatimLatitude: 38°55′41″N; verbatimLongitude: 141°01′53″E; **Identification:** identifiedBy: Yusuke Miyazaki; dateIdentified: 2007; **Event:** year: 2007; month: 9; day: 10; **Record Level:** basisOfRecord: PreservedSpecimen
- **Type status:** Other material. **Occurrence:** catalogNumber: KPM-NI 19452; recordedBy: Yusuke Miyazaki; individualCount: 1; **Taxon:** scientificName: Pseudorasbora parva; **Location:** country: Japan; stateProvince: Iwate; locality: irrigation pond of the Kubo River Basin; verbatimLatitude: 38°55′41″N; verbatimLongitude: 141°01′53″E; **Identification:** identifiedBy: Yusuke Miyazaki; dateIdentified: 2007; **Event:** year: 2007; month: 9; day: 10; **Record Level:** basisOfRecord: PreservedSpecimen
- **Type status:** Other material. **Occurrence:** catalogNumber: KPM-NI 21216; recordedBy: Yusuke Miyazaki; individualCount: 1; **Taxon:** scientificName: Pseudorasbora parva; **Location:** country: Japan; stateProvince: Iwate; locality: irrigation pond of the Kubo River Basin; verbatimLatitude: 38°55′55″N; verbatimLongitude: 141°02′03″E; **Identification:** identifiedBy: Yusuke Miyazaki; dateIdentified: 2008; **Event:** year: 2008; month: 5; day: 1; **Record Level:** basisOfRecord: PreservedSpecimen
- **Type status:** Other material. **Occurrence:** catalogNumber: KPM-NI 21413; recordedBy: Yusuke Miyazaki; individualCount: 1; **Taxon:** scientificName: Pseudorasbora parva; **Location:** country: Japan; stateProvince: Iwate; locality: irrigation pond of the Kubo River Basin; verbatimLatitude: 38°55′19″N; verbatimLongitude: 140°59′29″E; **Identification:** identifiedBy: Yusuke Miyazaki; dateIdentified: 2008; **Event:** year: 2008; month: 6; day: 4; **Record Level:** basisOfRecord: PreservedSpecimen
- **Type status:** Other material. **Occurrence:** catalogNumber: KPM-NI 22255; recordedBy: Yusuke Miyazaki; individualCount: 1; **Taxon:** scientificName: Pseudorasbora parva; **Location:** country: Japan; stateProvince: Iwate; locality: Tochikura River; verbatimLatitude: 38°54′43.6″N; verbatimLongitude: 141°01′25.1″E; **Identification:** identifiedBy: Yusuke Miyazaki; dateIdentified: 2008; **Event:** year: 2008; month: 7; day: 5; **Record Level:** basisOfRecord: PreservedSpecimen
- **Type status:** Other material. **Occurrence:** catalogNumber: KPM-NI 22259; recordedBy: Yusuke Miyazaki; individualCount: 1; **Taxon:** scientificName: Pseudorasbora parva; **Location:** country: Japan; stateProvince: Iwate; locality: Kubo River; verbatimLatitude: 38°54′55.4″N; verbatimLongitude: 141°04′23.7″E; **Identification:** identifiedBy: Yusuke Miyazaki; dateIdentified: 2008; **Event:** year: 2008; month: 7; day: 9; **Record Level:** basisOfRecord: PreservedSpecimen
- **Type status:** Other material. **Occurrence:** catalogNumber: KPM-NI 22272; recordedBy: Yusuke Miyazaki; individualCount: 1; **Taxon:** scientificName: Pseudorasbora parva; **Location:** country: Japan; stateProvince: Iwate; locality: irrigation pond of the Kubo River Basin; verbatimLatitude: 38°53′43.8″N; verbatimLongitude: 140°58′42.4″E; **Identification:** identifiedBy: Yusuke Miyazaki; dateIdentified: 2008; **Event:** year: 2008; month: 9; day: 16; **Record Level:** basisOfRecord: PreservedSpecimen
- **Type status:** Other material. **Occurrence:** catalogNumber: KPM-NI 22273; recordedBy: Yusuke Miyazaki; individualCount: 1; **Taxon:** scientificName: Pseudorasbora parva; **Location:** country: Japan; stateProvince: Iwate; locality: irrigation pond of the Kubo River Basin; verbatimLatitude: 38°53′43.8″N; verbatimLongitude: 140°58′42.4″E; **Identification:** identifiedBy: Yusuke Miyazaki; dateIdentified: 2008; **Event:** year: 2008; month: 9; day: 16; **Record Level:** basisOfRecord: PreservedSpecimen
- **Type status:** Other material. **Occurrence:** catalogNumber: KPM-NI 22284; recordedBy: Yusuke Miyazaki; individualCount: 1; **Taxon:** scientificName: Pseudorasbora parva; **Location:** country: Japan; stateProvince: Iwate; locality: irrigation pond of the Kubo River Basin; verbatimLatitude: 38°55′41.3″N; verbatimLongitude: 141°02′06.3″E; **Identification:** identifiedBy: Yusuke Miyazaki; dateIdentified: 2008; **Event:** year: 2008; month: 8; day: 23; **Record Level:** basisOfRecord: PreservedSpecimen
- **Type status:** Other material. **Occurrence:** catalogNumber: KPM-NI 22285; recordedBy: Yusuke Miyazaki; individualCount: 1; **Taxon:** scientificName: Pseudorasbora parva; **Location:** country: Japan; stateProvince: Iwate; locality: irrigation pond of the Kubo River Basin; verbatimLatitude: 38°55′41.3″N; verbatimLongitude: 141°02′06.3″E; **Identification:** identifiedBy: Yusuke Miyazaki; dateIdentified: 2008; **Event:** year: 2008; month: 8; day: 23; **Record Level:** basisOfRecord: PreservedSpecimen
- **Type status:** Other material. **Occurrence:** catalogNumber: KPM-NI 23971; recordedBy: Yusuke Miyazaki; individualCount: 2; **Taxon:** scientificName: Pseudorasbora parva; **Location:** country: Japan; stateProvince: Iwate; locality: irrigation pond of the Kubo River Basin; verbatimLatitude: 38°55′33″N; verbatimLongitude: 141°01′53″E; **Identification:** identifiedBy: Yusuke Miyazaki; dateIdentified: 2009; **Event:** year: 2009; month: 8; day: 2; **Record Level:** basisOfRecord: PreservedSpecimen
- **Type status:** Other material. **Occurrence:** catalogNumber: KPM-NI 23972; recordedBy: Yusuke Miyazaki; individualCount: 1; **Taxon:** scientificName: Pseudorasbora parva; **Location:** country: Japan; stateProvince: Iwate; locality: irrigation pond of the Kubo River Basin; verbatimLatitude: 38°55′49″N; verbatimLongitude: 141°01′56″E; **Identification:** identifiedBy: Yusuke Miyazaki; dateIdentified: 2009; **Event:** year: 2009; month: 7; day: 13; **Record Level:** basisOfRecord: PreservedSpecimen
- **Type status:** Other material. **Occurrence:** catalogNumber: KPM-NI 23973; recordedBy: Yusuke Miyazaki; individualCount: 1; **Taxon:** scientificName: Pseudorasbora parva; **Location:** country: Japan; stateProvince: Iwate; locality: irrigation pond of the Kubo River Basin; verbatimLatitude: 38°55′21″N; verbatimLongitude: 141°03′30″E; **Identification:** identifiedBy: Yusuke Miyazaki; dateIdentified: 2009; **Event:** year: 2009; month: 5; day: 18; **Record Level:** basisOfRecord: PreservedSpecimen
- **Type status:** Other material. **Occurrence:** catalogNumber: KPM-NI 23974; recordedBy: Yusuke Miyazaki; individualCount: 1; **Taxon:** scientificName: Pseudorasbora parva; **Location:** country: Japan; stateProvince: Iwate; locality: irrigation pond of the Kubo River Basin; verbatimLatitude: 38°55′21″N; verbatimLongitude: 141°03′30″E; **Identification:** identifiedBy: Yusuke Miyazaki; dateIdentified: 2009; **Event:** year: 2009; month: 5; day: 18; **Record Level:** basisOfRecord: PreservedSpecimen
- **Type status:** Other material. **Occurrence:** catalogNumber: KPM-NI 23975; recordedBy: Yusuke Miyazaki; individualCount: 1; **Taxon:** scientificName: Pseudorasbora parva; **Location:** country: Japan; stateProvince: Iwate; locality: irrigation pond of the Kubo River Basin; verbatimLatitude: 38°55′21″N; verbatimLongitude: 141°03′30″E; **Identification:** identifiedBy: Yusuke Miyazaki; dateIdentified: 2009; **Event:** year: 2009; month: 5; day: 18; **Record Level:** basisOfRecord: PreservedSpecimen
- **Type status:** Other material. **Occurrence:** catalogNumber: KPM-NI 23976; recordedBy: Yusuke Miyazaki; individualCount: 5; **Taxon:** scientificName: Pseudorasbora parva; **Location:** country: Japan; stateProvince: Iwate; locality: irrigation pond of the Kubo River Basin; verbatimLatitude: 38°55′37″N; verbatimLongitude: 141°01′48″E; **Identification:** identifiedBy: Yusuke Miyazaki; dateIdentified: 2009; **Event:** year: 2009; month: 8; day: 2; **Record Level:** basisOfRecord: PreservedSpecimen
- **Type status:** Other material. **Occurrence:** catalogNumber: KPM-NI 23977; recordedBy: Yusuke Miyazaki; individualCount: 1; **Taxon:** scientificName: Pseudorasbora parva; **Location:** country: Japan; stateProvince: Iwate; locality: irrigation pond of the Kubo River Basin; verbatimLatitude: 38°55′25″N; verbatimLongitude: 141°04′06″E; **Identification:** identifiedBy: Yusuke Miyazaki; dateIdentified: 2009; **Event:** year: 2009; month: 5; day: 16; **Record Level:** basisOfRecord: PreservedSpecimen
- **Type status:** Other material. **Occurrence:** catalogNumber: KPM-NI 23978; recordedBy: Yusuke Miyazaki; individualCount: 1; **Taxon:** scientificName: Pseudorasbora parva; **Location:** country: Japan; stateProvince: Iwate; locality: irrigation pond of the Kubo River Basin; verbatimLatitude: 38°55′25″N; verbatimLongitude: 141°04′06″E; **Identification:** identifiedBy: Yusuke Miyazaki; dateIdentified: 2009; **Event:** year: 2009; month: 5; day: 16; **Record Level:** basisOfRecord: PreservedSpecimen
- **Type status:** Other material. **Occurrence:** catalogNumber: KPM-NI 24413; recordedBy: Yusuke Miyazaki and Shogo Nishihara; individualCount: 1; **Taxon:** scientificName: Pseudorasbora parva; **Location:** country: Japan; stateProvince: Iwate; locality: channel of rice paddy, Tochikura River Basin; verbatimLatitude: 38°54′31″N; verbatimLongitude: 141°00′48″E; **Identification:** identifiedBy: Yusuke Miyazaki; dateIdentified: 2009; **Event:** year: 2009; month: 9; day: 21; **Record Level:** basisOfRecord: PreservedSpecimen
- **Type status:** Other material. **Occurrence:** catalogNumber: KPM-NI 24422; recordedBy: Yusuke Miyazaki; individualCount: 1; **Taxon:** scientificName: Pseudorasbora parva; **Location:** country: Japan; stateProvince: Iwate; municipality: Genbi Town; verbatimLatitude: 38°56′12″N; verbatimLongitude: 141°02′03″E; **Identification:** identifiedBy: Yusuke Miyazaki; dateIdentified: 2009; **Event:** year: 2009; month: 9; day: 20; **Record Level:** basisOfRecord: PreservedSpecimen
- **Type status:** Other material. **Occurrence:** catalogNumber: KPM-NI 24464; recordedBy: Yusuke Miyazaki; individualCount: 1; **Taxon:** scientificName: Pseudorasbora parva; **Location:** country: Japan; stateProvince: Iwate; municipality: Genbi Town; verbatimLatitude: 38°56′12″N; verbatimLongitude: 141°02′03″E; **Identification:** identifiedBy: Yusuke Miyazaki; dateIdentified: 2009; **Event:** year: 2009; month: 9; day: 21; **Record Level:** basisOfRecord: PreservedSpecimen
- **Type status:** Other material. **Occurrence:** catalogNumber: KPM-NI 24465; recordedBy: Yusuke Miyazaki; individualCount: 1; **Taxon:** scientificName: Pseudorasbora parva; **Location:** country: Japan; stateProvince: Iwate; municipality: Genbi Town; verbatimLatitude: 38°56′12″N; verbatimLongitude: 141°02′03″E; **Identification:** identifiedBy: Yusuke Miyazaki; dateIdentified: 2009; **Event:** year: 2009; month: 9; day: 21; **Record Level:** basisOfRecord: PreservedSpecimen
- **Type status:** Other material. **Occurrence:** catalogNumber: KPM-NI 24475; recordedBy: Yusuke Miyazaki; individualCount: 1; **Taxon:** scientificName: Pseudorasbora parva; **Location:** country: Japan; stateProvince: Iwate; municipality: Magaribuchi, Hagishou; verbatimLatitude: 38°55′49″N; verbatimLongitude: 141°01′56″E; **Identification:** identifiedBy: Yusuke Miyazaki; dateIdentified: 2009; **Event:** year: 2009; month: 9; day: 22; **Record Level:** basisOfRecord: PreservedSpecimen
- **Type status:** Other material. **Occurrence:** catalogNumber: KPM-NI 24476; recordedBy: Yusuke Miyazaki; individualCount: 1; **Taxon:** scientificName: Pseudorasbora parva; **Location:** country: Japan; stateProvince: Iwate; municipality: Magaribuchi, Hagishou; verbatimLatitude: 38°55′49″N; verbatimLongitude: 141°01′56″E; **Identification:** identifiedBy: Yusuke Miyazaki; dateIdentified: 2009; **Event:** year: 2009; month: 9; day: 22; **Record Level:** basisOfRecord: PreservedSpecimen
- **Type status:** Other material. **Occurrence:** catalogNumber: NSMT-P 90711; recordedBy: Y. Miyazaki; individualCount: 6; **Taxon:** scientificName: Pseudorasbora parva; **Location:** country: Japan; stateProvince: Iwate; municipality: Hagishou; verbatimLatitude: 38°55′54″N; verbatimLongitude: 141°02′01″E; **Identification:** identifiedBy: Yusuke Miyazaki; dateIdentified: 2008; **Event:** year: 2008; month: 5; day: 1; **Record Level:** basisOfRecord: PreservedSpecimen
- **Type status:** Other material. **Occurrence:** catalogNumber: NSMT-P 90720; recordedBy: Y. Miyazaki; individualCount: 9; **Taxon:** scientificName: Pseudorasbora parva; **Location:** country: Japan; stateProvince: Iwate; municipality: Hagishou; verbatimLatitude: 38°55′31″N; verbatimLongitude: 140°59′57″E; **Identification:** identifiedBy: Yusuke Miyazaki; dateIdentified: 2008; **Event:** year: 2008; month: 5; day: 3; **Record Level:** basisOfRecord: PreservedSpecimen
- **Type status:** Other material. **Occurrence:** catalogNumber: NSMT-P 90722; recordedBy: Y. Miyazaki; individualCount: 9; **Taxon:** scientificName: Pseudorasbora parva; **Location:** country: Japan; stateProvince: Iwate; municipality: Hagishou; verbatimLatitude: 38°54′32″N; verbatimLongitude: 141°00′45″E; **Identification:** identifiedBy: Yusuke Miyazaki; dateIdentified: 2008; **Event:** year: 2008; month: 5; day: 3; **Record Level:** basisOfRecord: PreservedSpecimen
- **Type status:** Other material. **Occurrence:** catalogNumber: NSMT-P 91202; recordedBy: Y. Miyazaki; individualCount: 1; **Taxon:** scientificName: Pseudorasbora parva; **Location:** country: Japan; stateProvince: Iwate; locality: Tochikura River; **Identification:** identifiedBy: Yusuke Miyazaki; dateIdentified: 2008; **Event:** year: 2008; month: 7; day: 5; **Record Level:** basisOfRecord: PreservedSpecimen
- **Type status:** Other material. **Occurrence:** catalogNumber: NSMT-P 91205; recordedBy: Y. Miyazaki; individualCount: 2; **Taxon:** scientificName: Pseudorasbora parva; **Location:** country: Japan; stateProvince: Iwate; locality: Tochikura River; **Identification:** identifiedBy: Yusuke Miyazaki; dateIdentified: 2008; **Event:** year: 2008; month: 7; day: 8; **Record Level:** basisOfRecord: PreservedSpecimen
- **Type status:** Other material. **Occurrence:** catalogNumber: NSMT-P 91651; recordedBy: Y. Miyazaki; individualCount: 1; **Taxon:** scientificName: Pseudorasbora parva; **Location:** country: Japan; stateProvince: Iwate; locality: Kubo River; **Identification:** identifiedBy: Yusuke Miyazaki; dateIdentified: 2008; **Event:** year: 2008; month: 7; day: 9; **Record Level:** basisOfRecord: PreservedSpecimen
- **Type status:** Other material. **Occurrence:** catalogNumber: NSMT-P 96831; recordedBy: Y. Miyazaki and Yoko Takata; individualCount: 1; **Taxon:** scientificName: Pseudorasbora parva; **Location:** country: Japan; stateProvince: Iwate; locality: Tochikura River; **Identification:** identifiedBy: Takashi P. Satoh; dateIdentified: 2010; **Event:** year: 2008; month: 10; day: 9; **Record Level:** basisOfRecord: PreservedSpecimen
- **Type status:** Other material. **Occurrence:** catalogNumber: NSMT-P 96856; recordedBy: Y. Miyazaki and Yoko Takata; individualCount: 3; **Taxon:** scientificName: Pseudorasbora parva; **Location:** country: Japan; stateProvince: Iwate; locality: irrigation pond of the Kubo River Basin; **Identification:** identifiedBy: Yoko Takata; dateIdentified: 2010; **Event:** year: 2008; month: 10; day: 8; **Record Level:** basisOfRecord: PreservedSpecimen
- **Type status:** Other material. **Occurrence:** catalogNumber: NSMT-P 96857; recordedBy: Y. Miyazaki and Yoko Takata; individualCount: 2; **Taxon:** scientificName: Pseudorasbora parva; **Location:** country: Japan; stateProvince: Iwate; municipality: Hagishou; **Identification:** identifiedBy: Takashi P. Satoh; dateIdentified: 2010; **Event:** year: 2008; month: 10; day: 8; **Record Level:** basisOfRecord: PreservedSpecimen
- **Type status:** Other material. **Occurrence:** catalogNumber: NSMT-P 96897; recordedBy: Y. Miyazaki and Yoko Takata; individualCount: 4; **Taxon:** scientificName: Pseudorasbora parva; **Location:** country: Japan; stateProvince: Iwate; locality: Tochikura River; **Identification:** identifiedBy: Yoko Takata; dateIdentified: 2010; **Event:** year: 2008; month: 10; day: 10; **Record Level:** basisOfRecord: PreservedSpecimen
- **Type status:** Other material. **Occurrence:** catalogNumber: NSMT-P 96899; recordedBy: Y. Miyazaki and Yoko Takata; individualCount: 2; **Taxon:** scientificName: Pseudorasbora parva; **Location:** country: Japan; stateProvince: Iwate; locality: Kubo River; **Identification:** identifiedBy: Yoko Takata; dateIdentified: 2010; **Event:** year: 2008; month: 10; day: 10; **Record Level:** basisOfRecord: PreservedSpecimen

##### Ecological interactions

###### Native status

Non-native (domestic non-native species in Iwate Prefecture: Matsuzawa and Senou 2008).

##### Distribution

Far East Asia

##### Notes

This species was captured only from lentic enviroments, and was often found together with *Cyprinus rubrofuscus*.

#### Pseudorasbora pumila pumila

Miyadi, 1930

##### Materials

- **Type status:** Other material. **Occurrence:** catalogNumber: KPM-NI 19447; recordedBy: Yusuke Miyazaki; individualCount: 1; **Taxon:** scientificName: Pseudorasbora pumila; **Location:** country: Japan; stateProvince: Iwate; locality: irrigation pond of the Kubo River Basin; **Identification:** identifiedBy: Hiroshi Senou; dateIdentified: 2007; **Event:** year: 2007; month: 9; day: 12; **Record Level:** basisOfRecord: PreservedSpecimen
- **Type status:** Other material. **Occurrence:** catalogNumber: KPM-NI 19448; recordedBy: Yusuke Miyazaki; individualCount: 1; **Taxon:** scientificName: Pseudorasbora pumila; **Location:** country: Japan; stateProvince: Iwate; locality: irrigation pond of the Kubo River Basin; **Identification:** identifiedBy: Hiroshi Senou; dateIdentified: 2007; **Event:** year: 2007; month: 9; day: 12; **Record Level:** basisOfRecord: PreservedSpecimen
- **Type status:** Other material. **Occurrence:** catalogNumber: KPM-NI 19449; recordedBy: Yusuke Miyazaki; individualCount: 1; **Taxon:** scientificName: Pseudorasbora pumila; **Location:** country: Japan; stateProvince: Iwate; locality: irrigation pond of the Kubo River Basin; **Identification:** identifiedBy: Hiroshi Senou; dateIdentified: 2007; **Event:** year: 2007; month: 9; day: 12; **Record Level:** basisOfRecord: PreservedSpecimen
- **Type status:** Other material. **Occurrence:** catalogNumber: KPM-NI 21219; recordedBy: Hiroshi Senou and Takumi Senou; individualCount: 3; **Taxon:** scientificName: Pseudorasbora pumila; **Location:** country: Japan; stateProvince: Iwate; locality: irrigation pond of the Kubo River Basin; **Identification:** identifiedBy: Hiroshi Senou; dateIdentified: 2008; **Event:** year: 2008; month: 5; day: 2; **Record Level:** basisOfRecord: PreservedSpecimen
- **Type status:** Other material. **Occurrence:** catalogNumber: KPM-NI 21227; recordedBy: Yusuke Miyazaki and Hiroshi Senou; individualCount: 2; **Taxon:** scientificName: Pseudorasbora pumila; **Location:** country: Japan; stateProvince: Iwate; locality: irrigation pond of the Kubo River Basin; **Identification:** identifiedBy: Yusuke Miyazaki; dateIdentified: 2008; **Event:** year: 2008; month: 5; day: 3; **Record Level:** basisOfRecord: PreservedSpecimen
- **Type status:** Other material. **Occurrence:** catalogNumber: KPM-NI 21403; recordedBy: Yusuke Miyazaki; individualCount: 1; **Taxon:** scientificName: Pseudorasbora pumila; **Location:** country: Japan; stateProvince: Iwate; locality: irrigation pond of the Kubo River Basin; **Identification:** identifiedBy: Hiroshi Senou; dateIdentified: 2008; **Event:** year: 2008; month: 6; day: 8; **Record Level:** basisOfRecord: PreservedSpecimen
- **Type status:** Other material. **Occurrence:** catalogNumber: KPM-NI 21405; recordedBy: Yusuke Miyazaki; individualCount: 1; **Taxon:** scientificName: Pseudorasbora pumila; **Location:** country: Japan; stateProvince: Iwate; locality: irrigation pond of the Kubo River Basin; **Identification:** identifiedBy: Hiroshi Senou; dateIdentified: 2008; **Event:** year: 2008; month: 6; day: 8; **Record Level:** basisOfRecord: PreservedSpecimen
- **Type status:** Other material. **Occurrence:** catalogNumber: KPM-NI 22264; recordedBy: Yusuke Miyazaki; individualCount: 1; **Taxon:** scientificName: Pseudorasbora pumila; **Location:** country: Japan; stateProvince: Iwate; locality: irrigation pond of the Kubo River Basin; **Identification:** identifiedBy: Yusuke Miyazaki; dateIdentified: 2008; **Event:** year: 2008; month: 8; day: 25; **Record Level:** basisOfRecord: PreservedSpecimen
- **Type status:** Other material. **Occurrence:** catalogNumber: KPM-NI 22265; recordedBy: Yusuke Miyazaki; individualCount: 1; **Taxon:** scientificName: Pseudorasbora pumila; **Location:** country: Japan; stateProvince: Iwate; locality: irrigation pond of the Kubo River Basin; **Identification:** identifiedBy: Yusuke Miyazaki; dateIdentified: 2008; **Event:** year: 2008; month: 8; day: 25; **Record Level:** basisOfRecord: PreservedSpecimen
- **Type status:** Other material. **Occurrence:** catalogNumber: KPM-NI 22266; recordedBy: Yusuke Miyazaki; individualCount: 1; **Taxon:** scientificName: Pseudorasbora pumila; **Location:** country: Japan; stateProvince: Iwate; locality: irrigation pond of the Kubo River Basin; **Identification:** identifiedBy: Yusuke Miyazaki; dateIdentified: 2008; **Event:** year: 2008; month: 8; day: 26; **Record Level:** basisOfRecord: PreservedSpecimen
- **Type status:** Other material. **Occurrence:** catalogNumber: KPM-NI 22269; recordedBy: Yusuke Miyazaki; individualCount: 1; **Taxon:** scientificName: Pseudorasbora pumila; **Location:** country: Japan; stateProvince: Iwate; locality: irrigation pond of the Kubo River Basin; **Identification:** identifiedBy: Yusuke Miyazaki; dateIdentified: 2008; **Event:** year: 2008; month: 8; day: 26; **Record Level:** basisOfRecord: PreservedSpecimen
- **Type status:** Other material. **Occurrence:** catalogNumber: KPM-NI 22280; recordedBy: Yusuke Miyazaki; individualCount: 1; **Taxon:** scientificName: Pseudorasbora pumila; **Location:** country: Japan; stateProvince: Iwate; locality: irrigation pond of the Kubo River Basin; **Identification:** identifiedBy: Yusuke Miyazaki; dateIdentified: 2008; **Event:** year: 2008; month: 8; day: 29; **Record Level:** basisOfRecord: PreservedSpecimen
- **Type status:** Other material. **Occurrence:** catalogNumber: KPM-NI 22281; recordedBy: Yusuke Miyazaki; individualCount: 1; **Taxon:** scientificName: Pseudorasbora pumila; **Location:** country: Japan; stateProvince: Iwate; locality: irrigation pond of the Kubo River Basin; **Identification:** identifiedBy: Yusuke Miyazaki; dateIdentified: 2008; **Event:** year: 2008; month: 8; day: 27; **Record Level:** basisOfRecord: PreservedSpecimen
- **Type status:** Other material. **Occurrence:** catalogNumber: KPM-NI 22313; recordedBy: Yusuke Miyazaki; individualCount: 1; **Taxon:** scientificName: Pseudorasbora pumila; **Location:** country: Japan; stateProvince: Iwate; locality: irrigation pond of the Kubo River Basin; **Identification:** identifiedBy: Yusuke Miyazaki; dateIdentified: 2008; **Event:** year: 2008; month: 8; day: 28; **Record Level:** basisOfRecord: PreservedSpecimen
- **Type status:** Other material. **Occurrence:** catalogNumber: KPM-NI 23964; recordedBy: Yusuke Miyazaki; individualCount: 1; **Taxon:** scientificName: Pseudorasbora pumila; **Location:** country: Japan; stateProvince: Iwate; locality: irrigation pond of the Kubo River Basin; **Identification:** identifiedBy: Yusuke Miyazaki; dateIdentified: 2009; **Event:** year: 2009; month: 5; day: 3; **Record Level:** basisOfRecord: PreservedSpecimen
- **Type status:** Other material. **Occurrence:** catalogNumber: KPM-NI 23965; recordedBy: Yusuke Miyazaki; individualCount: 1; **Taxon:** scientificName: Pseudorasbora pumila; **Location:** country: Japan; stateProvince: Iwate; locality: irrigation pond of the Kubo River Basin; **Identification:** identifiedBy: Yusuke Miyazaki; dateIdentified: 2009; **Event:** year: 2009; month: 5; day: 3; **Record Level:** basisOfRecord: PreservedSpecimen
- **Type status:** Other material. **Occurrence:** catalogNumber: KPM-NI 23966; recordedBy: Yusuke Miyazaki; individualCount: 1; **Taxon:** scientificName: Pseudorasbora pumila; **Location:** country: Japan; stateProvince: Iwate; locality: irrigation pond of the Kubo River Basin; **Identification:** identifiedBy: Yusuke Miyazaki; dateIdentified: 2009; **Event:** year: 2009; month: 5; day: 19; **Record Level:** basisOfRecord: PreservedSpecimen
- **Type status:** Other material. **Occurrence:** catalogNumber: KPM-NI 23967; recordedBy: Yusuke Miyazaki; individualCount: 1; **Taxon:** scientificName: Pseudorasbora pumila; **Location:** country: Japan; stateProvince: Iwate; **Identification:** identifiedBy: Yusuke Miyazaki; dateIdentified: 2009; **Event:** year: 2009; month: 5; day: 2; **Record Level:** basisOfRecord: PreservedSpecimen
- **Type status:** Other material. **Occurrence:** catalogNumber: KPM-NI 23968; recordedBy: Yusuke Miyazaki; individualCount: 1; **Taxon:** scientificName: Pseudorasbora pumila; **Location:** country: Japan; stateProvince: Iwate; municipality: Dounosawa, Hagishou; **Identification:** identifiedBy: Yusuke Miyazaki; dateIdentified: 2009; **Event:** year: 2009; month: 7; day: 13; **Record Level:** basisOfRecord: PreservedSpecimen
- **Type status:** Other material. **Occurrence:** catalogNumber: KPM-NI 23969; recordedBy: Yusuke Miyazaki; individualCount: 1; **Taxon:** scientificName: Pseudorasbora pumila; **Location:** country: Japan; stateProvince: Iwate; locality: irrigation pond of the Kubo River Basin; **Identification:** identifiedBy: Yusuke Miyazaki; dateIdentified: 2009; **Event:** year: 2009; month: 5; day: 3; **Record Level:** basisOfRecord: PreservedSpecimen
- **Type status:** Other material. **Occurrence:** catalogNumber: KPM-NI 23970; recordedBy: Yusuke Miyazaki; individualCount: 1; **Taxon:** scientificName: Pseudorasbora pumila; **Location:** country: Japan; stateProvince: Iwate; locality: irrigation pond of the Kubo River Basin; **Identification:** identifiedBy: Yusuke Miyazaki; dateIdentified: 2009; **Event:** year: 2009; month: 5; day: 3; **Record Level:** basisOfRecord: PreservedSpecimen
- **Type status:** Other material. **Occurrence:** catalogNumber: KPM-NI 24993; recordedBy: Yusuke Miyazaki; individualCount: 1; **Taxon:** scientificName: Pseudorasbora pumila; **Location:** country: Japan; stateProvince: Iwate; locality: irrigation pond of the Kubo River Basin; **Identification:** identifiedBy: Yusuke Miyazaki; dateIdentified: 2009; **Event:** year: 2008; month: 10; day: 9; **Record Level:** basisOfRecord: PreservedSpecimen
- **Type status:** Other material. **Occurrence:** catalogNumber: KPM-NI 31018; recordedBy: Yusuke Miyazaki; individualCount: 1; **Taxon:** scientificName: Pseudorasbora pumila; **Location:** country: Japan; stateProvince: Iwate; locality: irrigation pond of the Kubo River Basin; **Identification:** identifiedBy: Yusuke Miyazaki; dateIdentified: 2012; **Event:** year: 2010; month: 9; day: 4; **Record Level:** basisOfRecord: PreservedSpecimen
- **Type status:** Other material. **Occurrence:** catalogNumber: KPM-NI 31715; recordedBy: Yusuke Miyazaki; individualCount: 1; **Taxon:** scientificName: Pseudorasbora pumila; **Location:** country: Japan; stateProvince: Iwate; municipality: Dounosawa, Hagishou; **Identification:** identifiedBy: Yusuke Miyazaki; dateIdentified: 2009; **Event:** year: 2009; month: 9; day: 22; **Record Level:** basisOfRecord: PreservedSpecimen
- **Type status:** Other material. **Occurrence:** catalogNumber: KPM-NI 31718; recordedBy: Yusuke Miyazaki; individualCount: 1; **Taxon:** scientificName: Pseudorasbora pumila; **Location:** country: Japan; stateProvince: Iwate; municipality: Dounosawa, Hagishou; **Identification:** identifiedBy: Yusuke Miyazaki; dateIdentified: 2009; **Event:** year: 2009; month: 9; day: 20; **Record Level:** basisOfRecord: PreservedSpecimen
- **Type status:** Other material. **Occurrence:** catalogNumber: KPM-NI 31719; recordedBy: Yusuke Miyazaki; individualCount: 1; **Taxon:** scientificName: Pseudorasbora pumila; **Location:** country: Japan; stateProvince: Iwate; municipality: Dounosawa, Hagishou; **Identification:** identifiedBy: Yusuke Miyazaki; dateIdentified: 2009; **Event:** year: 2009; month: 9; day: 20; **Record Level:** basisOfRecord: PreservedSpecimen
- **Type status:** Other material. **Occurrence:** catalogNumber: KPM-NI 31720; recordedBy: Yusuke Miyazaki; individualCount: 1; **Taxon:** scientificName: Pseudorasbora pumila; **Location:** country: Japan; stateProvince: Iwate; municipality: Dounosawa, Hagishou; **Identification:** identifiedBy: Yusuke Miyazaki; dateIdentified: 2009; **Event:** year: 2009; month: 9; day: 20; **Record Level:** basisOfRecord: PreservedSpecimen
- **Type status:** Other material. **Occurrence:** catalogNumber: KPM-NI 31721; recordedBy: Yusuke Miyazaki; individualCount: 1; **Taxon:** scientificName: Pseudorasbora pumila; **Location:** country: Japan; stateProvince: Iwate; municipality: Dounosawa, Hagishou; **Identification:** identifiedBy: Yusuke Miyazaki; dateIdentified: 2009; **Event:** year: 2009; month: 9; day: 20; **Record Level:** basisOfRecord: PreservedSpecimen
- **Type status:** Other material. **Occurrence:** catalogNumber: KPM-NI 31716; recordedBy: Yusuke Miyazaki; individualCount: 1; **Taxon:** scientificName: Pseudorasbora pumila; **Location:** country: Japan; stateProvince: Iwate; locality: irrigation pond of the Kubo River Basin; **Identification:** identifiedBy: Yusuke Miyazaki; dateIdentified: 2008; **Event:** year: 2008; month: 8; day: 29; **Record Level:** basisOfRecord: PreservedSpecimen
- **Type status:** Other material. **Occurrence:** catalogNumber: KPM-NI 31717; recordedBy: Yusuke Miyazaki; individualCount: 1; **Taxon:** scientificName: Pseudorasbora pumila; **Location:** country: Japan; stateProvince: Iwate; locality: irrigation pond of the Kubo River Basin; **Identification:** identifiedBy: Yusuke Miyazaki; dateIdentified: 2008; **Event:** year: 2008; month: 8; day: 29; **Record Level:** basisOfRecord: PreservedSpecimen
- **Type status:** Other material. **Occurrence:** catalogNumber: KPM-NI 31722; recordedBy: Yusuke Miyazaki; individualCount: 1; **Taxon:** scientificName: Pseudorasbora pumila; **Location:** country: Japan; stateProvince: Iwate; locality: irrigation pond of the Kubo River Basin; **Identification:** identifiedBy: Yusuke Miyazaki; dateIdentified: 2008; **Event:** year: 2008; month: 8; day: 27; **Record Level:** basisOfRecord: PreservedSpecimen
- **Type status:** Other material. **Occurrence:** catalogNumber: KPM-NI 31723; recordedBy: Yusuke Miyazaki; individualCount: 1; **Taxon:** scientificName: Pseudorasbora pumila; **Location:** country: Japan; stateProvince: Iwate; locality: irrigation pond of the Kubo River Basin; **Identification:** identifiedBy: Yusuke Miyazaki; dateIdentified: 2008; **Event:** year: 2008; month: 8; day: 27; **Record Level:** basisOfRecord: PreservedSpecimen
- **Type status:** Other material. **Occurrence:** catalogNumber: KPM-NI 31724; recordedBy: Yusuke Miyazaki; individualCount: 1; **Taxon:** scientificName: Pseudorasbora pumila; **Location:** country: Japan; stateProvince: Iwate; locality: irrigation pond of the Kubo River Basin; **Identification:** identifiedBy: Yusuke Miyazaki; dateIdentified: 2010; **Event:** year: 2010; month: 9; day: 5; **Record Level:** basisOfRecord: PreservedSpecimen
- **Type status:** Other material. **Occurrence:** catalogNumber: KPM-NI 31725; recordedBy: Yusuke Miyazaki; individualCount: 1; **Taxon:** scientificName: Pseudorasbora pumila; **Location:** country: Japan; stateProvince: Iwate; locality: irrigation pond of the Kubo River Basin; **Identification:** identifiedBy: Yusuke Miyazaki; dateIdentified: 2010; **Event:** year: 2010; month: 9; day: 5; **Record Level:** basisOfRecord: PreservedSpecimen
- **Type status:** Other material. **Occurrence:** catalogNumber: KPM-NI 31726; recordedBy: Yusuke Miyazaki; individualCount: 1; **Taxon:** scientificName: Pseudorasbora pumila; **Location:** country: Japan; stateProvince: Iwate; locality: irrigation pond of the Kubo River Basin; **Identification:** identifiedBy: Yusuke Miyazaki; dateIdentified: 2010; **Event:** year: 2010; month: 9; day: 4; **Record Level:** basisOfRecord: PreservedSpecimen
- **Type status:** Other material. **Occurrence:** catalogNumber: KPM-NI 31727; recordedBy: Yusuke Miyazaki; individualCount: 1; **Taxon:** scientificName: Pseudorasbora pumila; **Location:** country: Japan; stateProvince: Iwate; locality: irrigation pond of the Kubo River Basin; **Identification:** identifiedBy: Yusuke Miyazaki; dateIdentified: 2010; **Event:** year: 2010; month: 9; day: 4; **Record Level:** basisOfRecord: PreservedSpecimen
- **Type status:** Other material. **Occurrence:** catalogNumber: KPM-NI 31728; recordedBy: Yusuke Miyazaki; individualCount: 1; **Taxon:** scientificName: Pseudorasbora pumila; **Location:** country: Japan; stateProvince: Iwate; locality: irrigation pond of the Kubo River Basin; **Identification:** identifiedBy: Yusuke Miyazaki; dateIdentified: 2010; **Event:** year: 2010; month: 9; day: 4; **Record Level:** basisOfRecord: PreservedSpecimen
- **Type status:** Other material. **Occurrence:** catalogNumber: KPM-NI 31729; recordedBy: Yusuke Miyazaki; individualCount: 1; **Taxon:** scientificName: Pseudorasbora pumila; **Location:** country: Japan; stateProvince: Iwate; locality: irrigation pond of the Kubo River Basin; **Identification:** identifiedBy: Yusuke Miyazaki; dateIdentified: 2010; **Event:** year: 2010; month: 9; day: 4; **Record Level:** basisOfRecord: PreservedSpecimen
- **Type status:** Other material. **Occurrence:** catalogNumber: KPM-NI 31730; recordedBy: Yusuke Miyazaki; individualCount: 1; **Taxon:** scientificName: Pseudorasbora pumila; **Location:** country: Japan; stateProvince: Iwate; locality: irrigation pond of the Kubo River Basin; **Identification:** identifiedBy: Yusuke Miyazaki; dateIdentified: 2010; **Event:** year: 2010; month: 9; day: 4; **Record Level:** basisOfRecord: PreservedSpecimen
- **Type status:** Other material. **Occurrence:** catalogNumber: KPM-NI 31731; recordedBy: Yusuke Miyazaki; individualCount: 1; **Taxon:** scientificName: Pseudorasbora pumila; **Location:** country: Japan; stateProvince: Iwate; locality: irrigation pond of the Kubo River Basin; **Identification:** identifiedBy: Yusuke Miyazaki; dateIdentified: 2010; **Event:** year: 2010; month: 9; day: 4; **Record Level:** basisOfRecord: PreservedSpecimen
- **Type status:** Other material. **Occurrence:** catalogNumber: KPM-NI 31732; recordedBy: Yusuke Miyazaki; individualCount: 1; **Taxon:** scientificName: Pseudorasbora pumila; **Location:** country: Japan; stateProvince: Iwate; locality: irrigation pond of the Kubo River Basin; **Identification:** identifiedBy: Yusuke Miyazaki; dateIdentified: 2010; **Event:** year: 2010; month: 9; day: 4; **Record Level:** basisOfRecord: PreservedSpecimen
- **Type status:** Other material. **Occurrence:** catalogNumber: KPM-NI 31733; recordedBy: Yusuke Miyazaki; individualCount: 1; **Taxon:** scientificName: Pseudorasbora pumila; **Location:** country: Japan; stateProvince: Iwate; locality: irrigation pond of the Kubo River Basin; **Identification:** identifiedBy: Yusuke Miyazaki; dateIdentified: 2010; **Event:** year: 2010; month: 9; day: 4; **Record Level:** basisOfRecord: PreservedSpecimen
- **Type status:** Other material. **Occurrence:** catalogNumber: KPM-NI 31734; recordedBy: Yusuke Miyazaki; individualCount: 1; **Taxon:** scientificName: Pseudorasbora pumila; **Location:** country: Japan; stateProvince: Iwate; locality: irrigation pond of the Kubo River Basin; **Identification:** identifiedBy: Yusuke Miyazaki; dateIdentified: 2010; **Event:** year: 2010; month: 9; day: 4; **Record Level:** basisOfRecord: PreservedSpecimen
- **Type status:** Other material. **Occurrence:** catalogNumber: KPM-NI 31735; recordedBy: Yusuke Miyazaki; individualCount: 1; **Taxon:** scientificName: Pseudorasbora pumila; **Location:** country: Japan; stateProvince: Iwate; locality: irrigation pond of the Kubo River Basin; **Identification:** identifiedBy: Yusuke Miyazaki; dateIdentified: 2010; **Event:** year: 2010; month: 9; day: 4; **Record Level:** basisOfRecord: PreservedSpecimen
- **Type status:** Other material. **Occurrence:** catalogNumber: KPM-NI 31736; recordedBy: Yusuke Miyazaki; individualCount: 1; **Taxon:** scientificName: Pseudorasbora pumila; **Location:** country: Japan; stateProvince: Iwate; locality: irrigation pond of the Kubo River Basin; **Identification:** identifiedBy: Yusuke Miyazaki; dateIdentified: 2010; **Event:** year: 2010; month: 9; day: 4; **Record Level:** basisOfRecord: PreservedSpecimen
- **Type status:** Other material. **Occurrence:** catalogNumber: KPM-NI 31737; recordedBy: Yusuke Miyazaki; individualCount: 1; **Taxon:** scientificName: Pseudorasbora pumila; **Location:** country: Japan; stateProvince: Iwate; locality: irrigation pond of the Kubo River Basin; **Identification:** identifiedBy: Yusuke Miyazaki; dateIdentified: 2010; **Event:** year: 2010; month: 9; day: 4; **Record Level:** basisOfRecord: PreservedSpecimen
- **Type status:** Other material. **Occurrence:** catalogNumber: KPM-NI 31738; recordedBy: Yusuke Miyazaki; individualCount: 1; **Taxon:** scientificName: Pseudorasbora pumila; **Location:** country: Japan; stateProvince: Iwate; locality: irrigation pond of the Kubo River Basin; **Identification:** identifiedBy: Yusuke Miyazaki; dateIdentified: 2010; **Event:** year: 2010; month: 9; day: 4; **Record Level:** basisOfRecord: PreservedSpecimen
- **Type status:** Other material. **Occurrence:** catalogNumber: KPM-NI 31739; recordedBy: Yusuke Miyazaki; individualCount: 1; **Taxon:** scientificName: Pseudorasbora pumila; **Location:** country: Japan; stateProvince: Iwate; locality: irrigation pond of the Kubo River Basin; **Identification:** identifiedBy: Yusuke Miyazaki; dateIdentified: 2010; **Event:** year: 2010; month: 9; day: 4; **Record Level:** basisOfRecord: PreservedSpecimen
- **Type status:** Other material. **Occurrence:** catalogNumber: KPM-NI 31740; recordedBy: Yusuke Miyazaki; individualCount: 1; **Taxon:** scientificName: Pseudorasbora pumila; **Location:** country: Japan; stateProvince: Iwate; locality: irrigation pond of the Kubo River Basin; **Identification:** identifiedBy: Yusuke Miyazaki; dateIdentified: 2010; **Event:** year: 2010; month: 9; day: 4; **Record Level:** basisOfRecord: PreservedSpecimen
- **Type status:** Other material. **Occurrence:** catalogNumber: KPM-NI 31741; recordedBy: Yusuke Miyazaki; individualCount: 1; **Taxon:** scientificName: Pseudorasbora pumila; **Location:** country: Japan; stateProvince: Iwate; locality: irrigation pond of the Kubo River Basin; **Identification:** identifiedBy: Yusuke Miyazaki; dateIdentified: 2010; **Event:** year: 2010; month: 9; day: 4; **Record Level:** basisOfRecord: PreservedSpecimen
- **Type status:** Other material. **Occurrence:** catalogNumber: KPM-NI 31742; recordedBy: Yusuke Miyazaki; individualCount: 1; **Taxon:** scientificName: Pseudorasbora pumila; **Location:** country: Japan; stateProvince: Iwate; locality: irrigation pond of the Kubo River Basin; **Identification:** identifiedBy: Yusuke Miyazaki; dateIdentified: 2010; **Event:** year: 2010; month: 9; day: 4; **Record Level:** basisOfRecord: PreservedSpecimen
- **Type status:** Other material. **Occurrence:** catalogNumber: KPM-NI 31744; recordedBy: Yusuke Miyazaki; individualCount: 1; **Taxon:** scientificName: Pseudorasbora pumila; **Location:** country: Japan; stateProvince: Iwate; locality: irrigation pond of the Kubo River Basin; **Identification:** identifiedBy: Yusuke Miyazaki; dateIdentified: 2010; **Event:** year: 2010; month: 9; day: 5; **Record Level:** basisOfRecord: PreservedSpecimen
- **Type status:** Other material. **Occurrence:** catalogNumber: KPM-NI 31745; recordedBy: Yusuke Miyazaki; individualCount: 1; **Taxon:** scientificName: Pseudorasbora pumila; **Location:** country: Japan; stateProvince: Iwate; locality: irrigation pond of the Kubo River Basin; **Identification:** identifiedBy: Yusuke Miyazaki; dateIdentified: 2010; **Event:** year: 2010; month: 9; day: 5; **Record Level:** basisOfRecord: PreservedSpecimen
- **Type status:** Other material. **Occurrence:** catalogNumber: KPM-NI 31746; recordedBy: Yusuke Miyazaki; individualCount: 1; **Taxon:** scientificName: Pseudorasbora pumila; **Location:** country: Japan; stateProvince: Iwate; locality: irrigation pond of the Kubo River Basin; **Identification:** identifiedBy: Yusuke Miyazaki; dateIdentified: 2010; **Event:** year: 2010; month: 9; day: 5; **Record Level:** basisOfRecord: PreservedSpecimen
- **Type status:** Other material. **Occurrence:** catalogNumber: KPM-NI 31747; recordedBy: Yusuke Miyazaki; individualCount: 1; **Taxon:** scientificName: Pseudorasbora pumila; **Location:** country: Japan; stateProvince: Iwate; locality: irrigation pond of the Kubo River Basin; **Identification:** identifiedBy: Yusuke Miyazaki; dateIdentified: 2010; **Event:** year: 2010; month: 9; day: 5; **Record Level:** basisOfRecord: PreservedSpecimen
- **Type status:** Other material. **Occurrence:** catalogNumber: KPM-NI 31748; recordedBy: Yusuke Miyazaki; individualCount: 1; **Taxon:** scientificName: Pseudorasbora pumila; **Location:** country: Japan; stateProvince: Iwate; locality: irrigation pond of the Kubo River Basin; **Identification:** identifiedBy: Yusuke Miyazaki; dateIdentified: 2010; **Event:** year: 2010; month: 9; day: 5; **Record Level:** basisOfRecord: PreservedSpecimen
- **Type status:** Other material. **Occurrence:** catalogNumber: KPM-NI 31749; recordedBy: Yusuke Miyazaki; individualCount: 1; **Taxon:** scientificName: Pseudorasbora pumila; **Location:** country: Japan; stateProvince: Iwate; locality: irrigation pond of the Kubo River Basin; **Identification:** identifiedBy: Yusuke Miyazaki; dateIdentified: 2010; **Event:** year: 2010; month: 9; day: 5; **Record Level:** basisOfRecord: PreservedSpecimen
- **Type status:** Other material. **Occurrence:** catalogNumber: KPM-NI 31750; recordedBy: Yusuke Miyazaki; individualCount: 1; **Taxon:** scientificName: Pseudorasbora pumila; **Location:** country: Japan; stateProvince: Iwate; locality: irrigation pond of the Kubo River Basin; **Identification:** identifiedBy: Yusuke Miyazaki; dateIdentified: 2010; **Event:** year: 2010; month: 9; day: 5; **Record Level:** basisOfRecord: PreservedSpecimen
- **Type status:** Other material. **Occurrence:** catalogNumber: KPM-NI 31751; recordedBy: Yusuke Miyazaki; individualCount: 1; **Taxon:** scientificName: Pseudorasbora pumila; **Location:** country: Japan; stateProvince: Iwate; locality: irrigation pond of the Kubo River Basin; **Identification:** identifiedBy: Yusuke Miyazaki; dateIdentified: 2010; **Event:** year: 2010; month: 9; day: 5; **Record Level:** basisOfRecord: PreservedSpecimen
- **Type status:** Other material. **Occurrence:** catalogNumber: KPM-NI 31752; recordedBy: Yusuke Miyazaki; individualCount: 1; **Taxon:** scientificName: Pseudorasbora pumila; **Location:** country: Japan; stateProvince: Iwate; locality: irrigation pond of the Kubo River Basin; **Identification:** identifiedBy: Yusuke Miyazaki; dateIdentified: 2010; **Event:** year: 2010; month: 9; day: 5; **Record Level:** basisOfRecord: PreservedSpecimen
- **Type status:** Other material. **Occurrence:** catalogNumber: KPM-NI 31753; recordedBy: Yusuke Miyazaki; individualCount: 1; **Taxon:** scientificName: Pseudorasbora pumila; **Location:** country: Japan; stateProvince: Iwate; locality: irrigation pond of the Kubo River Basin; **Identification:** identifiedBy: Yusuke Miyazaki; dateIdentified: 2010; **Event:** year: 2010; month: 9; day: 5; **Record Level:** basisOfRecord: PreservedSpecimen
- **Type status:** Other material. **Occurrence:** catalogNumber: KPM-NI 31754; recordedBy: Yusuke Miyazaki; individualCount: 1; **Taxon:** scientificName: Pseudorasbora pumila; **Location:** country: Japan; stateProvince: Iwate; locality: irrigation pond of the Kubo River Basin; **Identification:** identifiedBy: Yusuke Miyazaki; dateIdentified: 2010; **Event:** year: 2010; month: 9; day: 5; **Record Level:** basisOfRecord: PreservedSpecimen
- **Type status:** Other material. **Occurrence:** catalogNumber: KPM-NI 31755; recordedBy: Yusuke Miyazaki; individualCount: 1; **Taxon:** scientificName: Pseudorasbora pumila; **Location:** country: Japan; stateProvince: Iwate; locality: irrigation pond of the Kubo River Basin; **Identification:** identifiedBy: Yusuke Miyazaki; dateIdentified: 2010; **Event:** year: 2010; month: 9; day: 5; **Record Level:** basisOfRecord: PreservedSpecimen
- **Type status:** Other material. **Occurrence:** catalogNumber: KPM-NI 31756; recordedBy: Yusuke Miyazaki; individualCount: 1; **Taxon:** scientificName: Pseudorasbora pumila; **Location:** country: Japan; stateProvince: Iwate; locality: irrigation pond of the Kubo River Basin; **Identification:** identifiedBy: Yusuke Miyazaki; dateIdentified: 2010; **Event:** year: 2010; month: 9; day: 5; **Record Level:** basisOfRecord: PreservedSpecimen
- **Type status:** Other material. **Occurrence:** catalogNumber: KPM-NI 31757; recordedBy: Yusuke Miyazaki; individualCount: 1; **Taxon:** scientificName: Pseudorasbora pumila; **Location:** country: Japan; stateProvince: Iwate; locality: irrigation pond of the Kubo River Basin; **Identification:** identifiedBy: Yusuke Miyazaki; dateIdentified: 2010; **Event:** year: 2010; month: 9; day: 5; **Record Level:** basisOfRecord: PreservedSpecimen
- **Type status:** Other material. **Occurrence:** catalogNumber: KPM-NI 31758; recordedBy: Yusuke Miyazaki; individualCount: 1; **Taxon:** scientificName: Pseudorasbora pumila; **Location:** country: Japan; stateProvince: Iwate; locality: irrigation pond of the Kubo River Basin; **Identification:** identifiedBy: Yusuke Miyazaki; dateIdentified: 2010; **Event:** year: 2010; month: 9; day: 5; **Record Level:** basisOfRecord: PreservedSpecimen
- **Type status:** Other material. **Occurrence:** catalogNumber: KPM-NI 31759; recordedBy: Yusuke Miyazaki; individualCount: 1; **Taxon:** scientificName: Pseudorasbora pumila; **Location:** country: Japan; stateProvince: Iwate; locality: irrigation pond of the Kubo River Basin; **Identification:** identifiedBy: Yusuke Miyazaki; dateIdentified: 2010; **Event:** year: 2010; month: 9; day: 5; **Record Level:** basisOfRecord: PreservedSpecimen
- **Type status:** Other material. **Occurrence:** catalogNumber: KPM-NI 31760; recordedBy: Yusuke Miyazaki; individualCount: 1; **Taxon:** scientificName: Pseudorasbora pumila; **Location:** country: Japan; stateProvince: Iwate; locality: irrigation pond of the Kubo River Basin; **Identification:** identifiedBy: Yusuke Miyazaki; dateIdentified: 2010; **Event:** year: 2010; month: 9; day: 5; **Record Level:** basisOfRecord: PreservedSpecimen
- **Type status:** Other material. **Occurrence:** catalogNumber: KPM-NI 31761; recordedBy: Yusuke Miyazaki; individualCount: 1; **Taxon:** scientificName: Pseudorasbora pumila; **Location:** country: Japan; stateProvince: Iwate; locality: irrigation pond of the Kubo River Basin; **Identification:** identifiedBy: Yusuke Miyazaki; dateIdentified: 2010; **Event:** year: 2010; month: 9; day: 5; **Record Level:** basisOfRecord: PreservedSpecimen
- **Type status:** Other material. **Occurrence:** catalogNumber: KPM-NI 31762; recordedBy: Yusuke Miyazaki; individualCount: 1; **Taxon:** scientificName: Pseudorasbora pumila; **Location:** country: Japan; stateProvince: Iwate; locality: irrigation pond of the Kubo River Basin; **Identification:** identifiedBy: Yusuke Miyazaki; dateIdentified: 2010; **Event:** year: 2010; month: 9; day: 5; **Record Level:** basisOfRecord: PreservedSpecimen
- **Type status:** Other material. **Occurrence:** catalogNumber: KPM-NI 31763; recordedBy: Yusuke Miyazaki; individualCount: 1; **Taxon:** scientificName: Pseudorasbora pumila; **Location:** country: Japan; stateProvince: Iwate; locality: irrigation pond of the Kubo River Basin; **Identification:** identifiedBy: Yusuke Miyazaki; dateIdentified: 2010; **Event:** year: 2010; month: 9; day: 5; **Record Level:** basisOfRecord: PreservedSpecimen
- **Type status:** Other material. **Occurrence:** catalogNumber: KPM-NI 31764; recordedBy: Yusuke Miyazaki; individualCount: 1; **Taxon:** scientificName: Pseudorasbora pumila; **Location:** country: Japan; stateProvince: Iwate; locality: irrigation pond of the Kubo River Basin; **Identification:** identifiedBy: Yusuke Miyazaki; dateIdentified: 2010; **Event:** year: 2010; month: 9; day: 5; **Record Level:** basisOfRecord: PreservedSpecimen
- **Type status:** Other material. **Occurrence:** catalogNumber: KPM-NI 31765; recordedBy: Yusuke Miyazaki; individualCount: 1; **Taxon:** scientificName: Pseudorasbora pumila; **Location:** country: Japan; stateProvince: Iwate; locality: irrigation pond of the Kubo River Basin; **Identification:** identifiedBy: Yusuke Miyazaki; dateIdentified: 2010; **Event:** year: 2010; month: 9; day: 5; **Record Level:** basisOfRecord: PreservedSpecimen
- **Type status:** Other material. **Occurrence:** catalogNumber: KPM-NI 31766; recordedBy: Yusuke Miyazaki; individualCount: 1; **Taxon:** scientificName: Pseudorasbora pumila; **Location:** country: Japan; stateProvince: Iwate; locality: irrigation pond of the Kubo River Basin; **Identification:** identifiedBy: Yusuke Miyazaki; dateIdentified: 2010; **Event:** year: 2010; month: 9; day: 5; **Record Level:** basisOfRecord: PreservedSpecimen
- **Type status:** Other material. **Occurrence:** catalogNumber: KPM-NI 31767; recordedBy: Yusuke Miyazaki; individualCount: 1; **Taxon:** scientificName: Pseudorasbora pumila; **Location:** country: Japan; stateProvince: Iwate; locality: irrigation pond of the Kubo River Basin; **Identification:** identifiedBy: Yusuke Miyazaki; dateIdentified: 2010; **Event:** year: 2010; month: 9; day: 5; **Record Level:** basisOfRecord: PreservedSpecimen
- **Type status:** Other material. **Occurrence:** catalogNumber: KPM-NI 31768; recordedBy: Yusuke Miyazaki; individualCount: 1; **Taxon:** scientificName: Pseudorasbora pumila; **Location:** country: Japan; stateProvince: Iwate; locality: irrigation pond of the Kubo River Basin; **Identification:** identifiedBy: Yusuke Miyazaki; dateIdentified: 2010; **Event:** year: 2010; month: 9; day: 5; **Record Level:** basisOfRecord: PreservedSpecimen
- **Type status:** Other material. **Occurrence:** catalogNumber: KPM-NI 31769; recordedBy: Yusuke Miyazaki; individualCount: 1; **Taxon:** scientificName: Pseudorasbora pumila; **Location:** country: Japan; stateProvince: Iwate; locality: irrigation pond of the Kubo River Basin; **Identification:** identifiedBy: Yusuke Miyazaki; dateIdentified: 2010; **Event:** year: 2010; month: 9; day: 5; **Record Level:** basisOfRecord: PreservedSpecimen
- **Type status:** Other material. **Occurrence:** catalogNumber: KPM-NI 31770; recordedBy: Yusuke Miyazaki; individualCount: 1; **Taxon:** scientificName: Pseudorasbora pumila; **Location:** country: Japan; stateProvince: Iwate; locality: irrigation pond of the Kubo River Basin; **Identification:** identifiedBy: Yusuke Miyazaki; dateIdentified: 2010; **Event:** year: 2010; month: 9; day: 5; **Record Level:** basisOfRecord: PreservedSpecimen
- **Type status:** Other material. **Occurrence:** catalogNumber: KPM-NI 31771; recordedBy: Yusuke Miyazaki; individualCount: 1; **Taxon:** scientificName: Pseudorasbora pumila; **Location:** country: Japan; stateProvince: Iwate; locality: irrigation pond of the Kubo River Basin; **Identification:** identifiedBy: Yusuke Miyazaki; dateIdentified: 2010; **Event:** year: 2010; month: 9; day: 5; **Record Level:** basisOfRecord: PreservedSpecimen
- **Type status:** Other material. **Occurrence:** catalogNumber: KPM-NI 31772; recordedBy: Yusuke Miyazaki; individualCount: 1; **Taxon:** scientificName: Pseudorasbora pumila; **Location:** country: Japan; stateProvince: Iwate; locality: irrigation pond of the Kubo River Basin; **Identification:** identifiedBy: Yusuke Miyazaki; dateIdentified: 2010; **Event:** year: 2010; month: 9; day: 5; **Record Level:** basisOfRecord: PreservedSpecimen
- **Type status:** Other material. **Occurrence:** catalogNumber: KPM-NI 31773; recordedBy: Yusuke Miyazaki; individualCount: 1; **Taxon:** scientificName: Pseudorasbora pumila; **Location:** country: Japan; stateProvince: Iwate; locality: irrigation pond of the Kubo River Basin; **Identification:** identifiedBy: Yusuke Miyazaki; dateIdentified: 2010; **Event:** year: 2010; month: 9; day: 5; **Record Level:** basisOfRecord: PreservedSpecimen
- **Type status:** Other material. **Occurrence:** catalogNumber: KPM-NI 31774; recordedBy: Yusuke Miyazaki; individualCount: 1; **Taxon:** scientificName: Pseudorasbora pumila; **Location:** country: Japan; stateProvince: Iwate; locality: irrigation pond of the Kubo River Basin; **Identification:** identifiedBy: Yusuke Miyazaki; dateIdentified: 2010; **Event:** year: 2010; month: 9; day: 5; **Record Level:** basisOfRecord: PreservedSpecimen
- **Type status:** Other material. **Occurrence:** catalogNumber: KPM-NI 31775; recordedBy: Yusuke Miyazaki; individualCount: 1; **Taxon:** scientificName: Pseudorasbora pumila; **Location:** country: Japan; stateProvince: Iwate; locality: irrigation pond of the Kubo River Basin; **Identification:** identifiedBy: Yusuke Miyazaki; dateIdentified: 2010; **Event:** year: 2010; month: 9; day: 5; **Record Level:** basisOfRecord: PreservedSpecimen
- **Type status:** Other material. **Occurrence:** catalogNumber: KPM-NI 31776; recordedBy: Yusuke Miyazaki; individualCount: 1; **Taxon:** scientificName: Pseudorasbora pumila; **Location:** country: Japan; stateProvince: Iwate; locality: irrigation pond of the Kubo River Basin; **Identification:** identifiedBy: Yusuke Miyazaki; dateIdentified: 2010; **Event:** year: 2010; month: 9; day: 5; **Record Level:** basisOfRecord: PreservedSpecimen
- **Type status:** Other material. **Occurrence:** catalogNumber: KPM-NI 31777; recordedBy: Yusuke Miyazaki; individualCount: 1; **Taxon:** scientificName: Pseudorasbora pumila; **Location:** country: Japan; stateProvince: Iwate; locality: irrigation pond of the Kubo River Basin; **Identification:** identifiedBy: Yusuke Miyazaki; dateIdentified: 2010; **Event:** year: 2010; month: 9; day: 5; **Record Level:** basisOfRecord: PreservedSpecimen
- **Type status:** Other material. **Occurrence:** catalogNumber: KPM-NI 31778; recordedBy: Yusuke Miyazaki; individualCount: 1; **Taxon:** scientificName: Pseudorasbora pumila; **Location:** country: Japan; stateProvince: Iwate; locality: irrigation pond of the Kubo River Basin; **Identification:** identifiedBy: Yusuke Miyazaki; dateIdentified: 2010; **Event:** year: 2010; month: 9; day: 5; **Record Level:** basisOfRecord: PreservedSpecimen
- **Type status:** Other material. **Occurrence:** catalogNumber: KPM-NI 31779; recordedBy: Yusuke Miyazaki; individualCount: 1; **Taxon:** scientificName: Pseudorasbora pumila; **Location:** country: Japan; stateProvince: Iwate; locality: irrigation pond of the Kubo River Basin; **Identification:** identifiedBy: Yusuke Miyazaki; dateIdentified: 2010; **Event:** year: 2010; month: 9; day: 5; **Record Level:** basisOfRecord: PreservedSpecimen
- **Type status:** Other material. **Occurrence:** catalogNumber: KPM-NI 31780; recordedBy: Yusuke Miyazaki; individualCount: 1; **Taxon:** scientificName: Pseudorasbora pumila; **Location:** country: Japan; stateProvince: Iwate; locality: irrigation pond of the Kubo River Basin; **Identification:** identifiedBy: Yusuke Miyazaki; dateIdentified: 2010; **Event:** year: 2010; month: 9; day: 5; **Record Level:** basisOfRecord: PreservedSpecimen
- **Type status:** Other material. **Occurrence:** catalogNumber: KPM-NI 31781; recordedBy: Yusuke Miyazaki; individualCount: 1; **Taxon:** scientificName: Pseudorasbora pumila; **Location:** country: Japan; stateProvince: Iwate; locality: irrigation pond of the Kubo River Basin; **Identification:** identifiedBy: Yusuke Miyazaki; dateIdentified: 2010; **Event:** year: 2010; month: 9; day: 5; **Record Level:** basisOfRecord: PreservedSpecimen
- **Type status:** Other material. **Occurrence:** catalogNumber: KPM-NI 31782; recordedBy: Yusuke Miyazaki; individualCount: 1; **Taxon:** scientificName: Pseudorasbora pumila; **Location:** country: Japan; stateProvince: Iwate; locality: irrigation pond of the Kubo River Basin; **Identification:** identifiedBy: Yusuke Miyazaki; dateIdentified: 2010; **Event:** year: 2010; month: 9; day: 5; **Record Level:** basisOfRecord: PreservedSpecimen
- **Type status:** Other material. **Occurrence:** catalogNumber: KPM-NI 31783; recordedBy: Yusuke Miyazaki; individualCount: 1; **Taxon:** scientificName: Pseudorasbora pumila; **Location:** country: Japan; stateProvince: Iwate; locality: irrigation pond of the Kubo River Basin; **Identification:** identifiedBy: Yusuke Miyazaki; dateIdentified: 2010; **Event:** year: 2010; month: 9; day: 5; **Record Level:** basisOfRecord: PreservedSpecimen
- **Type status:** Other material. **Occurrence:** catalogNumber: KPM-NI 31784; recordedBy: Yusuke Miyazaki; individualCount: 1; **Taxon:** scientificName: Pseudorasbora pumila; **Location:** country: Japan; stateProvince: Iwate; locality: irrigation pond of the Kubo River Basin; **Identification:** identifiedBy: Yusuke Miyazaki; dateIdentified: 2010; **Event:** year: 2010; month: 9; day: 5; **Record Level:** basisOfRecord: PreservedSpecimen
- **Type status:** Other material. **Occurrence:** catalogNumber: KPM-NI 31785; recordedBy: Yusuke Miyazaki; individualCount: 1; **Taxon:** scientificName: Pseudorasbora pumila; **Location:** country: Japan; stateProvince: Iwate; locality: irrigation pond of the Kubo River Basin; **Identification:** identifiedBy: Yusuke Miyazaki; dateIdentified: 2010; **Event:** year: 2010; month: 9; day: 5; **Record Level:** basisOfRecord: PreservedSpecimen
- **Type status:** Other material. **Occurrence:** catalogNumber: KPM-NI 31786; recordedBy: Yusuke Miyazaki; individualCount: 1; **Taxon:** scientificName: Pseudorasbora pumila; **Location:** country: Japan; stateProvince: Iwate; locality: irrigation pond of the Kubo River Basin; **Identification:** identifiedBy: Yusuke Miyazaki; dateIdentified: 2010; **Event:** year: 2010; month: 9; day: 5; **Record Level:** basisOfRecord: PreservedSpecimen
- **Type status:** Other material. **Occurrence:** catalogNumber: KPM-NI 31787; recordedBy: Yusuke Miyazaki; individualCount: 1; **Taxon:** scientificName: Pseudorasbora pumila; **Location:** country: Japan; stateProvince: Iwate; locality: irrigation pond of the Kubo River Basin; **Identification:** identifiedBy: Yusuke Miyazaki; dateIdentified: 2010; **Event:** year: 2010; month: 9; day: 5; **Record Level:** basisOfRecord: PreservedSpecimen
- **Type status:** Other material. **Occurrence:** catalogNumber: KPM-NI 31788; recordedBy: Yusuke Miyazaki; individualCount: 1; **Taxon:** scientificName: Pseudorasbora pumila; **Location:** country: Japan; stateProvince: Iwate; locality: irrigation pond of the Kubo River Basin; **Identification:** identifiedBy: Yusuke Miyazaki; dateIdentified: 2010; **Event:** year: 2010; month: 9; day: 5; **Record Level:** basisOfRecord: PreservedSpecimen
- **Type status:** Other material. **Occurrence:** catalogNumber: KPM-NI 31789; recordedBy: Yusuke Miyazaki; individualCount: 1; **Taxon:** scientificName: Pseudorasbora pumila; **Location:** country: Japan; stateProvince: Iwate; locality: irrigation pond of the Kubo River Basin; **Identification:** identifiedBy: Yusuke Miyazaki; dateIdentified: 2010; **Event:** year: 2010; month: 9; day: 5; **Record Level:** basisOfRecord: PreservedSpecimen
- **Type status:** Other material. **Occurrence:** catalogNumber: KPM-NI 31790; recordedBy: Yusuke Miyazaki; individualCount: 1; **Taxon:** scientificName: Pseudorasbora pumila; **Location:** country: Japan; stateProvince: Iwate; locality: irrigation pond of the Kubo River Basin; **Identification:** identifiedBy: Yusuke Miyazaki; dateIdentified: 2010; **Event:** year: 2010; month: 9; day: 5; **Record Level:** basisOfRecord: PreservedSpecimen
- **Type status:** Other material. **Occurrence:** catalogNumber: KPM-NI 31791; recordedBy: Yusuke Miyazaki; individualCount: 1; **Taxon:** scientificName: Pseudorasbora pumila; **Location:** country: Japan; stateProvince: Iwate; locality: irrigation pond of the Kubo River Basin; **Identification:** identifiedBy: Yusuke Miyazaki; dateIdentified: 2010; **Event:** year: 2010; month: 9; day: 5; **Record Level:** basisOfRecord: PreservedSpecimen
- **Type status:** Other material. **Occurrence:** catalogNumber: KPM-NI 31792; recordedBy: Yusuke Miyazaki; individualCount: 1; **Taxon:** scientificName: Pseudorasbora pumila; **Location:** country: Japan; stateProvince: Iwate; locality: irrigation pond of the Kubo River Basin; **Identification:** identifiedBy: Yusuke Miyazaki; dateIdentified: 2010; **Event:** year: 2010; month: 9; day: 5; **Record Level:** basisOfRecord: PreservedSpecimen
- **Type status:** Other material. **Occurrence:** catalogNumber: KPM-NI 31793; recordedBy: Yusuke Miyazaki; individualCount: 1; **Taxon:** scientificName: Pseudorasbora pumila; **Location:** country: Japan; stateProvince: Iwate; locality: irrigation pond of the Kubo River Basin; **Identification:** identifiedBy: Yusuke Miyazaki; dateIdentified: 2010; **Event:** year: 2010; month: 9; day: 5; **Record Level:** basisOfRecord: PreservedSpecimen
- **Type status:** Other material. **Occurrence:** catalogNumber: KPM-NI 31794; recordedBy: Yusuke Miyazaki; individualCount: 1; **Taxon:** scientificName: Pseudorasbora pumila; **Location:** country: Japan; stateProvince: Iwate; locality: irrigation pond of the Kubo River Basin; **Identification:** identifiedBy: Yusuke Miyazaki; dateIdentified: 2010; **Event:** year: 2010; month: 9; day: 5; **Record Level:** basisOfRecord: PreservedSpecimen
- **Type status:** Other material. **Occurrence:** catalogNumber: KPM-NI 31795; recordedBy: Yusuke Miyazaki; individualCount: 1; **Taxon:** scientificName: Pseudorasbora pumila; **Location:** country: Japan; stateProvince: Iwate; locality: irrigation pond of the Kubo River Basin; **Identification:** identifiedBy: Yusuke Miyazaki; dateIdentified: 2010; **Event:** year: 2010; month: 9; day: 5; **Record Level:** basisOfRecord: PreservedSpecimen
- **Type status:** Other material. **Occurrence:** catalogNumber: KPM-NI 31796; recordedBy: Yusuke Miyazaki; individualCount: 1; **Taxon:** scientificName: Pseudorasbora pumila; **Location:** country: Japan; stateProvince: Iwate; locality: irrigation pond of the Kubo River Basin; **Identification:** identifiedBy: Yusuke Miyazaki; dateIdentified: 2010; **Event:** year: 2010; month: 9; day: 5; **Record Level:** basisOfRecord: PreservedSpecimen
- **Type status:** Other material. **Occurrence:** catalogNumber: KPM-NI 31797; recordedBy: Yusuke Miyazaki; individualCount: 1; **Taxon:** scientificName: Pseudorasbora pumila; **Location:** country: Japan; stateProvince: Iwate; locality: irrigation pond of the Kubo River Basin; **Identification:** identifiedBy: Yusuke Miyazaki; dateIdentified: 2010; **Event:** year: 2010; month: 9; day: 5; **Record Level:** basisOfRecord: PreservedSpecimen
- **Type status:** Other material. **Occurrence:** catalogNumber: KPM-NI 31798; recordedBy: Yusuke Miyazaki; individualCount: 1; **Taxon:** scientificName: Pseudorasbora pumila; **Location:** country: Japan; stateProvince: Iwate; locality: irrigation pond of the Kubo River Basin; **Identification:** identifiedBy: Yusuke Miyazaki; dateIdentified: 2010; **Event:** year: 2010; month: 9; day: 5; **Record Level:** basisOfRecord: PreservedSpecimen
- **Type status:** Other material. **Occurrence:** catalogNumber: KPM-NI 31799; recordedBy: Yusuke Miyazaki; individualCount: 1; **Taxon:** scientificName: Pseudorasbora pumila; **Location:** country: Japan; stateProvince: Iwate; locality: irrigation pond of the Kubo River Basin; **Identification:** identifiedBy: Yusuke Miyazaki; dateIdentified: 2010; **Event:** year: 2010; month: 9; day: 5; **Record Level:** basisOfRecord: PreservedSpecimen
- **Type status:** Other material. **Occurrence:** catalogNumber: KPM-NI 31800; recordedBy: Yusuke Miyazaki; individualCount: 1; **Taxon:** scientificName: Pseudorasbora pumila; **Location:** country: Japan; stateProvince: Iwate; locality: irrigation pond of the Kubo River Basin; **Identification:** identifiedBy: Yusuke Miyazaki; dateIdentified: 2010; **Event:** year: 2010; month: 9; day: 5; **Record Level:** basisOfRecord: PreservedSpecimen
- **Type status:** Other material. **Occurrence:** catalogNumber: KPM-NI 31801; recordedBy: Yusuke Miyazaki; individualCount: 1; **Taxon:** scientificName: Pseudorasbora pumila; **Location:** country: Japan; stateProvince: Iwate; locality: irrigation pond of the Kubo River Basin; **Identification:** identifiedBy: Yusuke Miyazaki; dateIdentified: 2010; **Event:** year: 2010; month: 9; day: 5; **Record Level:** basisOfRecord: PreservedSpecimen
- **Type status:** Other material. **Occurrence:** catalogNumber: KPM-NI 31802; recordedBy: Yusuke Miyazaki; individualCount: 1; **Taxon:** scientificName: Pseudorasbora pumila; **Location:** country: Japan; stateProvince: Iwate; locality: irrigation pond of the Kubo River Basin; **Identification:** identifiedBy: Yusuke Miyazaki; dateIdentified: 2010; **Event:** year: 2010; month: 9; day: 5; **Record Level:** basisOfRecord: PreservedSpecimen
- **Type status:** Other material. **Occurrence:** catalogNumber: KPM-NI 31803; recordedBy: Yusuke Miyazaki; individualCount: 1; **Taxon:** scientificName: Pseudorasbora pumila; **Location:** country: Japan; stateProvince: Iwate; locality: irrigation pond of the Kubo River Basin; **Identification:** identifiedBy: Yusuke Miyazaki; dateIdentified: 2010; **Event:** year: 2010; month: 9; day: 5; **Record Level:** basisOfRecord: PreservedSpecimen
- **Type status:** Other material. **Occurrence:** catalogNumber: KPM-NI 31804; recordedBy: Yusuke Miyazaki; individualCount: 1; **Taxon:** scientificName: Pseudorasbora pumila; **Location:** country: Japan; stateProvince: Iwate; locality: irrigation pond of the Kubo River Basin; **Identification:** identifiedBy: Yusuke Miyazaki; dateIdentified: 2010; **Event:** year: 2010; month: 9; day: 5; **Record Level:** basisOfRecord: PreservedSpecimen
- **Type status:** Other material. **Occurrence:** catalogNumber: KPM-NI 31805; recordedBy: Yusuke Miyazaki; individualCount: 1; **Taxon:** scientificName: Pseudorasbora pumila; **Location:** country: Japan; stateProvince: Iwate; locality: irrigation pond of the Kubo River Basin; **Identification:** identifiedBy: Yusuke Miyazaki; dateIdentified: 2010; **Event:** year: 2010; month: 9; day: 5; **Record Level:** basisOfRecord: PreservedSpecimen
- **Type status:** Other material. **Occurrence:** catalogNumber: KPM-NI 31806; recordedBy: Yusuke Miyazaki; individualCount: 1; **Taxon:** scientificName: Pseudorasbora pumila; **Location:** country: Japan; stateProvince: Iwate; locality: irrigation pond of the Kubo River Basin; **Identification:** identifiedBy: Yusuke Miyazaki; dateIdentified: 2010; **Event:** year: 2010; month: 9; day: 5; **Record Level:** basisOfRecord: PreservedSpecimen
- **Type status:** Other material. **Occurrence:** catalogNumber: KPM-NI 31807; recordedBy: Yusuke Miyazaki; individualCount: 1; **Taxon:** scientificName: Pseudorasbora pumila; **Location:** country: Japan; stateProvince: Iwate; locality: irrigation pond of the Kubo River Basin; **Identification:** identifiedBy: Yusuke Miyazaki; dateIdentified: 2010; **Event:** year: 2010; month: 9; day: 5; **Record Level:** basisOfRecord: PreservedSpecimen
- **Type status:** Other material. **Occurrence:** catalogNumber: KPM-NI 31808; recordedBy: Yusuke Miyazaki; individualCount: 1; **Taxon:** scientificName: Pseudorasbora pumila; **Location:** country: Japan; stateProvince: Iwate; locality: irrigation pond of the Kubo River Basin; **Identification:** identifiedBy: Yusuke Miyazaki; dateIdentified: 2010; **Event:** year: 2010; month: 9; day: 5; **Record Level:** basisOfRecord: PreservedSpecimen
- **Type status:** Other material. **Occurrence:** catalogNumber: KPM-NI 31809; recordedBy: Yusuke Miyazaki; individualCount: 1; **Taxon:** scientificName: Pseudorasbora pumila; **Location:** country: Japan; stateProvince: Iwate; locality: irrigation pond of the Kubo River Basin; **Identification:** identifiedBy: Yusuke Miyazaki; dateIdentified: 2010; **Event:** year: 2010; month: 9; day: 5; **Record Level:** basisOfRecord: PreservedSpecimen
- **Type status:** Other material. **Occurrence:** catalogNumber: KPM-NI 31810; recordedBy: Yusuke Miyazaki; individualCount: 1; **Taxon:** scientificName: Pseudorasbora pumila; **Location:** country: Japan; stateProvince: Iwate; locality: irrigation pond of the Kubo River Basin; **Identification:** identifiedBy: Yusuke Miyazaki; dateIdentified: 2010; **Event:** year: 2010; month: 9; day: 5; **Record Level:** basisOfRecord: PreservedSpecimen
- **Type status:** Other material. **Occurrence:** catalogNumber: KPM-NI 31811; recordedBy: Yusuke Miyazaki; individualCount: 1; **Taxon:** scientificName: Pseudorasbora pumila; **Location:** country: Japan; stateProvince: Iwate; locality: irrigation pond of the Kubo River Basin; **Identification:** identifiedBy: Yusuke Miyazaki; dateIdentified: 2010; **Event:** year: 2010; month: 9; day: 5; **Record Level:** basisOfRecord: PreservedSpecimen
- **Type status:** Other material. **Occurrence:** catalogNumber: KPM-NI 35077; recordedBy: Yusuke Miyazaki; individualCount: 1; **Taxon:** scientificName: Pseudorasbora pumila; **Location:** country: Japan; stateProvince: Iwate; locality: irrigation pond of the Kubo River Basin; **Identification:** identifiedBy: Yusuke Miyazaki; dateIdentified: 2010; **Event:** year: 2010; month: 9; day: 4; **Record Level:** basisOfRecord: PreservedSpecimen
- **Type status:** Other material. **Occurrence:** catalogNumber: KPM-NI 35078; recordedBy: Yusuke Miyazaki; individualCount: 1; **Taxon:** scientificName: Pseudorasbora pumila; **Location:** country: Japan; stateProvince: Iwate; locality: irrigation pond of the Kubo River Basin; **Identification:** identifiedBy: Yusuke Miyazaki; dateIdentified: 2010; **Event:** year: 2010; month: 9; day: 4; **Record Level:** basisOfRecord: PreservedSpecimen
- **Type status:** Other material. **Occurrence:** catalogNumber: KPM-NI 35079; recordedBy: Yusuke Miyazaki; individualCount: 1; **Taxon:** scientificName: Pseudorasbora pumila; **Location:** country: Japan; stateProvince: Iwate; locality: irrigation pond of the Kubo River Basin; **Identification:** identifiedBy: Yusuke Miyazaki; dateIdentified: 2010; **Event:** year: 2010; month: 9; day: 4; **Record Level:** basisOfRecord: PreservedSpecimen
- **Type status:** Other material. **Occurrence:** catalogNumber: KPM-NI 35080; recordedBy: Yusuke Miyazaki; individualCount: 1; **Taxon:** scientificName: Pseudorasbora pumila; **Location:** country: Japan; stateProvince: Iwate; locality: irrigation pond of the Kubo River Basin; **Identification:** identifiedBy: Yusuke Miyazaki; dateIdentified: 2010; **Event:** year: 2010; month: 9; day: 4; **Record Level:** basisOfRecord: PreservedSpecimen
- **Type status:** Other material. **Occurrence:** catalogNumber: KPM-NI 35081; recordedBy: Yusuke Miyazaki; individualCount: 1; **Taxon:** scientificName: Pseudorasbora pumila; **Location:** country: Japan; stateProvince: Iwate; locality: irrigation pond of the Kubo River Basin; **Identification:** identifiedBy: Yusuke Miyazaki; dateIdentified: 2010; **Event:** year: 2010; month: 9; day: 4; **Record Level:** basisOfRecord: PreservedSpecimen
- **Type status:** Other material. **Occurrence:** catalogNumber: KPM-NI 35082; recordedBy: Yusuke Miyazaki; individualCount: 1; **Taxon:** scientificName: Pseudorasbora pumila; **Location:** country: Japan; stateProvince: Iwate; locality: irrigation pond of the Kubo River Basin; **Identification:** identifiedBy: Yusuke Miyazaki; dateIdentified: 2010; **Event:** year: 2010; month: 9; day: 4; **Record Level:** basisOfRecord: PreservedSpecimen
- **Type status:** Other material. **Occurrence:** catalogNumber: KPM-NI 35083; recordedBy: Yusuke Miyazaki; individualCount: 1; **Taxon:** scientificName: Pseudorasbora pumila; **Location:** country: Japan; stateProvince: Iwate; locality: irrigation pond of the Kubo River Basin; **Identification:** identifiedBy: Yusuke Miyazaki; dateIdentified: 2010; **Event:** year: 2010; month: 9; day: 4; **Record Level:** basisOfRecord: PreservedSpecimen
- **Type status:** Other material. **Occurrence:** catalogNumber: KPM-NI 35084; recordedBy: Yusuke Miyazaki; individualCount: 1; **Taxon:** scientificName: Pseudorasbora pumila; **Location:** country: Japan; stateProvince: Iwate; locality: irrigation pond of the Kubo River Basin; **Identification:** identifiedBy: Yusuke Miyazaki; dateIdentified: 2010; **Event:** year: 2010; month: 9; day: 4; **Record Level:** basisOfRecord: PreservedSpecimen
- **Type status:** Other material. **Occurrence:** catalogNumber: KPM-NI 35085; recordedBy: Yusuke Miyazaki; individualCount: 1; **Taxon:** scientificName: Pseudorasbora pumila; **Location:** country: Japan; stateProvince: Iwate; locality: irrigation pond of the Kubo River Basin; **Identification:** identifiedBy: Yusuke Miyazaki; dateIdentified: 2010; **Event:** year: 2010; month: 9; day: 4; **Record Level:** basisOfRecord: PreservedSpecimen
- **Type status:** Other material. **Occurrence:** catalogNumber: KPM-NI 35086; recordedBy: Yusuke Miyazaki; individualCount: 1; **Taxon:** scientificName: Pseudorasbora pumila; **Location:** country: Japan; stateProvince: Iwate; locality: irrigation pond of the Kubo River Basin; **Identification:** identifiedBy: Yusuke Miyazaki; dateIdentified: 2010; **Event:** year: 2010; month: 9; day: 4; **Record Level:** basisOfRecord: PreservedSpecimen
- **Type status:** Other material. **Occurrence:** catalogNumber: KPM-NI 35087; recordedBy: Yusuke Miyazaki; individualCount: 1; **Taxon:** scientificName: Pseudorasbora pumila; **Location:** country: Japan; stateProvince: Iwate; locality: irrigation pond of the Kubo River Basin; **Identification:** identifiedBy: Yusuke Miyazaki; dateIdentified: 2010; **Event:** year: 2010; month: 9; day: 4; **Record Level:** basisOfRecord: PreservedSpecimen
- **Type status:** Other material. **Occurrence:** catalogNumber: KPM-NI 35088; recordedBy: Yusuke Miyazaki; individualCount: 1; **Taxon:** scientificName: Pseudorasbora pumila; **Location:** country: Japan; stateProvince: Iwate; locality: irrigation pond of the Kubo River Basin; **Identification:** identifiedBy: Yusuke Miyazaki; dateIdentified: 2010; **Event:** year: 2010; month: 9; day: 4; **Record Level:** basisOfRecord: PreservedSpecimen
- **Type status:** Other material. **Occurrence:** catalogNumber: KPM-NI 35089; recordedBy: Yusuke Miyazaki; individualCount: 1; **Taxon:** scientificName: Pseudorasbora pumila; **Location:** country: Japan; stateProvince: Iwate; locality: irrigation pond of the Kubo River Basin; **Identification:** identifiedBy: Yusuke Miyazaki; dateIdentified: 2010; **Event:** year: 2010; month: 9; day: 4; **Record Level:** basisOfRecord: PreservedSpecimen
- **Type status:** Other material. **Occurrence:** catalogNumber: KPM-NI 35090; recordedBy: Yusuke Miyazaki; individualCount: 1; **Taxon:** scientificName: Pseudorasbora pumila; **Location:** country: Japan; stateProvince: Iwate; locality: irrigation pond of the Kubo River Basin; **Identification:** identifiedBy: Yusuke Miyazaki; dateIdentified: 2010; **Event:** year: 2010; month: 9; day: 4; **Record Level:** basisOfRecord: PreservedSpecimen
- **Type status:** Other material. **Occurrence:** catalogNumber: KPM-NI 35091; recordedBy: Yusuke Miyazaki; individualCount: 1; **Taxon:** scientificName: Pseudorasbora pumila; **Location:** country: Japan; stateProvince: Iwate; locality: irrigation pond of the Kubo River Basin; **Identification:** identifiedBy: Yusuke Miyazaki; dateIdentified: 2010; **Event:** year: 2010; month: 9; day: 4; **Record Level:** basisOfRecord: PreservedSpecimen
- **Type status:** Other material. **Occurrence:** catalogNumber: KPM-NI 35092; recordedBy: Yusuke Miyazaki; individualCount: 1; **Taxon:** scientificName: Pseudorasbora pumila; **Location:** country: Japan; stateProvince: Iwate; locality: irrigation pond of the Kubo River Basin; **Identification:** identifiedBy: Yusuke Miyazaki; dateIdentified: 2010; **Event:** year: 2010; month: 9; day: 4; **Record Level:** basisOfRecord: PreservedSpecimen
- **Type status:** Other material. **Occurrence:** catalogNumber: KPM-NI 35093; recordedBy: Yusuke Miyazaki; individualCount: 1; **Taxon:** scientificName: Pseudorasbora pumila; **Location:** country: Japan; stateProvince: Iwate; locality: irrigation pond of the Kubo River Basin; **Identification:** identifiedBy: Yusuke Miyazaki; dateIdentified: 2010; **Event:** year: 2010; month: 9; day: 4; **Record Level:** basisOfRecord: PreservedSpecimen
- **Type status:** Other material. **Occurrence:** catalogNumber: KPM-NI 35094; recordedBy: Yusuke Miyazaki; individualCount: 1; **Taxon:** scientificName: Pseudorasbora pumila; **Location:** country: Japan; stateProvince: Iwate; locality: irrigation pond of the Kubo River Basin; **Identification:** identifiedBy: Yusuke Miyazaki; dateIdentified: 2010; **Event:** year: 2010; month: 9; day: 4; **Record Level:** basisOfRecord: PreservedSpecimen
- **Type status:** Other material. **Occurrence:** catalogNumber: KPM-NI 35095; recordedBy: Yusuke Miyazaki; individualCount: 1; **Taxon:** scientificName: Pseudorasbora pumila; **Location:** country: Japan; stateProvince: Iwate; locality: irrigation pond of the Kubo River Basin; **Identification:** identifiedBy: Yusuke Miyazaki; dateIdentified: 2010; **Event:** year: 2010; month: 9; day: 4; **Record Level:** basisOfRecord: PreservedSpecimen
- **Type status:** Other material. **Occurrence:** catalogNumber: KPM-NI 35096; recordedBy: Yusuke Miyazaki; individualCount: 1; **Taxon:** scientificName: Pseudorasbora pumila; **Location:** country: Japan; stateProvince: Iwate; locality: irrigation pond of the Kubo River Basin; **Identification:** identifiedBy: Yusuke Miyazaki; dateIdentified: 2010; **Event:** year: 2010; month: 9; day: 4; **Record Level:** basisOfRecord: PreservedSpecimen
- **Type status:** Other material. **Occurrence:** catalogNumber: KPM-NI 35097; recordedBy: Yusuke Miyazaki; individualCount: 1; **Taxon:** scientificName: Pseudorasbora pumila; **Location:** country: Japan; stateProvince: Iwate; locality: irrigation pond of the Kubo River Basin; **Identification:** identifiedBy: Yusuke Miyazaki; dateIdentified: 2010; **Event:** year: 2010; month: 9; day: 4; **Record Level:** basisOfRecord: PreservedSpecimen
- **Type status:** Other material. **Occurrence:** catalogNumber: KPM-NI 35098; recordedBy: Yusuke Miyazaki; individualCount: 1; **Taxon:** scientificName: Pseudorasbora pumila; **Location:** country: Japan; stateProvince: Iwate; locality: irrigation pond of the Kubo River Basin; **Identification:** identifiedBy: Yusuke Miyazaki; dateIdentified: 2010; **Event:** year: 2010; month: 9; day: 4; **Record Level:** basisOfRecord: PreservedSpecimen
- **Type status:** Other material. **Occurrence:** catalogNumber: KPM-NI 35099; recordedBy: Yusuke Miyazaki; individualCount: 1; **Taxon:** scientificName: Pseudorasbora pumila; **Location:** country: Japan; stateProvince: Iwate; locality: irrigation pond of the Kubo River Basin; **Identification:** identifiedBy: Yusuke Miyazaki; dateIdentified: 2010; **Event:** year: 2010; month: 9; day: 4; **Record Level:** basisOfRecord: PreservedSpecimen
- **Type status:** Other material. **Occurrence:** catalogNumber: KPM-NI 35100; recordedBy: Yusuke Miyazaki; individualCount: 1; **Taxon:** scientificName: Pseudorasbora pumila; **Location:** country: Japan; stateProvince: Iwate; locality: irrigation pond of the Kubo River Basin; **Identification:** identifiedBy: Yusuke Miyazaki; dateIdentified: 2010; **Event:** year: 2010; month: 9; day: 4; **Record Level:** basisOfRecord: PreservedSpecimen
- **Type status:** Other material. **Occurrence:** catalogNumber: KPM-NI 35101; recordedBy: Yusuke Miyazaki; individualCount: 1; **Taxon:** scientificName: Pseudorasbora pumila; **Location:** country: Japan; stateProvince: Iwate; locality: irrigation pond of the Kubo River Basin; **Identification:** identifiedBy: Yusuke Miyazaki; dateIdentified: 2010; **Event:** year: 2010; month: 9; day: 4; **Record Level:** basisOfRecord: PreservedSpecimen
- **Type status:** Other material. **Occurrence:** catalogNumber: KPM-NI 35102; recordedBy: Yusuke Miyazaki; individualCount: 1; **Taxon:** scientificName: Pseudorasbora pumila; **Location:** country: Japan; stateProvince: Iwate; locality: irrigation pond of the Kubo River Basin; **Identification:** identifiedBy: Yusuke Miyazaki; dateIdentified: 2010; **Event:** year: 2010; month: 9; day: 4; **Record Level:** basisOfRecord: PreservedSpecimen
- **Type status:** Other material. **Occurrence:** catalogNumber: KPM-NI 35103; recordedBy: Yusuke Miyazaki; individualCount: 1; **Taxon:** scientificName: Pseudorasbora pumila; **Location:** country: Japan; stateProvince: Iwate; locality: irrigation pond of the Kubo River Basin; **Identification:** identifiedBy: Yusuke Miyazaki; dateIdentified: 2010; **Event:** year: 2010; month: 9; day: 4; **Record Level:** basisOfRecord: PreservedSpecimen
- **Type status:** Other material. **Occurrence:** catalogNumber: KPM-NI 35104; recordedBy: Yusuke Miyazaki; individualCount: 1; **Taxon:** scientificName: Pseudorasbora pumila; **Location:** country: Japan; stateProvince: Iwate; locality: irrigation pond of the Kubo River Basin; **Identification:** identifiedBy: Yusuke Miyazaki; dateIdentified: 2010; **Event:** year: 2010; month: 9; day: 4; **Record Level:** basisOfRecord: PreservedSpecimen
- **Type status:** Other material. **Occurrence:** catalogNumber: KPM-NI 35105; recordedBy: Yusuke Miyazaki; individualCount: 1; **Taxon:** scientificName: Pseudorasbora pumila; **Location:** country: Japan; stateProvince: Iwate; locality: irrigation pond of the Kubo River Basin; **Identification:** identifiedBy: Yusuke Miyazaki; dateIdentified: 2010; **Event:** year: 2010; month: 9; day: 4; **Record Level:** basisOfRecord: PreservedSpecimen

##### Ecological interactions

###### Conservation status

National: CR (Ministry of the Environment, Japan 2013); Prefectural: CR+EN (Iwate Prefecture 2014).

##### Distribution

Japan.

##### Notes

This taxon is identical to *Pseudorasbora pumila pumila* of Hosoya (2013), in light of a potential new subspecies of *Pseudorasbora pumila* being recognized from Nagano, Shizuoka, Aichi, and Gifu Prefectures (Hosoya 2002, Hosoya 2013). This taxon was only recorded in irrigation ponds rich in aquatic plants.

#### Pseudogobio esocinus esocinus

(Temminck & Schlegel, 1846)

##### Materials

- **Type status:** Other material. **Occurrence:** catalogNumber: KPM-NI 22252; recordedBy: Yusuke Miyazaki; individualCount: 1; **Taxon:** scientificName: Pseudogobio esocinus esocinus; **Location:** country: Japan; stateProvince: Iwate; locality: Ichinono River; verbatimLatitude: 38°54′17.9″N; verbatimLongitude: 141°02′42.1″E; **Identification:** identifiedBy: Yusuke Miyazaki; dateIdentified: 2008; **Event:** year: 2008; month: 7; day: 8; **Record Level:** basisOfRecord: PreservedSpecimen
- **Type status:** Other material. **Occurrence:** catalogNumber: KPM-NI 22253; recordedBy: Yusuke Miyazaki; individualCount: 1; **Taxon:** scientificName: Pseudogobio esocinus esocinus; **Location:** country: Japan; stateProvince: Iwate; locality: Ichinono River; verbatimLatitude: 38°53′53.0″N; verbatimLongitude: 141°01′16.8″E; **Identification:** identifiedBy: Yusuke Miyazaki; dateIdentified: 2008; **Event:** year: 2008; month: 7; day: 7; **Record Level:** basisOfRecord: PreservedSpecimen
- **Type status:** Other material. **Occurrence:** catalogNumber: KPM-NI 23646; recordedBy: Yusuke Miyazaki; individualCount: 1; **Taxon:** scientificName: Pseudogobio esocinus esocinus; **Location:** country: Japan; stateProvince: Iwate; locality: Tochikura River; verbatimLatitude: 38°54′29.3″N; verbatimLongitude: 141°02′52.8″E; **Identification:** identifiedBy: Yusuke Miyazaki; dateIdentified: 2008; **Event:** year: 2008; month: 7; day: 11; **Record Level:** basisOfRecord: PreservedSpecimen
- **Type status:** Other material. **Occurrence:** catalogNumber: KPM-NI 23650; recordedBy: Yusuke Miyazaki; individualCount: 1; **Taxon:** scientificName: Pseudogobio esocinus esocinus; **Location:** country: Japan; stateProvince: Iwate; locality: Ichinono River; verbatimLatitude: 38°54′17.9″N; verbatimLongitude: 141°02′42.1″E; **Identification:** identifiedBy: Yusuke Miyazaki; dateIdentified: 2008; **Event:** year: 2008; month: 7; day: 8; **Record Level:** basisOfRecord: PreservedSpecimen
- **Type status:** Other material. **Occurrence:** catalogNumber: KPM-NI 23651; recordedBy: Yusuke Miyazaki; individualCount: 1; **Taxon:** scientificName: Pseudogobio esocinus esocinus; **Location:** country: Japan; stateProvince: Iwate; locality: Ichinono River; verbatimLatitude: 38°54′17.9″N; verbatimLongitude: 141°02′42.1″E; **Identification:** identifiedBy: Yusuke Miyazaki; dateIdentified: 2008; **Event:** year: 2008; month: 7; day: 8; **Record Level:** basisOfRecord: PreservedSpecimen
- **Type status:** Other material. **Occurrence:** catalogNumber: NSMT-P 91642; recordedBy: Yusuke Miyazaki; individualCount: 2; **Taxon:** scientificName: Pseudogobio esocinus esocinus; **Location:** country: Japan; stateProvince: Iwate; locality: Ichinono River; **Identification:** identifiedBy: Yusuke Miyazaki; dateIdentified: 2008; **Event:** year: 2008; month: 7; day: 8; **Record Level:** basisOfRecord: PreservedSpecimen
- **Type status:** Other material. **Occurrence:** catalogNumber: NSMT-P 96845; recordedBy: Yusuke Miyazaki and Yoko Takata; individualCount: 1; **Taxon:** scientificName: Pseudogobio esocinus esocinus; **Location:** country: Japan; stateProvince: Iwate; locality: Ichinono River; **Identification:** identifiedBy: Takashi P. Satoh; dateIdentified: 2010; **Event:** year: 2008; month: 10; day: 9; **Record Level:** basisOfRecord: PreservedSpecimen

##### Ecological interactions

###### Conservation status

Prefectural: DD (Iwate Prefecture 2014).

##### Distribution

China, Korea and Japan.

##### Notes

This species was only collected from the lotic environmentsin the present study.

#### Misgurnus anguillicaudatus

(Cantor, 1842)

##### Materials

- **Type status:** Other material. **Occurrence:** catalogNumber: KPM-NI 19453; recordedBy: Yusuke Miyazaki; individualCount: 1; **Taxon:** scientificName: Misgurnus anguillicaudatus; **Location:** country: Japan; stateProvince: Iwate; locality: irrigation pond of the Ichinono River Basin; verbatimLatitude: 38°56′10″N; verbatimLongitude: 141°01′33″E; **Identification:** identifiedBy: Yusuke Miyazaki; dateIdentified: 2007; **Event:** year: 2007; month: 9; day: 11; **Record Level:** basisOfRecord: PreservedSpecimen
- **Type status:** Other material. **Occurrence:** catalogNumber: KPM-NI 21191; recordedBy: Takumi Senou; individualCount: 1; **Taxon:** scientificName: Misgurnus anguillicaudatus; **Location:** country: Japan; stateProvince: Iwate; locality: irrigation pond of the Ichinono River Basin; verbatimLatitude: 38°54′43.05″N; verbatimLongitude: 141°01′24.39″E; **Identification:** identifiedBy: Hiroshi Senou; dateIdentified: 2008; **Event:** year: 2008; month: 5; day: 2; **Record Level:** basisOfRecord: PreservedSpecimen
- **Type status:** Other material. **Occurrence:** catalogNumber: KPM-NI 21217; recordedBy: Hiroshi Senou and Takumi Senou; individualCount: 7; **Taxon:** scientificName: Misgurnus anguillicaudatus; **Location:** country: Japan; stateProvince: Iwate; locality: irrigation pond of the Kubo River Basin; verbatimLatitude: 38°56′34.29″N; verbatimLongitude: 141°00′42.59″E; **Identification:** identifiedBy: Hiroshi Senou; dateIdentified: 2008; **Event:** year: 2008; month: 5; day: 2; **Record Level:** basisOfRecord: PreservedSpecimen
- **Type status:** Other material. **Occurrence:** catalogNumber: KPM-NI 21221; recordedBy: Hiroshi Senou and Takumi Senou; individualCount: 1; **Taxon:** scientificName: Misgurnus anguillicaudatus; **Location:** country: Japan; stateProvince: Iwate; locality: Tochikura River; verbatimLatitude: 38°54′58.75″N; verbatimLongitude: 140°58′49.07″E; **Identification:** identifiedBy: Hiroshi Senou; dateIdentified: 2008; **Event:** year: 2008; month: 5; day: 2; **Record Level:** basisOfRecord: PreservedSpecimen
- **Type status:** Other material. **Occurrence:** catalogNumber: KPM-NI 21223; recordedBy: Hiroshi Senou and Takumi Senou; individualCount: 4; **Taxon:** scientificName: Misgurnus anguillicaudatus; **Location:** country: Japan; stateProvince: Iwate; locality: irrigation pond of the Kubo River Basin; verbatimLatitude: 38°54′53.66″N; verbatimLongitude: 141°00′02.14″E; **Identification:** identifiedBy: Hiroshi Senou; dateIdentified: 2008; **Event:** year: 2008; month: 5; day: 2; **Record Level:** basisOfRecord: PreservedSpecimen
- **Type status:** Other material. **Occurrence:** catalogNumber: KPM-NI 21226; recordedBy: Yusuke Miyazaki and Hiroshi Senou; individualCount: 2; **Taxon:** scientificName: Misgurnus anguillicaudatus; **Location:** country: Japan; stateProvince: Iwate; locality: Tochikura River; verbatimLatitude: 38°55′28.28″N; verbatimLongitude: 141°00′27.29″E; **Identification:** identifiedBy: Yusuke Miyazaki; dateIdentified: 2008; **Event:** year: 2008; month: 5; day: 3; **Record Level:** basisOfRecord: PreservedSpecimen
- **Type status:** Other material. **Occurrence:** catalogNumber: KPM-NI 21399; recordedBy: Shin-ichi Suda; individualCount: 1; **Taxon:** scientificName: Misgurnus anguillicaudatus; **Location:** country: Japan; stateProvince: Iwate; locality: channel of rice paddy, Tochikura River Basin; verbatimLatitude: 38°54′19″N; verbatimLongitude: 141°02′41″E; **Identification:** identifiedBy: Yusuke Miyazaki; dateIdentified: 2008; **Event:** year: 2008; month: 5; day: 3; **Record Level:** basisOfRecord: PreservedSpecimen
- **Type status:** Other material. **Occurrence:** catalogNumber: KPM-NI 22323; recordedBy: Satoru Hirotani; individualCount: 1; **Taxon:** scientificName: Misgurnus anguillicaudatus; **Location:** country: Japan; stateProvince: Iwate; locality: the irrigation pond of the Kubo River Basin; verbatimLatitude: 38°53′48.9″N; verbatimLongitude: 141°01′43.5″E; **Identification:** identifiedBy: Yusuke Miyazaki; dateIdentified: 2008; **Event:** year: 2008; month: 9; day: 14; **Record Level:** basisOfRecord: PreservedSpecimen
- **Type status:** Other material. **Occurrence:** catalogNumber: KPM-NI 22274; recordedBy: Yusuke Miyazaki; individualCount: 1; **Taxon:** scientificName: Misgurnus anguillicaudatus; **Location:** country: Japan; stateProvince: Iwate; locality: Ichinono River; verbatimLatitude: 38°53′48.4″N; verbatimLongitude: 141°01′30.2″E; **Identification:** identifiedBy: Yusuke Miyazaki; dateIdentified: 2008; **Event:** year: 2008; month: 9; day: 14; **Record Level:** basisOfRecord: PreservedSpecimen
- **Type status:** Other material. **Occurrence:** catalogNumber: KPM-NI 22295; recordedBy: Yusuke Miyazaki; individualCount: 1; **Taxon:** scientificName: Misgurnus anguillicaudatus; **Location:** country: Japan; stateProvince: Iwate; locality: irrigation pond of the Ichinono River Basin; verbatimLatitude: 38°54′15.1″N; verbatimLongitude: 141°00′27.9″E; **Identification:** identifiedBy: Yusuke Miyazaki; dateIdentified: 2008; **Event:** year: 2008; month: 9; day: 17; **Record Level:** basisOfRecord: PreservedSpecimen
- **Type status:** Other material. **Occurrence:** catalogNumber: KPM-NI 22296; recordedBy: Yusuke Miyazaki; individualCount: 1; **Taxon:** scientificName: Misgurnus anguillicaudatus; **Location:** country: Japan; stateProvince: Iwate; locality: irrigation pond of the Ichinono River Basin; verbatimLatitude: 38°54′15.1″N; verbatimLongitude: 141°00′27.9″E; **Identification:** identifiedBy: Yusuke Miyazaki; dateIdentified: 2008; **Event:** year: 2008; month: 9; day: 17; **Record Level:** basisOfRecord: PreservedSpecimen
- **Type status:** Other material. **Occurrence:** catalogNumber: KPM-NI 22297; recordedBy: Yusuke Miyazaki; individualCount: 1; **Taxon:** scientificName: Misgurnus anguillicaudatus; **Location:** country: Japan; stateProvince: Iwate; locality: irrigation pond of the Tochikura River Basin; verbatimLatitude: 38°55′46.9″N; verbatimLongitude: 140°53′16.2″E; **Identification:** identifiedBy: Yusuke Miyazaki; dateIdentified: 2008; **Event:** year: 2008; month: 8; day: 24; **Record Level:** basisOfRecord: PreservedSpecimen
- **Type status:** Other material. **Occurrence:** catalogNumber: KPM-NI 22261; recordedBy: Yusuke Miyazaki; individualCount: 1; **Taxon:** scientificName: Misgurnus anguillicaudatus; **Location:** country: Japan; stateProvince: Iwate; locality: irrigation pond of the Tochikura River Basin; verbatimLatitude: 38°56′01.6″N; verbatimLongitude: 141°01′01.0″E; **Identification:** identifiedBy: Yusuke Miyazaki; dateIdentified: 2008; **Event:** year: 2008; month: 7; day: 11; **Record Level:** basisOfRecord: PreservedSpecimen
- **Type status:** Other material. **Occurrence:** catalogNumber: KPM-NI 22271; recordedBy: Yusuke Miyazaki; individualCount: 1; **Taxon:** scientificName: Misgurnus anguillicaudatus; **Location:** country: Japan; stateProvince: Iwate; locality: irrigation pond of the Kubo River Basin; verbatimLatitude: 38°56′33.1″N; verbatimLongitude: 141°00′46.1″E; **Identification:** identifiedBy: Yusuke Miyazaki; dateIdentified: 2008; **Event:** year: 2008; month: 8; day: 26; **Record Level:** basisOfRecord: PreservedSpecimen
- **Type status:** Other material. **Occurrence:** catalogNumber: KPM-NI 22268; recordedBy: Yusuke Miyazaki; individualCount: 1; **Taxon:** scientificName: Misgurnus anguillicaudatus; **Location:** country: Japan; stateProvince: Iwate; locality: Kubo River; verbatimLatitude: 38°55′40″N; verbatimLongitude: 140°59′51″E; **Identification:** identifiedBy: Yusuke Miyazaki; dateIdentified: 2008; **Event:** year: 2008; month: 8; day: 24; **Record Level:** basisOfRecord: PreservedSpecimen
- **Type status:** Other material. **Occurrence:** catalogNumber: KPM-NI 22294; recordedBy: Yusuke Miyazaki; individualCount: 1; **Taxon:** scientificName: Misgurnus anguillicaudatus; **Location:** country: Japan; stateProvince: Iwate; locality: irrigation pond of the Kubo River Basin; verbatimLatitude: 38°55′46.1″N; verbatimLongitude: 140°53′16.2″E; **Identification:** identifiedBy: Yusuke Miyazaki; dateIdentified: 2008; **Event:** year: 2008; month: 8; day: 25; **Record Level:** basisOfRecord: PreservedSpecimen
- **Type status:** Other material. **Occurrence:** catalogNumber: KPM-NI 22282; recordedBy: Yusuke Miyazaki; individualCount: 1; **Taxon:** scientificName: Misgurnus anguillicaudatus; **Location:** country: Japan; stateProvince: Iwate; locality: irrigation pond of the Kubo River Basin; verbatimLatitude: 38°55′19.4″N; verbatimLongitude: 140°59′29.1″E; **Identification:** identifiedBy: Yusuke Miyazaki; dateIdentified: 2008; **Event:** year: 2008; month: 8; day: 27; **Record Level:** basisOfRecord: PreservedSpecimen
- **Type status:** Other material. **Occurrence:** catalogNumber: KPM-NI 22288; recordedBy: Yusuke Miyazaki; individualCount: 1; **Taxon:** scientificName: Misgurnus anguillicaudatus; **Location:** country: Japan; stateProvince: Iwate; locality: irrigation pond of the Kubo River Basin; verbatimLatitude: 38°55′20.2″N; verbatimLongitude: 140°59′35.7″E; **Identification:** identifiedBy: Yusuke Miyazaki; dateIdentified: 2008; **Event:** year: 2008; month: 8; day: 28; **Record Level:** basisOfRecord: PreservedSpecimen
- **Type status:** Other material. **Occurrence:** catalogNumber: KPM-NI 22303; recordedBy: Yusuke Miyazaki; individualCount: 1; **Taxon:** scientificName: Misgurnus anguillicaudatus; **Location:** country: Japan; stateProvince: Iwate; locality: irrigation pond of the Kubo River Basin; verbatimLatitude: 38°55′17.3″N; verbatimLongitude: 140°59′26.7″E; **Identification:** identifiedBy: Yusuke Miyazaki; dateIdentified: 2008; **Event:** year: 2008; month: 8; day: 27; **Record Level:** basisOfRecord: PreservedSpecimen
- **Type status:** Other material. **Occurrence:** catalogNumber: KPM-NI 22292; recordedBy: Yusuke Miyazaki; individualCount: 1; **Taxon:** scientificName: Misgurnus anguillicaudatus; **Location:** country: Japan; stateProvince: Iwate; locality: irrigation pond of the Kubo River Basin; verbatimLatitude: 38°55′49″N; verbatimLongitude: 141°01′35″E; **Identification:** identifiedBy: Yusuke Miyazaki; dateIdentified: 2008; **Event:** year: 2008; month: 8; day: 30; **Record Level:** basisOfRecord: PreservedSpecimen
- **Type status:** Other material. **Occurrence:** catalogNumber: KPM-NI 22304; recordedBy: Yusuke Miyazaki; individualCount: 1; **Taxon:** scientificName: Misgurnus anguillicaudatus; **Location:** country: Japan; stateProvince: Iwate; locality: irrigation pond of the Kubo River Basin; verbatimLatitude: 38°55′50″N; verbatimLongitude: 141°01′34″E; **Identification:** identifiedBy: Yusuke Miyazaki; dateIdentified: 2008; **Event:** year: 2008; month: 8; day: 30; **Record Level:** basisOfRecord: PreservedSpecimen
- **Type status:** Other material. **Occurrence:** catalogNumber: KPM-NI 23952; recordedBy: Yusuke Miyazaki; individualCount: 1; **Taxon:** scientificName: Misgurnus anguillicaudatus; **Location:** country: Japan; stateProvince: Iwate; locality: irrigation pond of the Kubo River Basin; verbatimLatitude: 38°55′22″N; verbatimLongitude: 140°59′46″E; **Identification:** identifiedBy: Yusuke Miyazaki; dateIdentified: 2009; **Event:** year: 2009; month: 5; day: 2; **Record Level:** basisOfRecord: PreservedSpecimen
- **Type status:** Other material. **Occurrence:** catalogNumber: KPM-NI 23953; recordedBy: Yusuke Miyazaki; individualCount: 1; **Taxon:** scientificName: Misgurnus anguillicaudatus; **Location:** country: Japan; stateProvince: Iwate; locality: irrigation pond of the Kubo River Basin; verbatimLatitude: 38°55′08″N; verbatimLongitude: 141°01′05″E; **Identification:** identifiedBy: Yusuke Miyazaki; dateIdentified: 2009; **Event:** year: 2009; month: 5; day: 15; **Record Level:** basisOfRecord: PreservedSpecimen
- **Type status:** Other material. **Occurrence:** catalogNumber: KPM-NI 23954; recordedBy: Yusuke Miyazaki; individualCount: 1; **Taxon:** scientificName: Misgurnus anguillicaudatus; **Location:** country: Japan; stateProvince: Iwate; locality: irrigation pond of the Kubo River Basin; verbatimLatitude: 38°55′14″N; verbatimLongitude: 141°01′10″E; **Identification:** identifiedBy: Yusuke Miyazaki; dateIdentified: 2009; **Event:** year: 2009; month: 5; day: 19; **Record Level:** basisOfRecord: PreservedSpecimen
- **Type status:** Other material. **Occurrence:** catalogNumber: KPM-NI 23955; recordedBy: Yusuke Miyazaki; individualCount: 1; **Taxon:** scientificName: Misgurnus anguillicaudatus; **Location:** country: Japan; stateProvince: Iwate; locality: irrigation pond of the Kubo River Basin; verbatimLatitude: 38°54′54″N; verbatimLongitude: 141°03′55″E; **Identification:** identifiedBy: Yusuke Miyazaki; dateIdentified: 2009; **Event:** year: 2009; month: 5; day: 19; **Record Level:** basisOfRecord: PreservedSpecimen
- **Type status:** Other material. **Occurrence:** catalogNumber: KPM-NI 23956; recordedBy: Yusuke Miyazaki; individualCount: 1; **Taxon:** scientificName: Misgurnus anguillicaudatus; **Location:** country: Japan; stateProvince: Iwate; locality: irrigation pond of the Kubo River Basin; verbatimLatitude: 38°56′38″N; verbatimLongitude: 141°00′48″E; **Identification:** identifiedBy: Yusuke Miyazaki; dateIdentified: 2009; **Event:** year: 2009; month: 5; day: 15; **Record Level:** basisOfRecord: PreservedSpecimen
- **Type status:** Other material. **Occurrence:** catalogNumber: KPM-NI 23957; recordedBy: Yusuke Miyazaki; individualCount: 1; **Taxon:** scientificName: Misgurnus anguillicaudatus; **Location:** country: Japan; stateProvince: Iwate; locality: irrigation pond of the Kubo River Basin; verbatimLatitude: 38°54′37″N; verbatimLongitude: 141°00′34″E; **Identification:** identifiedBy: Yusuke Miyazaki; dateIdentified: 2009; **Event:** year: 2009; month: 5; day: 20; **Record Level:** basisOfRecord: PreservedSpecimen
- **Type status:** Other material. **Occurrence:** catalogNumber: KPM-NI 23958; recordedBy: Yusuke Miyazaki; individualCount: 1; **Taxon:** scientificName: Misgurnus anguillicaudatus; **Location:** country: Japan; stateProvince: Iwate; locality: irrigation pond of the Kubo River Basin; verbatimLatitude: 38°56′38″N; verbatimLongitude: 141°00′48″E; **Identification:** identifiedBy: Yusuke Miyazaki; dateIdentified: 2009; **Event:** year: 2009; month: 5; day: 2; **Record Level:** basisOfRecord: PreservedSpecimen
- **Type status:** Other material. **Occurrence:** catalogNumber: KPM-NI 23959; recordedBy: Yusuke Miyazaki; individualCount: 1; **Taxon:** scientificName: Misgurnus anguillicaudatus; **Location:** country: Japan; stateProvince: Iwate; locality: irrigation pond of the Kubo River Basin; verbatimLatitude: 38°55′33″N; verbatimLongitude: 141°01′53″E; **Identification:** identifiedBy: Yusuke Miyazaki; dateIdentified: 2009; **Event:** year: 2009; month: 8; day: 2; **Record Level:** basisOfRecord: PreservedSpecimen
- **Type status:** Other material. **Occurrence:** catalogNumber: KPM-NI 23960; recordedBy: Yusuke Miyazaki; individualCount: 1; **Taxon:** scientificName: Misgurnus anguillicaudatus; **Location:** country: Japan; stateProvince: Iwate; locality: irrigation pond of the Kubo River Basin; verbatimLatitude: 38°54′34″N; verbatimLongitude: 141°00′34″E; **Identification:** identifiedBy: Yusuke Miyazaki; dateIdentified: 2009; **Event:** year: 2009; month: 5; day: 20; **Record Level:** basisOfRecord: PreservedSpecimen
- **Type status:** Other material. **Occurrence:** catalogNumber: KPM-NI 23961; recordedBy: Yusuke Miyazaki; individualCount: 1; **Taxon:** scientificName: Misgurnus anguillicaudatus; **Location:** country: Japan; stateProvince: Iwate; locality: irrigation pond of the Kubo River Basin; verbatimLatitude: 38°56′45″N; verbatimLongitude: 141°00′08″E; **Identification:** identifiedBy: Yusuke Miyazaki; dateIdentified: 2009; **Event:** year: 2009; month: 5; day: 2; **Record Level:** basisOfRecord: PreservedSpecimen
- **Type status:** Other material. **Occurrence:** catalogNumber: KPM-NI 23962; recordedBy: Yusuke Miyazaki; individualCount: 1; **Taxon:** scientificName: Misgurnus anguillicaudatus; **Location:** country: Japan; stateProvince: Iwate; locality: irrigation pond of the Kubo River Basin; verbatimLatitude: 38°54′37″N; verbatimLongitude: 141°00′35″E; **Identification:** identifiedBy: Yusuke Miyazaki; dateIdentified: 2009; **Event:** year: 2009; month: 5; day: 20; **Record Level:** basisOfRecord: PreservedSpecimen
- **Type status:** Other material. **Occurrence:** catalogNumber: KPM-NI 23963; recordedBy: Yusuke Miyazaki; individualCount: 1; **Taxon:** scientificName: Misgurnus anguillicaudatus; **Location:** country: Japan; stateProvince: Iwate; locality: irrigation pond of the Kubo River Basin; verbatimLatitude: 38°55′14″N; verbatimLongitude: 141°01′13″E; **Identification:** identifiedBy: Yusuke Miyazaki; dateIdentified: 2009; **Event:** year: 2009; month: 5; day: 3; **Record Level:** basisOfRecord: PreservedSpecimen
- **Type status:** Other material. **Occurrence:** catalogNumber: KPM-NI 24399; recordedBy: Yusuke Miyazaki; individualCount: 1; **Taxon:** scientificName: Misgurnus anguillicaudatus; **Location:** country: Japan; stateProvince: Iwate; locality: irrigation pond of the Kubo River Basin; verbatimLatitude: 38°56′09″N; verbatimLongitude: 141°01′34″E; **Identification:** identifiedBy: Yusuke Miyazaki; dateIdentified: 2009; **Event:** year: 2009; month: 9; day: 22; **Record Level:** basisOfRecord: PreservedSpecimen
- **Type status:** Other material. **Occurrence:** catalogNumber: KPM-NI 24406; recordedBy: Shogo Nishihara; individualCount: 1; **Taxon:** scientificName: Misgurnus anguillicaudatus; **Location:** country: Japan; stateProvince: Iwate; locality: irrigation pond of the Kubo River Basin; verbatimLatitude: 38°56′32″N; verbatimLongitude: 141°01′57″E; **Identification:** identifiedBy: Yusuke Miyazaki; dateIdentified: 2009; **Event:** year: 2009; month: 9; day: 20; **Record Level:** basisOfRecord: PreservedSpecimen
- **Type status:** Other material. **Occurrence:** catalogNumber: KPM-NI 24412; recordedBy: Yusuke Miyazaki; individualCount: 1; **Taxon:** scientificName: Misgurnus anguillicaudatus; **Location:** country: Japan; stateProvince: Iwate; municipality: Dougasawa, Hagishou; verbatimLatitude: 38°55′49″N; verbatimLongitude: 141°01′57″E; **Identification:** identifiedBy: Yusuke Miyazaki; dateIdentified: 2009; **Event:** year: 2009; month: 9; day: 20; **Record Level:** basisOfRecord: PreservedSpecimen
- **Type status:** Other material. **Occurrence:** catalogNumber: KPM-NI 24416; recordedBy: Yusuke Miyazaki and Shogo Nishihara; individualCount: 1; **Taxon:** scientificName: Misgurnus anguillicaudatus; **Location:** country: Japan; stateProvince: Iwate; municipality: Dougasawa, Hagishou; verbatimLatitude: 38°54′31″N; verbatimLongitude: 141°00′48″E; **Identification:** identifiedBy: Yusuke Miyazaki; dateIdentified: 2009; **Event:** year: 2009; month: 9; day: 21; **Record Level:** basisOfRecord: PreservedSpecimen
- **Type status:** Other material. **Occurrence:** catalogNumber: KPM-NI 24417; recordedBy: Yusuke Miyazaki; individualCount: 1; **Taxon:** scientificName: Misgurnus anguillicaudatus; **Location:** country: Japan; stateProvince: Iwate; locality: irrigation pond of the Kubo River Basin; verbatimLatitude: 38°54′32″N; verbatimLongitude: 141°00′50″E; **Identification:** identifiedBy: Yusuke Miyazaki; dateIdentified: 2009; **Event:** year: 2009; month: 9; day: 21; **Record Level:** basisOfRecord: PreservedSpecimen
- **Type status:** Other material. **Occurrence:** catalogNumber: KPM-NI 24420; recordedBy: Yusuke Miyazaki; individualCount: 1; **Taxon:** scientificName: Misgurnus anguillicaudatus; **Location:** country: Japan; stateProvince: Iwate; locality: channel of rice paddy, Tochikura River Basin; verbatimLatitude: 38°56′12″N; verbatimLongitude: 141°02′03″E; **Identification:** identifiedBy: Yusuke Miyazaki; dateIdentified: 2009; **Event:** year: 2009; month: 9; day: 20; **Record Level:** basisOfRecord: PreservedSpecimen
- **Type status:** Other material. **Occurrence:** catalogNumber: KPM-NI 24462; recordedBy: Yusuke Miyazaki; individualCount: 1; **Taxon:** scientificName: Misgurnus anguillicaudatus; **Location:** country: Japan; stateProvince: Iwate; locality: irrigation pond of the Tochikura River Basin; verbatimLatitude: 38°56′12″N; verbatimLongitude: 141°02′03″E; **Identification:** identifiedBy: Yusuke Miyazaki; dateIdentified: 2009; **Event:** year: 2009; month: 9; day: 21; **Record Level:** basisOfRecord: PreservedSpecimen
- **Type status:** Other material. **Occurrence:** catalogNumber: KPM-NI 24468; recordedBy: Yusuke Miyazaki; individualCount: 1; **Taxon:** scientificName: Misgurnus anguillicaudatus; **Location:** country: Japan; stateProvince: Iwate; municipality: Dougasawa, Hagishou; verbatimLatitude: 38°56′32″N; verbatimLongitude: 140°59′49″E; **Identification:** identifiedBy: Yusuke Miyazaki; dateIdentified: 2009; **Event:** year: 2009; month: 9; day: 22; **Record Level:** basisOfRecord: PreservedSpecimen
- **Type status:** Other material. **Occurrence:** catalogNumber: KPM-NI 24988; recordedBy: Yusuke Miyazaki; individualCount: 1; **Taxon:** scientificName: Misgurnus anguillicaudatus; **Location:** country: Japan; stateProvince: Iwate; municipality: Dougasawa, Hagishou; verbatimLatitude: 38°54′57″N; verbatimLongitude: 141°01′53″E; **Identification:** identifiedBy: Yusuke Miyazaki; dateIdentified: 2009; **Event:** year: 2008; month: 9; day: 18; **Record Level:** basisOfRecord: PreservedSpecimen
- **Type status:** Other material. **Occurrence:** catalogNumber: KPM-NI 24990; recordedBy: Yusuke Miyazaki; individualCount: 1; **Taxon:** scientificName: Misgurnus anguillicaudatus; **Location:** country: Japan; stateProvince: Iwate; locality: irrigation pond of the Kubo River Basin; verbatimLatitude: 38°56′16″N; verbatimLongitude: 141°01′00″E; **Identification:** identifiedBy: Yusuke Miyazaki; dateIdentified: 2009; **Event:** year: 2008; month: 10; day: 8; **Record Level:** basisOfRecord: PreservedSpecimen
- **Type status:** Other material. **Occurrence:** catalogNumber: NSMT-P 90708; recordedBy: Yusuke Miyazaki; individualCount: 1; **Taxon:** scientificName: Misgurnus anguillicaudatus; **Location:** country: Japan; stateProvince: Iwate; municipality: Hagishou; verbatimLatitude: 38°55′37″N; verbatimLongitude: 141°02′32″E; **Identification:** identifiedBy: Yusuke Miyazaki; dateIdentified: 2008; **Event:** year: 2008; month: 4; day: 30; **Record Level:** basisOfRecord: PreservedSpecimen
- **Type status:** Other material. **Occurrence:** catalogNumber: NSMT-P 90710; recordedBy: Yusuke Miyazaki; individualCount: 1; **Taxon:** scientificName: Misgurnus anguillicaudatus; **Location:** country: Japan; stateProvince: Iwate; municipality: Hagishou; verbatimLatitude: 38°55′48″N; verbatimLongitude: 141°02′19″E; **Identification:** identifiedBy: Yusuke Miyazaki; dateIdentified: 2008; **Event:** year: 2008; month: 5; day: 1; **Record Level:** basisOfRecord: PreservedSpecimen
- **Type status:** Other material. **Occurrence:** catalogNumber: NSMT-P 90715; recordedBy: Yusuke Miyazaki; individualCount: 2; **Taxon:** scientificName: Misgurnus anguillicaudatus; **Location:** country: Japan; stateProvince: Iwate; municipality: Hagishou; verbatimLatitude: 38°56′49″N; verbatimLongitude: 141°00′12″E; **Identification:** identifiedBy: Yusuke Miyazaki; dateIdentified: 2008; **Event:** year: 2008; month: 5; day: 1; **Record Level:** basisOfRecord: PreservedSpecimen
- **Type status:** Other material. **Occurrence:** catalogNumber: NSMT-P 90716; recordedBy: Yusuke Miyazaki; individualCount: 2; **Taxon:** scientificName: Misgurnus anguillicaudatus; **Location:** country: Japan; stateProvince: Iwate; municipality: Hagishou; verbatimLatitude: 38°55′46″N; verbatimLongitude: 140°57′15″E; **Identification:** identifiedBy: Yusuke Miyazaki; dateIdentified: 2008; **Event:** year: 2008; month: 5; day: 1; **Record Level:** basisOfRecord: PreservedSpecimen
- **Type status:** Other material. **Occurrence:** catalogNumber: NSMT-P 90717; recordedBy: Yusuke Miyazaki; individualCount: 1; **Taxon:** scientificName: Misgurnus anguillicaudatus; **Location:** country: Japan; stateProvince: Iwate; municipality: Hagishou; verbatimLatitude: 38°55′47″N; verbatimLongitude: 140°57′13″E; **Identification:** identifiedBy: Yusuke Miyazaki; dateIdentified: 2008; **Event:** year: 2008; month: 5; day: 1; **Record Level:** basisOfRecord: PreservedSpecimen
- **Type status:** Other material. **Occurrence:** catalogNumber: NSMT-P 90719; recordedBy: Yusuke Miyazaki; individualCount: 1; **Taxon:** scientificName: Misgurnus anguillicaudatus; **Location:** country: Japan; stateProvince: Iwate; municipality: Hagishou; verbatimLatitude: 38°55′55″N; verbatimLongitude: 141°02′03″E; **Identification:** identifiedBy: Yusuke Miyazaki; dateIdentified: 2008; **Event:** year: 2008; month: 5; day: 1; **Record Level:** basisOfRecord: PreservedSpecimen
- **Type status:** Other material. **Occurrence:** catalogNumber: NSMT-P 91194; recordedBy: Yusuke Miyazaki; individualCount: 1; **Taxon:** scientificName: Misgurnus anguillicaudatus; **Location:** country: Japan; stateProvince: Iwate; locality: Tochikura River; **Identification:** identifiedBy: Yusuke Miyazaki; dateIdentified: 2008; **Event:** year: 2008; month: 7; day: 4; **Record Level:** basisOfRecord: PreservedSpecimen
- **Type status:** Other material. **Occurrence:** catalogNumber: NSMT-P 91197; recordedBy: Yusuke Miyazaki; individualCount: 1; **Taxon:** scientificName: Misgurnus anguillicaudatus; **Location:** country: Japan; stateProvince: Iwate; locality: Kubo River; **Identification:** identifiedBy: Yusuke Miyazaki; dateIdentified: 2008; **Event:** year: 2008; month: 7; day: 7; **Record Level:** basisOfRecord: PreservedSpecimen
- **Type status:** Other material. **Occurrence:** catalogNumber: NSMT-P 91640; recordedBy: Yusuke Miyazaki; individualCount: 2; **Taxon:** scientificName: Misgurnus anguillicaudatus; **Location:** country: Japan; stateProvince: Iwate; locality: Ichinono River; **Identification:** identifiedBy: Yusuke Miyazaki; dateIdentified: 2008; **Event:** year: 2008; month: 7; day: 10; **Record Level:** basisOfRecord: PreservedSpecimen
- **Type status:** Other material. **Occurrence:** catalogNumber: NSMT-P 91645; recordedBy: Yusuke Miyazaki; individualCount: 1; **Taxon:** scientificName: Misgurnus anguillicaudatus; **Location:** country: Japan; stateProvince: Iwate; locality: Tochikura River; **Identification:** identifiedBy: Yusuke Miyazaki; dateIdentified: 2008; **Event:** year: 1967; month: 1; day: 11; **Record Level:** basisOfRecord: PreservedSpecimen
- **Type status:** Other material. **Occurrence:** catalogNumber: NSMT-P 96055; recordedBy: Yusuke Miyazaki; individualCount: 1; **Taxon:** scientificName: Misgurnus anguillicaudatus; **Location:** country: Japan; stateProvince: Iwate; locality: irrigation pond of the Kubo River Basin; verbatimLatitude: 38°55′54″N; verbatimLongitude: 141°01′35″E; **Identification:** identifiedBy: Yusuke Miyazaki; dateIdentified: 2009; **Event:** year: 2008; month: 8; day: 29; **Record Level:** basisOfRecord: PreservedSpecimen
- **Type status:** Other material. **Occurrence:** catalogNumber: NSMT-P 96060; recordedBy: Yusuke Miyazaki; individualCount: 1; **Taxon:** scientificName: Misgurnus anguillicaudatus; **Location:** country: Japan; stateProvince: Iwate; locality: irrigation pond of the Tochikura River Basin; verbatimLatitude: 38°54′36″N; verbatimLongitude: 141°01′07″E; **Identification:** identifiedBy: Yusuke Miyazaki; dateIdentified: 2009; **Event:** year: 2008; month: 8; day: 24; **Record Level:** basisOfRecord: PreservedSpecimen
- **Type status:** Other material. **Occurrence:** catalogNumber: NSMT-P 96843; recordedBy: Yusuke Miyazaki and Yoko Takata; individualCount: 2; **Taxon:** scientificName: Misgurnus anguillicaudatus; **Location:** country: Japan; stateProvince: Iwate; locality: Ichinono River; **Identification:** identifiedBy: Yoko Takata; dateIdentified: 2010; **Event:** year: 2008; month: 10; day: 9; **Record Level:** basisOfRecord: PreservedSpecimen
- **Type status:** Other material. **Occurrence:** catalogNumber: NSMT-P 96848; recordedBy: Yusuke Miyazaki; individualCount: 1; **Taxon:** scientificName: Misgurnus anguillicaudatus; **Location:** country: Japan; stateProvince: Iwate; locality: irrigation pond of the Kubo River Basin; **Identification:** identifiedBy: Yoko Takata; dateIdentified: 2010; **Event:** year: 2008; month: 10; day: 9; **Record Level:** basisOfRecord: PreservedSpecimen
- **Type status:** Other material. **Occurrence:** catalogNumber: NSMT-P 96849; recordedBy: Yusuke Miyazaki; individualCount: 5; **Taxon:** scientificName: Misgurnus anguillicaudatus; **Location:** country: Japan; stateProvince: Iwate; locality: irrigation pond of the Kubo River Basin; **Identification:** identifiedBy: Yoko Takata; dateIdentified: 2010; **Event:** year: 2008; month: 10; day: 9; **Record Level:** basisOfRecord: PreservedSpecimen
- **Type status:** Other material. **Occurrence:** catalogNumber: NSMT-P 96855; recordedBy: Yusuke Miyazaki and Yoko Takata; individualCount: 9; **Taxon:** scientificName: Misgurnus anguillicaudatus; **Location:** country: Japan; stateProvince: Iwate; locality: irrigation pond of the Kubo River Basin; **Identification:** identifiedBy: Yoko Takata; dateIdentified: 2010; **Event:** year: 2008; month: 10; day: 8; **Record Level:** basisOfRecord: PreservedSpecimen
- **Type status:** Other material. **Occurrence:** catalogNumber: NSMT-P 96860; recordedBy: Yusuke Miyazaki and Yoko Takata; individualCount: 2; **Taxon:** scientificName: Misgurnus anguillicaudatus; **Location:** country: Japan; stateProvince: Iwate; municipality: Hagishou; **Identification:** identifiedBy: Yoko Takata; dateIdentified: 2010; **Event:** year: 2008; month: 10; day: 8; **Record Level:** basisOfRecord: PreservedSpecimen
- **Type status:** Other material. **Occurrence:** catalogNumber: NSMT-P 97109; recordedBy: Yusuke Miyazaki and Yoko Takata; individualCount: 2; **Taxon:** scientificName: Misgurnus anguillicaudatus; **Location:** country: Japan; stateProvince: Iwate; locality: irrigation pond of the Kubo River Basin; **Identification:** identifiedBy: Yoko Takata; dateIdentified: 2010; **Event:** year: 2008; month: 10; day: 8; **Record Level:** basisOfRecord: PreservedSpecimen

##### Ecological interactions

###### Conservation status

National: DD (Ministry of the Environment, Japan 2013).

##### Distribution

Far East Asia.

##### Notes

This species was only recorded from the lentic environment of the region.

#### Cobitis biwae

Jordan & Snyder, 1901

##### Materials

- **Type status:** Other material. **Occurrence:** catalogNumber: KPM-NI 21190; recordedBy: Hiroshi Senou; individualCount: 1; **Taxon:** scientificName: Cobitis biwae; **Location:** country: Japan; stateProvince: Iwate; locality: Tochikura River; verbatimLatitude: 38°54′43.05″N; verbatimLongitude: 141°01′24.39″E; **Identification:** identifiedBy: Hiroshi Senou; dateIdentified: 2008; **Event:** year: 2008; month: 5; day: 2; **Record Level:** basisOfRecord: PreservedSpecimen
- **Type status:** Other material. **Occurrence:** catalogNumber: KPM-NI 21222; recordedBy: Hiroshi Senou and Takumi Senou; individualCount: 1; **Taxon:** scientificName: Cobitis biwae; **Location:** country: Japan; stateProvince: Iwate; locality: Tochikura River; verbatimLatitude: 38°54′58.75″N; verbatimLongitude: 140°58′49.07″E; **Identification:** identifiedBy: Hiroshi Senou; dateIdentified: 2008; **Event:** year: 2008; month: 5; day: 2; **Record Level:** basisOfRecord: PreservedSpecimen
- **Type status:** Other material. **Occurrence:** catalogNumber: KPM-NI 21224; recordedBy: Hiroshi Senou and Takumi Senou; individualCount: 3; **Taxon:** scientificName: Cobitis biwae; **Location:** country: Japan; stateProvince: Iwate; locality: channel of rice paddy, Tochikura River Basin; verbatimLatitude: 38°54′53.66″N; verbatimLongitude: 141°00′02.14″E; **Identification:** identifiedBy: Hiroshi Senou; dateIdentified: 2008; **Event:** year: 2008; month: 5; day: 2; **Record Level:** basisOfRecord: PreservedSpecimen
- **Type status:** Other material. **Occurrence:** catalogNumber: KPM-NI 21225; recordedBy: Hiroshi Senou and Takumi Senou; individualCount: 2; **Taxon:** scientificName: Cobitis biwae; **Location:** country: Japan; stateProvince: Iwate; locality: channel of rice paddy, Tochikura River Basin; verbatimLatitude: 38°54′53.66″N; verbatimLongitude: 141°00′02.14″E; **Identification:** identifiedBy: Hiroshi Senou; dateIdentified: 2008; **Event:** year: 2008; month: 5; day: 2; **Record Level:** basisOfRecord: PreservedSpecimen
- **Type status:** Other material. **Occurrence:** catalogNumber: KPM-NI 22262; recordedBy: Yusuke Miyazaki; individualCount: 1; **Taxon:** scientificName: Cobitis biwae; **Location:** country: Japan; stateProvince: Iwate; locality: Kubo River; verbatimLatitude: 38°56′01.6″N; verbatimLongitude: 141°01′01.0″E; **Identification:** identifiedBy: Yusuke Miyazaki; dateIdentified: 2008; **Event:** year: 2008; month: 7; day: 11; **Record Level:** basisOfRecord: PreservedSpecimen
- **Type status:** Other material. **Occurrence:** catalogNumber: KPM-NI 23999; recordedBy: Yusuke Miyazaki; individualCount: 3; **Taxon:** scientificName: Cobitis biwae; **Location:** country: Japan; stateProvince: Iwate; locality: Tochikura River; verbatimLatitude: 38°54′59″N; verbatimLongitude: 140°58′51″E; **Identification:** identifiedBy: Yusuke Miyazaki; dateIdentified: 2009; **Event:** year: 2009; month: 5; day: 1; **Record Level:** basisOfRecord: PreservedSpecimen
- **Type status:** Other material. **Occurrence:** catalogNumber: KPM-NI 24466; recordedBy: Yusuke Miyazaki; individualCount: 1; **Taxon:** scientificName: Cobitis biwae; **Location:** country: Japan; stateProvince: Iwate; locality: irrigation pond of the Kubo River Basin; verbatimLatitude: 38°56′32″N; verbatimLongitude: 140°59′49″E; **Identification:** identifiedBy: Yusuke Miyazaki; dateIdentified: 2009; **Event:** year: 2009; month: 9; day: 22; **Record Level:** basisOfRecord: PreservedSpecimen
- **Type status:** Other material. **Occurrence:** catalogNumber: NSMT-P 91189; individualCount: 1; **Taxon:** scientificName: Cobitis biwae; **Location:** country: Japan; stateProvince: Iwate; locality: Tochikura River; **Identification:** identifiedBy: Yusuke Miyazaki; dateIdentified: 2008; **Event:** year: 2008; month: 7; day: 4; **Record Level:** basisOfRecord: PreservedSpecimen
- **Type status:** Other material. **Occurrence:** catalogNumber: NSMT-P 91193; individualCount: 1; **Taxon:** scientificName: Cobitis biwae; **Location:** country: Japan; stateProvince: Iwate; locality: Tochikura River; **Identification:** identifiedBy: Yusuke Miyazaki; dateIdentified: 2008; **Event:** year: 2008; month: 7; day: 4; **Record Level:** basisOfRecord: PreservedSpecimen
- **Type status:** Other material. **Occurrence:** catalogNumber: NSMT-P 91196; individualCount: 1; **Taxon:** scientificName: Cobitis biwae; **Location:** country: Japan; stateProvince: Iwate; locality: Tochikura River; **Identification:** identifiedBy: Yusuke Miyazaki; dateIdentified: 2008; **Event:** year: 2008; month: 7; day: 5; **Record Level:** basisOfRecord: PreservedSpecimen
- **Type status:** Other material. **Occurrence:** catalogNumber: NSMT-P 91199; individualCount: 1; **Taxon:** scientificName: Cobitis biwae; **Location:** country: Japan; stateProvince: Iwate; locality: Ichinono River; **Identification:** identifiedBy: Yusuke Miyazaki; dateIdentified: 2008; **Event:** year: 2008; month: 7; day: 6; **Record Level:** basisOfRecord: PreservedSpecimen
- **Type status:** Other material. **Occurrence:** catalogNumber: NSMT-P 91639; individualCount: 1; **Taxon:** scientificName: Cobitis biwae; **Location:** country: Japan; stateProvince: Iwate; locality: Ichinono River; **Identification:** identifiedBy: Yusuke Miyazaki; dateIdentified: 2008; **Event:** year: 2008; month: 7; day: 7; **Record Level:** basisOfRecord: PreservedSpecimen
- **Type status:** Other material. **Occurrence:** catalogNumber: NSMT-P 91641; individualCount: 1; **Taxon:** scientificName: Cobitis biwae; **Location:** country: Japan; stateProvince: Iwate; locality: Ichinono River; **Identification:** identifiedBy: Yusuke Miyazaki; dateIdentified: 2008; **Event:** year: 2008; month: 7; day: 10; **Record Level:** basisOfRecord: PreservedSpecimen
- **Type status:** Other material. **Occurrence:** catalogNumber: NSMT-P 91644; individualCount: 1; **Taxon:** scientificName: Cobitis biwae; **Location:** country: Japan; stateProvince: Iwate; locality: Tochikura River; **Identification:** identifiedBy: Yusuke Miyazaki; dateIdentified: 2008; **Event:** year: 2008; month: 7; day: 9; **Record Level:** basisOfRecord: PreservedSpecimen
- **Type status:** Other material. **Occurrence:** catalogNumber: NSMT-P 91646; individualCount: 1; **Taxon:** scientificName: Cobitis biwae; **Location:** country: Japan; stateProvince: Iwate; locality: Tochikura River; **Identification:** identifiedBy: Yusuke Miyazaki; dateIdentified: 2008; **Event:** year: 2008; month: 7; day: 11; **Record Level:** basisOfRecord: PreservedSpecimen
- **Type status:** Other material. **Occurrence:** catalogNumber: NSMT-P 91652; individualCount: 1; **Taxon:** scientificName: Cobitis biwae; **Location:** country: Japan; stateProvince: Iwate; locality: Kubo River; **Identification:** identifiedBy: Yusuke Miyazaki; dateIdentified: 2008; **Event:** year: 2008; month: 7; day: 9; **Record Level:** basisOfRecord: PreservedSpecimen
- **Type status:** Other material. **Occurrence:** catalogNumber: NSMT-P 96056; individualCount: 1; **Taxon:** scientificName: Cobitis biwae; **Location:** country: Japan; stateProvince: Iwate; locality: irrigation pond of the Kubo River Basin; verbatimLatitude: 38°55′39″N; verbatimLongitude: 140°59′52″E; **Identification:** identifiedBy: Yusuke Miyazaki; dateIdentified: 2009; **Event:** year: 2008; month: 8; day: 30; **Record Level:** basisOfRecord: PreservedSpecimen
- **Type status:** Other material. **Occurrence:** catalogNumber: NSMT-P 96895; individualCount: 1; **Taxon:** scientificName: Cobitis biwae; **Location:** country: Japan; stateProvince: Iwate; locality: Tochikura River; **Identification:** identifiedBy: Yoko Takata; dateIdentified: 2010; **Event:** year: 2008; month: 10; day: 10; **Record Level:** basisOfRecord: PreservedSpecimen
- **Type status:** Other material. **Occurrence:** catalogNumber: NSMT-P 96896; individualCount: 5; **Taxon:** scientificName: Cobitis biwae; **Location:** country: Japan; stateProvince: Iwate; locality: Tochikura River; **Identification:** identifiedBy: Yoko Takata; dateIdentified: 2010; **Event:** year: 2008; month: 10; day: 10; **Record Level:** basisOfRecord: PreservedSpecimen

##### Distribution

Japan

##### Notes

This taxon is identical with *Cobitis* sp. BIWAE type C of Nakajima et al. (2012), and could represent a possibly undescribed species, although we refrain from referring to it as such until further work is carried out.

#### Tachysurus tokiensis

(Döderlein, 1887)

##### Materials

- **Type status:** Other material. **Occurrence:** catalogNumber: KPM-NI 19440; recordedBy: Yusuke Miyazaki; individualCount: 1; **Taxon:** scientificName: Tachysurus tokiensis; **Location:** country: Japan; stateProvince: Iwate; locality: irrigation pond of the Kubo River Basin; verbatimLatitude: 38°56′09″N; verbatimLongitude: 141°01′22″E; **Identification:** identifiedBy: Yusuke Miyazaki; dateIdentified: 2007; **Event:** year: 2007; month: 9; day: 11; **Record Level:** basisOfRecord: PreservedSpecimen
- **Type status:** Other material. **Occurrence:** catalogNumber: KPM-NI 21194; recordedBy: Yusuke Miyazaki; individualCount: 1; **Taxon:** scientificName: Tachysurus tokiensis; **Location:** country: Japan; stateProvince: Iwate; locality: Tochikura River; verbatimLatitude: 38°54′43.05″N; verbatimLongitude: 141°01′24.39″E; **Identification:** identifiedBy: Hiroshi Senou; dateIdentified: 2008; **Event:** year: 2008; month: 5; day: 2; **Record Level:** basisOfRecord: PreservedSpecimen
- **Type status:** Other material. **Occurrence:** catalogNumber: KPM-NI 21184; recordedBy: Yusuke Miyazaki; individualCount: 1; **Taxon:** scientificName: Tachysurus tokiensis; **Location:** country: Japan; stateProvince: Iwate; locality: Tochikura River; verbatimLatitude: 38°54′43″N; verbatimLongitude: 141°02′32″E; **Identification:** identifiedBy: Yusuke Miyazaki; dateIdentified: 2008; **Event:** year: 2008; month: 5; day: 3; **Record Level:** basisOfRecord: PreservedSpecimen
- **Type status:** Other material. **Occurrence:** catalogNumber: KPM-NI 21185; recordedBy: Shin-ichi Suda; individualCount: 1; **Taxon:** scientificName: Tachysurus tokiensis; **Location:** country: Japan; stateProvince: Iwate; locality: irrigation pond of the Tochikura River Basin; verbatimLatitude: 38°54′32″N; verbatimLongitude: 141°00′41″E; **Identification:** identifiedBy: Yusuke Miyazaki; dateIdentified: 2008; **Event:** year: 2008; month: 5; day: 3; **Record Level:** basisOfRecord: PreservedSpecimen
- **Type status:** Other material. **Occurrence:** catalogNumber: KPM-NI 22263; recordedBy: Shinichi Suda; individualCount: 1; **Taxon:** scientificName: Tachysurus tokiensis; **Location:** country: Japan; stateProvince: Iwate; locality: irrigation pond of the Kubo River Basin; verbatimLatitude: 38°56′49″N; verbatimLongitude: 141°00′11″E; **Identification:** identifiedBy: Yusuke Miyazaki; dateIdentified: 2008; **Event:** year: 2008; month: 7; day: 7; **Record Level:** basisOfRecord: PreservedSpecimen
- **Type status:** Other material. **Occurrence:** catalogNumber: KPM-NI 23639; recordedBy: Yusuke Miyazaki; individualCount: 1; **Taxon:** scientificName: Tachysurus tokiensis; **Location:** country: Japan; stateProvince: Iwate; locality: Ichinono River; verbatimLatitude: 38°53′53.0″N; verbatimLongitude: 141°01′16.8″E; **Identification:** identifiedBy: Yusuke Miyazaki; dateIdentified: 2009; **Event:** year: 2008; month: 7; day: 7; **Record Level:** basisOfRecord: PreservedSpecimen
- **Type status:** Other material. **Occurrence:** catalogNumber: KPM-NI 23640; recordedBy: Yusuke Miyazaki; individualCount: 1; **Taxon:** scientificName: Tachysurus tokiensis; **Location:** country: Japan; stateProvince: Iwate; locality: Ichinono River; verbatimLatitude: 38°53′53.0″N; verbatimLongitude: 141°01′16.8″E; **Identification:** identifiedBy: Yusuke Miyazaki; dateIdentified: 2009; **Event:** year: 2008; month: 7; day: 7; **Record Level:** basisOfRecord: PreservedSpecimen
- **Type status:** Other material. **Occurrence:** catalogNumber: KPM-NI 23641; recordedBy: Yusuke Miyazaki; individualCount: 1; **Taxon:** scientificName: Tachysurus tokiensis; **Location:** country: Japan; stateProvince: Iwate; locality: Ichinono River; verbatimLatitude: 38°53′53.0″N; verbatimLongitude: 141°01′16.8″E; **Identification:** identifiedBy: Yusuke Miyazaki; dateIdentified: 2009; **Event:** year: 2008; month: 7; day: 7; **Record Level:** basisOfRecord: PreservedSpecimen
- **Type status:** Other material. **Occurrence:** catalogNumber: KPM-NI 23642; recordedBy: Yusuke Miyazaki; individualCount: 1; **Taxon:** scientificName: Tachysurus tokiensis; **Location:** country: Japan; stateProvince: Iwate; locality: Kubo River; verbatimLatitude: 38°56′01.6″N; verbatimLongitude: 141°01′01.0″E; **Identification:** identifiedBy: Yusuke Miyazaki; dateIdentified: 2009; **Event:** year: 2008; month: 7; day: 11; **Record Level:** basisOfRecord: PreservedSpecimen
- **Type status:** Other material. **Occurrence:** catalogNumber: KPM-NI 23644; recordedBy: Yusuke Miyazaki; individualCount: 1; **Taxon:** scientificName: Tachysurus tokiensis; **Location:** country: Japan; stateProvince: Iwate; locality: Tochikura River; verbatimLatitude: 38°54′29.3″N; verbatimLongitude: 141°02′52.8″E; **Identification:** identifiedBy: Yusuke Miyazaki; dateIdentified: 2009; **Event:** year: 2008; month: 7; day: 11; **Record Level:** basisOfRecord: PreservedSpecimen
- **Type status:** Other material. **Occurrence:** catalogNumber: KPM-NI 23648; recordedBy: Yusuke Miyazaki; individualCount: 1; **Taxon:** scientificName: Tachysurus tokiensis; **Location:** country: Japan; stateProvince: Iwate; locality: Ichinono River; verbatimLatitude: 38°54′17.9″N; verbatimLongitude: 141°02′42.1″E; **Identification:** identifiedBy: Yusuke Miyazaki; dateIdentified: 2009; **Event:** year: 2008; month: 7; day: 8; **Record Level:** basisOfRecord: PreservedSpecimen
- **Type status:** Other material. **Occurrence:** catalogNumber: KPM-NI 23649; recordedBy: Yusuke Miyazaki; individualCount: 1; **Taxon:** scientificName: Tachysurus tokiensis; **Location:** country: Japan; stateProvince: Iwate; locality: Ichinono River; verbatimLatitude: 38°54′17.9″N; verbatimLongitude: 141°02′42.1″E; **Identification:** identifiedBy: Yusuke Miyazaki; dateIdentified: 2009; **Event:** year: 2008; month: 7; day: 8; **Record Level:** basisOfRecord: PreservedSpecimen
- **Type status:** Other material. **Occurrence:** catalogNumber: KPM-NI 23652; recordedBy: Yusuke Miyazaki; individualCount: 1; **Taxon:** scientificName: Tachysurus tokiensis; **Location:** country: Japan; stateProvince: Iwate; locality: Tochikura River; verbatimLatitude: 38°53′25.7″N; verbatimLongitude: 141°00′18.0″E; **Identification:** identifiedBy: Yusuke Miyazaki; dateIdentified: 2009; **Event:** year: 2008; month: 7; day: 8; **Record Level:** basisOfRecord: PreservedSpecimen
- **Type status:** Other material. **Occurrence:** catalogNumber: KPM-NI 23653; recordedBy: Yusuke Miyazaki; individualCount: 1; **Taxon:** scientificName: Tachysurus tokiensis; **Location:** country: Japan; stateProvince: Iwate; locality: Kubo River; verbatimLatitude: 38°54′55.4″N; verbatimLongitude: 141°04′23.7″E; **Identification:** identifiedBy: Yusuke Miyazaki; dateIdentified: 2009; **Event:** year: 2008; month: 7; day: 9; **Record Level:** basisOfRecord: PreservedSpecimen
- **Type status:** Other material. **Occurrence:** catalogNumber: KPM-NI 23738; recordedBy: Yusuke Miyazaki; individualCount: 1; **Taxon:** scientificName: Tachysurus tokiensis; **Location:** country: Japan; stateProvince: Iwate; locality: the irrigation pond of the Kubo River Basin; verbatimLatitude: 38°55′30″N; verbatimLongitude: 141°00′25″E; **Identification:** identifiedBy: Hiroshi Senou; dateIdentified: 2009; **Event:** year: 2009; month: 5; day: 14; **Record Level:** basisOfRecord: PreservedSpecimen
- **Type status:** Other material. **Occurrence:** catalogNumber: KPM-NI 23739; recordedBy: Yusuke Miyazaki; individualCount: 1; **Taxon:** scientificName: Tachysurus tokiensis; **Location:** country: Japan; stateProvince: Iwate; locality: irrigation pond of the Kubo River Basin; verbatimLatitude: 38°55′30″N; verbatimLongitude: 141°00′25″E; **Identification:** identifiedBy: Hiroshi Senou; dateIdentified: 2009; **Event:** year: 2009; month: 5; day: 14; **Record Level:** basisOfRecord: PreservedSpecimen
- **Type status:** Other material. **Occurrence:** catalogNumber: KPM-NI 24396; recordedBy: Yusuke Miyazaki; individualCount: 1; **Taxon:** scientificName: Tachysurus tokiensis; **Location:** country: Japan; stateProvince: Iwate; locality: irrigation pond of the Kubo River Basin; verbatimLatitude: 38°55′28″N; verbatimLongitude: 141°00′02″E; **Identification:** identifiedBy: Yusuke Miyazaki; dateIdentified: 2009; **Event:** year: 2009; month: 9; day: 24; **Record Level:** basisOfRecord: PreservedSpecimen
- **Type status:** Other material. **Occurrence:** catalogNumber: KPM-NI 24398; recordedBy: Yusuke Miyazaki; individualCount: 1; **Taxon:** scientificName: Tachysurus tokiensis; **Location:** country: Japan; stateProvince: Iwate; locality: irrigation pond of the Kubo River Basin; verbatimLatitude: 38°55′33″N; verbatimLongitude: 140°59′57″E; **Identification:** identifiedBy: Yusuke Miyazaki; dateIdentified: 2009; **Event:** year: 2009; month: 9; day: 22; **Record Level:** basisOfRecord: PreservedSpecimen
- **Type status:** Other material. **Occurrence:** catalogNumber: KPM-NI 24400; recordedBy: Yusuke Miyazaki and Shogo Nishihara; individualCount: 1; **Taxon:** scientificName: Tachysurus tokiensis; **Location:** country: Japan; stateProvince: Iwate; municipality: Dougasawa, Hagishou; verbatimLatitude: 38°55′33″N; verbatimLongitude: 140°59′58″E; **Identification:** identifiedBy: Yusuke Miyazaki; dateIdentified: 2009; **Event:** year: 2009; month: 9; day: 20; **Record Level:** basisOfRecord: PreservedSpecimen
- **Type status:** Other material. **Occurrence:** catalogNumber: KPM-NI 24401; recordedBy: Yusuke Miyazaki and Shogo Nishihara; individualCount: 1; **Taxon:** scientificName: Tachysurus tokiensis; **Location:** country: Japan; stateProvince: Iwate; municipality: Dougasawa, Hagishou; verbatimLatitude: 38°55′33″N; verbatimLongitude: 140°59′59″E; **Identification:** identifiedBy: Yusuke Miyazaki; dateIdentified: 2009; **Event:** year: 2009; month: 9; day: 20; **Record Level:** basisOfRecord: PreservedSpecimen
- **Type status:** Other material. **Occurrence:** catalogNumber: KPM-NI 24402; recordedBy: Yusuke Miyazaki and Shogo Nishihara; individualCount: 1; **Taxon:** scientificName: Tachysurus tokiensis; **Location:** country: Japan; stateProvince: Iwate; municipality: Dougasawa, Hagishou; verbatimLatitude: 38°55′33″N; verbatimLongitude: 140°59′60″E; **Identification:** identifiedBy: Yusuke Miyazaki; dateIdentified: 2009; **Event:** year: 2009; month: 9; day: 20; **Record Level:** basisOfRecord: PreservedSpecimen
- **Type status:** Other material. **Occurrence:** catalogNumber: KPM-NI 24403; recordedBy: Yusuke Miyazaki and Shogo Nishihara; individualCount: 1; **Taxon:** scientificName: Tachysurus tokiensis; **Location:** country: Japan; stateProvince: Iwate; municipality: Dougasawa, Hagishou; verbatimLatitude: 38°55′33″N; verbatimLongitude: 140°59′61″E; **Identification:** identifiedBy: Yusuke Miyazaki; dateIdentified: 2009; **Event:** year: 2009; month: 9; day: 20; **Record Level:** basisOfRecord: PreservedSpecimen
- **Type status:** Other material. **Occurrence:** catalogNumber: KPM-NI 24404; recordedBy: Yusuke Miyazaki and Shogo Nishihara; individualCount: 1; **Taxon:** scientificName: Tachysurus tokiensis; **Location:** country: Japan; stateProvince: Iwate; municipality: Dougasawa, Hagishou; verbatimLatitude: 38°55′33″N; verbatimLongitude: 140°59′62″E; **Identification:** identifiedBy: Yusuke Miyazaki; dateIdentified: 2009; **Event:** year: 2009; month: 9; day: 20; **Record Level:** basisOfRecord: PreservedSpecimen
- **Type status:** Other material. **Occurrence:** catalogNumber: KPM-NI 24469; recordedBy: Yusuke Miyazaki; individualCount: 1; **Taxon:** scientificName: Tachysurus tokiensis; **Location:** country: Japan; stateProvince: Iwate; locality: irrigation pond of the Kubo River Basin; verbatimLatitude: 38°56′15.9″N; verbatimLongitude: 141°01′01.0″E; **Identification:** identifiedBy: Yusuke Miyazaki; dateIdentified: 2009; **Event:** year: 2009; month: 9; day: 24; **Record Level:** basisOfRecord: PreservedSpecimen
- **Type status:** Other material. **Occurrence:** catalogNumber: KPM-NI 24470; recordedBy: Yusuke Miyazaki; individualCount: 1; **Taxon:** scientificName: Tachysurus tokiensis; **Location:** country: Japan; stateProvince: Iwate; locality: irrigation pond of the Kubo River Basin; verbatimLatitude: 38°56′15.9″N; verbatimLongitude: 141°01′01.1″E; **Identification:** identifiedBy: Yusuke Miyazaki; dateIdentified: 2009; **Event:** year: 2009; month: 9; day: 24; **Record Level:** basisOfRecord: PreservedSpecimen
- **Type status:** Other material. **Occurrence:** catalogNumber: KPM-NI 24991; recordedBy: Yusuke Miyazaki; individualCount: 1; **Taxon:** scientificName: Tachysurus tokiensis; **Location:** country: Japan; stateProvince: Iwate; locality: irrigation pond of the Kubo River Basin; verbatimLatitude: 38°56′16″N; verbatimLongitude: 141°01′00″E; **Identification:** identifiedBy: Yusuke Miyazaki; dateIdentified: 2009; **Event:** year: 2009; month: 10; day: 8; **Record Level:** basisOfRecord: PreservedSpecimen
- **Type status:** Other material. **Occurrence:** catalogNumber: KPM-NR 43911; recordedBy: Taichi Okeda; individualCount: 1; **Taxon:** scientificName: Tachysurus tokiensis; **Location:** country: Japan; stateProvince: Iwate; locality: irrigation pond of the Kubo River Basin; verbatimLatitude: 38°56′09″N; verbatimLongitude: 141°01′22″E; **Identification:** identifiedBy: Yusuke Miyazaki; dateIdentified: 2009; **Event:** year: 2009; month: 9; day: 3; **Record Level:** basisOfRecord: Photography
- **Type status:** Other material. **Occurrence:** catalogNumber: NSMT-P 91195; recordedBy: Yusuke Miyazaki; individualCount: 1; **Taxon:** scientificName: Tachysurus tokiensis; **Location:** country: Japan; stateProvince: Iwate; locality: Tochikura River; **Identification:** identifiedBy: Yusuke Miyazaki; dateIdentified: 2008; **Event:** year: 2008; month: 7; day: 5; **Record Level:** basisOfRecord: PreservedSpecimen
- **Type status:** Other material. **Occurrence:** catalogNumber: NSMT-P 96832; recordedBy: Yusuke Miyazaki; individualCount: 1; **Taxon:** scientificName: Tachysurus tokiensis; **Location:** country: Japan; stateProvince: Iwate; municipality: Hagishou; **Identification:** identifiedBy: Yoko Takata; dateIdentified: 2010; **Event:** year: 2008; month: 10; day: 8; **Record Level:** basisOfRecord: PreservedSpecimen
- **Type status:** Other material. **Occurrence:** catalogNumber: NSMT-P 96833; recordedBy: Yusuke Miyazaki; individualCount: 4; **Taxon:** scientificName: Tachysurus tokiensis; **Location:** country: Japan; stateProvince: Iwate; municipality: Hagishou; **Identification:** identifiedBy: Yoko Takata; dateIdentified: 2010; **Event:** year: 2008; month: 10; day: 8; **Record Level:** basisOfRecord: PreservedSpecimen

##### Ecological interactions

###### Conservation status

National: VU (Ministry of the Environment, Japan 2013).

##### Distribution

Japan.

##### Notes

This species usually inhabits lotic environments of river (Kawanabe et al. 2001, Hosoya 2013). However, Sugiyama (1997) mentioned that this species often inhabits isolated ponds in the mountains in Tohoaku region. Our records of this species also include the isolated irrigation ponds as well as the rivers.

#### Oncorhynchus keta

(Walbaum, 1792)

##### Materials

- **Type status:** Other material. **Occurrence:** catalogNumber: KPM-NI 21183; recordedBy: Yusuke Miyazaki; individualCount: 1; **Taxon:** scientificName: Oncorhynchus keta; **Location:** country: Japan; stateProvince: Iwate; locality: Ichinono River; verbatimLatitude: 38°54′19″N; verbatimLongitude: 141°02′41″E; **Identification:** identifiedBy: Yusuke Miyazaki; dateIdentified: 2008; **Event:** year: 2008; month: 5; day: 3; **Record Level:** basisOfRecord: PreservedSpecimen
- **Type status:** Other material. **Occurrence:** catalogNumber: KPM-NI 22429; recordedBy: Muneo Sasaki; individualCount: 1; **Taxon:** scientificName: Oncorhynchus keta; **Location:** country: Japan; stateProvince: Iwate; locality: Kubo River; verbatimLatitude: 38°55′21.2″N; verbatimLongitude: 141°05′25.1″E; **Identification:** identifiedBy: Hiroshi Senou; dateIdentified: 2008; **Event:** year: 2008; month: 10; day: 22; **Record Level:** basisOfRecord: PreservedSpecimen
- **Type status:** Other material. **Occurrence:** catalogNumber: KPM-NI 22430; recordedBy: Muneo Sasaki; individualCount: 1; **Taxon:** scientificName: Oncorhynchus keta; **Location:** country: Japan; stateProvince: Iwate; locality: Kubo River; verbatimLatitude: 38°55′21.2″N; verbatimLongitude: 141°05′25.1″E; **Identification:** identifiedBy: Hiroshi Senou; dateIdentified: 2008; **Event:** year: 2008; month: 10; day: 22; **Record Level:** basisOfRecord: PreservedSpecimen
- **Type status:** Other material. **Occurrence:** catalogNumber: KPM-NR 43909; recordedBy: Yusuke Miyazaki; individualCount: 1; **Taxon:** scientificName: Oncorhynchus keta; **Location:** country: Japan; stateProvince: Iwate; locality: Tochikura River; verbatimLatitude: 38°54′29.3″N; verbatimLongitude: 141°02′52.8″E; **Identification:** identifiedBy: Yusuke Miyazaki; dateIdentified: 2009; **Event:** year: 2008; month: 10; day: 23; **Record Level:** basisOfRecord: Photography
- **Type status:** Other material. **Occurrence:** catalogNumber: KPM-NR 43910; recordedBy: Yusuke Miyazaki; individualCount: 1; **Taxon:** scientificName: Oncorhynchus keta; **Location:** country: Japan; stateProvince: Iwate; locality: Ichinono River; verbatimLatitude: 38°53′53.0″N; verbatimLongitude: 141°01′16.8″E; **Identification:** identifiedBy: Yusuke Miyazaki; dateIdentified: 2009; **Event:** year: 2008; month: 10; day: 23; **Record Level:** basisOfRecord: Photography
- **Type status:** Other material. **Occurrence:** catalogNumber: NSMT-P 92194; recordedBy: Muneo Sasaki; individualCount: 1; **Taxon:** scientificName: Oncorhynchus keta; **Location:** country: Japan; stateProvince: Iwate; locality: Kubo River; verbatimLatitude: 38°55′21.2″N; verbatimLongitude: 141°05′25.1″E; **Identification:** identifiedBy: Masanori Nakae; dateIdentified: 2009; **Event:** year: 2008; month: 10; day: 22; **Record Level:** basisOfRecord: PreservedSpecimen
- **Type status:** Other material. **Occurrence:** catalogNumber: NSMT-P 92195; recordedBy: Muneo Sasaki; individualCount: 1; **Taxon:** scientificName: Oncorhynchus keta; **Location:** country: Japan; stateProvince: Iwate; locality: Kubo River; verbatimLatitude: 38°55′21.2″N; verbatimLongitude: 141°05′25.1″E; **Identification:** identifiedBy: Masanori Nakae; dateIdentified: 2009; **Event:** year: 2008; month: 10; day: 22; **Record Level:** basisOfRecord: PreservedSpecimen

##### Distribution

North Pacific.

##### Notes

This species was recorded from lotic environments in our surveys.

#### Oncorhynchus masou masou

(Brevoort, 1856)

##### Materials

- **Type status:** Other material. **Occurrence:** catalogNumber: KPM-NI 21181; recordedBy: Yusuke Miyazaki; individualCount: 1; **Taxon:** scientificName: Oncorhynchus masou masou; **Location:** country: Japan; stateProvince: Iwate; locality: Ichinono River; verbatimLatitude: 38°54′19″N; verbatimLongitude: 141°02′41″E; **Identification:** identifiedBy: Yusuke Miyazaki; dateIdentified: 2008; **Event:** year: 2008; month: 5; day: 3; **Record Level:** basisOfRecord: PreservedSpecimen
- **Type status:** Other material. **Occurrence:** catalogNumber: KPM-NI 22250; recordedBy: Yusuke Miyazaki; individualCount: 1; **Taxon:** scientificName: Oncorhynchus masou masou; **Location:** country: Japan; stateProvince: Iwate; locality: Ichinono River; verbatimLatitude: 38°54′17.9″N; verbatimLongitude: 141°02′42.1″E; **Identification:** identifiedBy: Yusuke Miyazaki; dateIdentified: 2008; **Event:** year: 2008; month: 7; day: 5; **Record Level:** basisOfRecord: PreservedSpecimen
- **Type status:** Other material. **Occurrence:** catalogNumber: KPM-NI 22251; recordedBy: Yusuke Miyazaki; individualCount: 1; **Taxon:** scientificName: Oncorhynchus masou masou; **Location:** country: Japan; stateProvince: Iwate; locality: Ichinono River; verbatimLatitude: 38°54′17.9″N; verbatimLongitude: 141°02′42.1″E; **Identification:** identifiedBy: Yusuke Miyazaki; dateIdentified: 2008; **Event:** year: 2008; month: 7; day: 5; **Record Level:** basisOfRecord: PreservedSpecimen
- **Type status:** Other material. **Occurrence:** catalogNumber: KPM-NI 22256; recordedBy: Yusuke Miyazaki; individualCount: 1; **Taxon:** scientificName: Oncorhynchus masou masou; **Location:** country: Japan; stateProvince: Iwate; locality: Tochikura River; verbatimLatitude: 38°54′29.3″N; verbatimLongitude: 141°02′52.8″E; **Identification:** identifiedBy: Yusuke Miyazaki; dateIdentified: 2008; **Event:** year: 2008; month: 7; day: 9; **Record Level:** basisOfRecord: PreservedSpecimen
- **Type status:** Other material. **Occurrence:** catalogNumber: KPM-NI 22431; recordedBy: Yusuke Miyazaki; individualCount: 1; **Taxon:** scientificName: Oncorhynchus masou masou; **Location:** country: Japan; stateProvince: Iwate; locality: Ichinono River; verbatimLatitude: 38°53′25.7″N; verbatimLongitude: 141°00′18.0″E; **Identification:** identifiedBy: Yusuke Miyazaki; dateIdentified: 2008; **Event:** year: 2008; month: 7; day: 7; **Record Level:** basisOfRecord: PreservedSpecimen
- **Type status:** Other material. **Occurrence:** catalogNumber: KPM-NI 22432; recordedBy: Yusuke Miyazaki; individualCount: 1; **Taxon:** scientificName: Oncorhynchus masou masou; **Location:** country: Japan; stateProvince: Iwate; locality: Ichinono River; verbatimLatitude: 38°53′25.7″N; verbatimLongitude: 141°00′18.0″E; **Identification:** identifiedBy: Yusuke Miyazaki; dateIdentified: 2008; **Event:** year: 2008; month: 7; day: 7; **Record Level:** basisOfRecord: PreservedSpecimen
- **Type status:** Other material. **Occurrence:** catalogNumber: KPM-NI 23742; recordedBy: Yusuke Miyazaki; individualCount: 1; **Taxon:** scientificName: Oncorhynchus masou masou; **Location:** country: Japan; stateProvince: Iwate; locality: Ichinono River; verbatimLatitude: 38°53′53.0″N; verbatimLongitude: 141°01′16.8″E; **Identification:** identifiedBy: Hiroshi Senou; dateIdentified: 2009; **Event:** year: 2009; month: 5; day: 3; **Record Level:** basisOfRecord: PreservedSpecimen
- **Type status:** Other material. **Occurrence:** catalogNumber: KPM-NI 23991; recordedBy: Yusuke Miyazaki; individualCount: 1; **Taxon:** scientificName: Oncorhynchus masou masou; **Location:** country: Japan; stateProvince: Iwate; locality: Tochikura River; verbatimLatitude: 38°54′59″N; verbatimLongitude: 140°58′51″E; **Identification:** identifiedBy: Yusuke Miyazaki; dateIdentified: 2009; **Event:** year: 2009; month: 4; day: 30; **Record Level:** basisOfRecord: PreservedSpecimen
- **Type status:** Other material. **Occurrence:** catalogNumber: KPM-NI 23992; recordedBy: Yusuke Miyazaki; individualCount: 1; **Taxon:** scientificName: Oncorhynchus masou masou; **Location:** country: Japan; stateProvince: Iwate; locality: Tochikura River; verbatimLatitude: 38°54′59″N; verbatimLongitude: 140°58′51″E; **Identification:** identifiedBy: Yusuke Miyazaki; dateIdentified: 2009; **Event:** year: 2009; month: 4; day: 30; **Record Level:** basisOfRecord: PreservedSpecimen

##### Ecological interactions

###### Conservation status

National: NT (Ministry of the Environment, Japan 2013).

##### Distribution

Japan.

##### Notes

This species was recorded from lotic environments in our surveys.

#### Oryzias latipes

(Temminck & Schlegel, 1846)

##### Materials

- **Type status:** Other material. **Occurrence:** catalogNumber: KPM-NI 19454; recordedBy: Yusuke Miyazaki; individualCount: 1; **Taxon:** scientificName: Oryzias latipes; **Location:** country: Japan; stateProvince: Iwate; locality: irrigation pond of the Kubo River Basin; verbatimLatitude: 38°55′42″N; verbatimLongitude: 141°02′06″E; **Identification:** identifiedBy: Hiroshi Senou; dateIdentified: 2007; **Event:** year: 2007; month: 9; day: 11; **Record Level:** basisOfRecord: PreservedSpecimen
- **Type status:** Other material. **Occurrence:** catalogNumber: KPM-NI 21201; recordedBy: Yusuke Miyazaki; individualCount: 1; **Taxon:** scientificName: Oryzias latipes; **Location:** country: Japan; stateProvince: Iwate; locality: irrigation pond of the Kubo River Basin; verbatimLatitude: 38°56′12″N; verbatimLongitude: 141°01′34″E; **Identification:** identifiedBy: Yusuke Miyazaki; dateIdentified: 2008; **Event:** year: 2008; month: 5; day: 6; **Record Level:** basisOfRecord: PreservedSpecimen
- **Type status:** Other material. **Occurrence:** catalogNumber: KPM-NI 21202; recordedBy: Yusuke Miyazaki; individualCount: 1; **Taxon:** scientificName: Oryzias latipes; **Location:** country: Japan; stateProvince: Iwate; locality: irrigation pond of the Kubo River Basin; verbatimLatitude: 38°56′12″N; verbatimLongitude: 141°01′34″E; **Identification:** identifiedBy: Yusuke Miyazaki; dateIdentified: 2008; **Event:** year: 2008; month: 5; day: 6; **Record Level:** basisOfRecord: PreservedSpecimen
- **Type status:** Other material. **Occurrence:** catalogNumber: KPM-NI 21203; recordedBy: Yusuke Miyazaki; individualCount: 1; **Taxon:** scientificName: Oryzias latipes; **Location:** country: Japan; stateProvince: Iwate; locality: irrigation pond of the Kubo River Basin; verbatimLatitude: 38°56′12″N; verbatimLongitude: 141°01′34″E; **Identification:** identifiedBy: Yusuke Miyazaki; dateIdentified: 2008; **Event:** year: 2008; month: 5; day: 6; **Record Level:** basisOfRecord: PreservedSpecimen
- **Type status:** Other material. **Occurrence:** catalogNumber: KPM-NI 21400; recordedBy: Hayato Takeda; individualCount: 1; **Taxon:** scientificName: Oryzias latipes; **Location:** country: Japan; stateProvince: Iwate; locality: channel of rice paddy, Kubo River Basin; verbatimLatitude: 38°55′50″N; verbatimLongitude: 141°03′32″E; **Identification:** identifiedBy: Yusuke Miyazaki; dateIdentified: 2008; **Event:** year: 2008; month: 6; day: 7; **Record Level:** basisOfRecord: PreservedSpecimen
- **Type status:** Other material. **Occurrence:** catalogNumber: KPM-NI 21408; recordedBy: Hayato Takeda; individualCount: 1; **Taxon:** scientificName: Oryzias latipes; **Location:** country: Japan; stateProvince: Iwate; locality: channel of rice paddy, Kubo River Basin; verbatimLatitude: 38°55′49″N; verbatimLongitude: 141°03′33″E; **Identification:** identifiedBy: Yusuke Miyazaki; dateIdentified: 2008; **Event:** year: 2008; month: 6; day: 7; **Record Level:** basisOfRecord: PreservedSpecimen
- **Type status:** Other material. **Occurrence:** catalogNumber: KPM-NI 21409; recordedBy: Hayato Takeda; individualCount: 1; **Taxon:** scientificName: Oryzias latipes; **Location:** country: Japan; stateProvince: Iwate; locality: channel of rice paddy, Kubo River Basin; verbatimLatitude: 38°55′49″N; verbatimLongitude: 141°03′33″E; **Identification:** identifiedBy: Yusuke Miyazaki; dateIdentified: 2008; **Event:** year: 2008; month: 6; day: 7; **Record Level:** basisOfRecord: PreservedSpecimen
- **Type status:** Other material. **Occurrence:** catalogNumber: KPM-NI 21411; recordedBy: Hayato Takeda; individualCount: 1; **Taxon:** scientificName: Oryzias latipes; **Location:** country: Japan; stateProvince: Iwate; locality: channel of rice paddy, Kubo River Basin; verbatimLatitude: 38°55′49″N; verbatimLongitude: 141°03′33″E; **Identification:** identifiedBy: Yusuke Miyazaki; dateIdentified: 2008; **Event:** year: 2008; month: 6; day: 7; **Record Level:** basisOfRecord: PreservedSpecimen
- **Type status:** Other material. **Occurrence:** catalogNumber: KPM-NI 21412; recordedBy: Hayato Takeda; individualCount: 1; **Taxon:** scientificName: Oryzias latipes; **Location:** country: Japan; stateProvince: Iwate; locality: channel of rice paddy, Kubo River Basin; verbatimLatitude: 38°55′49″N; verbatimLongitude: 141°03′33″E; **Identification:** identifiedBy: Yusuke Miyazaki; dateIdentified: 2008; **Event:** year: 2008; month: 6; day: 7; **Record Level:** basisOfRecord: PreservedSpecimen
- **Type status:** Other material. **Occurrence:** catalogNumber: KPM-NI 22286; recordedBy: Yusuke Miyazaki; individualCount: 1; **Taxon:** scientificName: Oryzias latipes; **Location:** country: Japan; stateProvince: Iwate; locality: irrigation pond of the Kubo River Basin; verbatimLatitude: 38°55′41.3″N; verbatimLongitude: 141°02′06.3″E; **Identification:** identifiedBy: Yusuke Miyazaki; dateIdentified: 2008; **Event:** year: 2008; month: 8; day: 23; **Record Level:** basisOfRecord: PreservedSpecimen
- **Type status:** Other material. **Occurrence:** catalogNumber: KPM-NI 23979; recordedBy: Yusuke Miyazaki; individualCount: 1; **Taxon:** scientificName: Oryzias latipes; **Location:** country: Japan; stateProvince: Iwate; locality: irrigation pond of the Kubo River Basin; verbatimLatitude: 38°55′21″N; verbatimLongitude: 141°03′30″E; **Identification:** identifiedBy: Yusuke Miyazaki; dateIdentified: 2009; **Event:** year: 2009; month: 5; day: 18; **Record Level:** basisOfRecord: PreservedSpecimen
- **Type status:** Other material. **Occurrence:** catalogNumber: KPM-NI 23980; recordedBy: Yusuke Miyazaki; individualCount: 1; **Taxon:** scientificName: Oryzias latipes; **Location:** country: Japan; stateProvince: Iwate; locality: irrigation pond of the Kubo River Basin; verbatimLatitude: 38°55′26″N; verbatimLongitude: 141°04′06″E; **Identification:** identifiedBy: Yusuke Miyazaki; dateIdentified: 2009; **Event:** year: 2009; month: 5; day: 18; **Record Level:** basisOfRecord: PreservedSpecimen
- **Type status:** Other material. **Occurrence:** catalogNumber: KPM-NI 23981; recordedBy: Yusuke Miyazaki; individualCount: 1; **Taxon:** scientificName: Oryzias latipes; **Location:** country: Japan; stateProvince: Iwate; locality: irrigation pond of the Kubo River Basin; verbatimLatitude: 38°55′14″N; verbatimLongitude: 141°01′13″E; **Identification:** identifiedBy: Yusuke Miyazaki; dateIdentified: 2009; **Event:** year: 2009; month: 5; day: 15; **Record Level:** basisOfRecord: PreservedSpecimen
- **Type status:** Other material. **Occurrence:** catalogNumber: KPM-NI 24421; recordedBy: Yusuke Miyazaki; individualCount: 3; **Taxon:** scientificName: Oryzias latipes; **Location:** country: Japan; stateProvince: Iwate; municipality: Dougasawa, Hagishou; verbatimLatitude: 38°56′09″N; verbatimLongitude: 141°01′34″E; **Identification:** identifiedBy: Yusuke Miyazaki; dateIdentified: 2009; **Event:** year: 2009; month: 9; day: 20; **Record Level:** basisOfRecord: PreservedSpecimen
- **Type status:** Other material. **Occurrence:** catalogNumber: KPM-NI 24989; recordedBy: Yusuke Miyazaki; individualCount: 1; **Taxon:** scientificName: Oryzias latipes; **Location:** country: Japan; stateProvince: Iwate; locality: irrigation pond of the Ichinono River Basin; verbatimLatitude: 38°54′11″N; verbatimLongitude: 140°59′54″E; **Identification:** identifiedBy: Hiroshi Senou; dateIdentified: 2013; **Event:** year: 2008; month: 10; day: 9; **Record Level:** basisOfRecord: PreservedSpecimen
- **Type status:** Other material. **Occurrence:** catalogNumber: KPM-NI 24992; recordedBy: Yusuke Miyazaki; individualCount: 2; **Taxon:** scientificName: Oryzias latipes; **Location:** country: Japan; stateProvince: Iwate; locality: irrigation pond of the Tochikura River Basin; verbatimLatitude: 38°54′32″N; verbatimLongitude: 141°00′44″E; **Identification:** identifiedBy: Yusuke Miyazaki; dateIdentified: 2009; **Event:** year: 2008; month: 10; day: 9; **Record Level:** basisOfRecord: PreservedSpecimen
- **Type status:** Other material. **Occurrence:** catalogNumber: NSMT-P 90721; recordedBy: Yusuke Miyazaki and Yoko Takata; individualCount: 2; **Taxon:** scientificName: Oryzias latipes; **Location:** country: Japan; stateProvince: Iwate; municipality: Hagishou; verbatimLatitude: 38°54′32″N; verbatimLongitude: 141°00′45″E; **Identification:** identifiedBy: Yusuke Miyazaki; dateIdentified: 2008; **Event:** year: 2008; month: 5; day: 3; **Record Level:** basisOfRecord: PreservedSpecimen
- **Type status:** Other material. **Occurrence:** catalogNumber: NSMT-P 96851; recordedBy: Yusuke Miyazaki and Yoko Takata; individualCount: 1; **Taxon:** scientificName: Oryzias latipes; **Location:** country: Japan; stateProvince: Iwate; locality: irrigation pond of the Kubo River Basin; **Identification:** identifiedBy: Kaoru Kuriiwa; dateIdentified: 2010; **Event:** year: 2008; month: 10; day: 5; **Record Level:** basisOfRecord: PreservedSpecimen
- **Type status:** Other material. **Occurrence:** catalogNumber: NSMT-P 96852; recordedBy: Yusuke Miyazaki and Yoko Takata; individualCount: 2; **Taxon:** scientificName: Oryzias latipes; **Location:** country: Japan; stateProvince: Iwate; locality: irrigation pond of the Kubo River Basin; **Identification:** identifiedBy: Kaoru Kuriiwa; dateIdentified: 2010; **Event:** year: 2008; month: 10; day: 5; **Record Level:** basisOfRecord: PreservedSpecimen
- **Type status:** Other material. **Occurrence:** catalogNumber: NSMT-P 97084; recordedBy: Yusuke Miyazaki and Yoko Takata; individualCount: 26; **Taxon:** scientificName: Oryzias latipes; **Location:** country: Japan; stateProvince: Iwate; locality: irrigation pond of the Kubo River Basin; **Identification:** identifiedBy: Masanori Nakae; dateIdentified: 2010; **Event:** year: 2008; month: 10; day: 10; **Record Level:** basisOfRecord: PreservedSpecimen
- **Type status:** Other material. **Occurrence:** catalogNumber: NSMT-P 97110; recordedBy: Yusuke Miyazaki and Yoko Takata; individualCount: 1; **Taxon:** scientificName: Oryzias latipes; **Location:** country: Japan; stateProvince: Iwate; locality: irrigation pond of the Kubo River Basin; **Identification:** identifiedBy: Masanori Nakae; dateIdentified: 2010; **Event:** year: 2008; month: 10; day: 8; **Record Level:** basisOfRecord: PreservedSpecimen

##### Ecological interactions

###### Native status

Non-native (See Discussion)

###### Conservation status

National: VU (Ministry of the Environment, Japan 2013); Prefectural: VU (Iwate Prefecture 2014).

##### Distribution

Japan.

##### Notes

The species was recorded only from irrigation ponds with rich aquatic plants in the study.

#### Micropterus salmoides

(Lacepède, 1802)

##### Materials

- **Type status:** Other material. **Occurrence:** catalogNumber: KPM-NI 21182; recordedBy: Yusuke Miyazaki; individualCount: 1; **Taxon:** scientificName: Micropterus salmoides; **Location:** country: Japan; stateProvince: Iwate; locality: irrigation pond of the Kubo River Basin; verbatimLatitude: 38°55′23″N; verbatimLongitude: 141°02′57″E; **Identification:** identifiedBy: Yusuke Miyazaki; dateIdentified: 2008; **Event:** year: 2008; month: 4; day: 30; **Record Level:** basisOfRecord: PreservedSpecimen
- **Type status:** Other material. **Occurrence:** catalogNumber: KPM-NI 23743; recordedBy: Yusuke Miyazaki; individualCount: 1; **Taxon:** scientificName: Micropterus salmoides; **Location:** country: Japan; stateProvince: Iwate; locality: irrigation pond of the Kubo River Basin; verbatimLatitude: 38°55′23″N; verbatimLongitude: 141°02′57″E; **Identification:** identifiedBy: Hiroshi Senou; dateIdentified: 2009; **Event:** year: 2009; month: 5; day: 16; **Record Level:** basisOfRecord: PreservedSpecimen
- **Type status:** Other material. **Occurrence:** catalogNumber: KPM-NI 24471; recordedBy: Yusuke Miyazaki; individualCount: 1; **Taxon:** scientificName: Micropterus salmoides; **Location:** country: Japan; stateProvince: Iwate; locality: irrigation pond of the Kubo River Basin; verbatimLatitude: 38°55′12″N; verbatimLongitude: 141°00′12″E; **Identification:** identifiedBy: Yusuke Miyazaki; dateIdentified: 2009; **Event:** year: 2009; month: 8; day: 5; **Record Level:** basisOfRecord: PreservedSpecimen
- **Type status:** Other material. **Occurrence:** catalogNumber: KPM-NI 24472; recordedBy: Yusuke Miyazaki; individualCount: 1; **Taxon:** scientificName: Micropterus salmoides; **Location:** country: Japan; stateProvince: Iwate; locality: irrigation pond of the Ichinono River Basin; verbatimLatitude: 38°54′10.1″N; verbatimLongitude: 141°01′16.8″E; **Identification:** identifiedBy: Yusuke Miyazaki; dateIdentified: 2009; **Event:** year: 2009; month: 8; day: 5; **Record Level:** basisOfRecord: PreservedSpecimen
- **Type status:** Other material. **Occurrence:** catalogNumber: NSMT-P 96893; recordedBy: Yusuke Miyazaki; individualCount: 1; **Taxon:** scientificName: Micropterus salmoides; **Location:** country: Japan; stateProvince: Iwate; locality: irrigation pond of the Kubo River Basin; **Identification:** identifiedBy: Yoko Takata; dateIdentified: 2010; **Event:** year: 2008; month: 10; day: 10; **Record Level:** basisOfRecord: PreservedSpecimen
- **Type status:** Other material. **Occurrence:** catalogNumber: NSMT-P 96894; recordedBy: Yusuke Miyazaki; individualCount: 1; **Taxon:** scientificName: Micropterus salmoides; **Location:** country: Japan; stateProvince: Iwate; locality: irrigation pond of the Kubo River Basin; **Identification:** identifiedBy: Yoko Takata; dateIdentified: 2010; **Event:** year: 2008; month: 10; day: 10; **Record Level:** basisOfRecord: PreservedSpecimen
- **Type status:** Other material. **Occurrence:** catalogNumber: NSMT-P 96902; recordedBy: Yusuke Miyazaki; individualCount: 3; **Taxon:** scientificName: Micropterus salmoides; **Location:** country: Japan; stateProvince: Iwate; locality: irrigation pond of the Kubo River Basin; **Identification:** identifiedBy: Yoko Takata; dateIdentified: 2010; **Event:** year: 2008; month: 10; day: 8; **Record Level:** basisOfRecord: PreservedSpecimen

##### Ecological interactions

###### Native status

Non-native (100 of the World's and Japanese Worst Invasive Alien Species: Lowe et al. 2000, Murakami and Washitani 2002).

##### Distribution

North America.

##### Notes

This non-native invasive species was recorded only from irrigation ponds on our surveys.

#### Cottus nozawae

Snyder, 1911

##### Materials

- **Type status:** Other material. **Occurrence:** catalogNumber: KPM-NI 24408; recordedBy: Yusuke Miyazaki, Shogo Nishihara and Taichi Okeda; individualCount: 1; **Taxon:** scientificName: Cottus nozawae; **Location:** country: Japan; stateProvince: Iwate; locality: Kubo River; **Identification:** identifiedBy: Hiroshi Senou; dateIdentified: 2009; **Event:** year: 2009; month: 9; day: 23; **Record Level:** basisOfRecord: PreservedSpecimen
- **Type status:** Other material. **Occurrence:** catalogNumber: KPM-NI 24409; recordedBy: Yusuke Miyazaki, Shogo Nishihara and Taichi Okeda; individualCount: 1; **Taxon:** scientificName: Cottus nozawae; **Location:** country: Japan; stateProvince: Iwate; locality: Kubo River; **Identification:** identifiedBy: Hiroshi Senou; dateIdentified: 2009; **Event:** year: 2009; month: 9; day: 23; **Record Level:** basisOfRecord: PreservedSpecimen
- **Type status:** Other material. **Occurrence:** catalogNumber: KPM-NI 24410; recordedBy: Yusuke Miyazaki, Shogo Nishihara and Taichi Okeda; individualCount: 1; **Taxon:** scientificName: Cottus nozawae; **Location:** country: Japan; stateProvince: Iwate; locality: Kubo River; **Identification:** identifiedBy: Hiroshi Senou; dateIdentified: 2009; **Event:** year: 2009; month: 9; day: 23; **Record Level:** basisOfRecord: PreservedSpecimen
- **Type status:** Other material. **Occurrence:** catalogNumber: KPM-NI 24411; recordedBy: Yusuke Miyazaki, Shogo Nishihara and Taichi Okeda; individualCount: 1; **Taxon:** scientificName: Cottus nozawae; **Location:** country: Japan; stateProvince: Iwate; locality: Kubo River; **Identification:** identifiedBy: Hiroshi Senou; dateIdentified: 2009; **Event:** year: 2009; month: 9; day: 23; **Record Level:** basisOfRecord: PreservedSpecimen

##### Ecological interactions

###### Conservation status

National: LP (Ministry of the Environment, Japan 2013); Prefectural: VU (Iwate Prefecture 2014).

##### Distribution

Japan.

##### Notes

This species usually inhabitsfast lotic waters (Kawanabe et al. 2001), but, our collections of this species were from slow-moving back waters of the upper reaches of the Kubo River.

#### Rhinogobius brunneus

(Temminck & Schlegel, 1845)

##### Materials

- **Type status:** Other material. **Occurrence:** catalogNumber: KPM-NI 21192; recordedBy: Hiroshi Senou; individualCount: 1; **Taxon:** scientificName: Rhinogobius brunneus; **Location:** country: Japan; stateProvince: Iwate; locality: Tochikura River; verbatimLatitude: 38°54′43.05″N; verbatimLongitude: 141°01′24.39″E; **Identification:** identifiedBy: Hiroshi Senou; dateIdentified: 2008; **Event:** year: 2008; month: 5; day: 2; **Record Level:** basisOfRecord: PreservedSpecimen
- **Type status:** Other material. **Occurrence:** catalogNumber: KPM-NI 21193; recordedBy: Hiroshi Senou; individualCount: 1; **Taxon:** scientificName: Rhinogobius brunneus; **Location:** country: Japan; stateProvince: Iwate; locality: Tochikura River; verbatimLatitude: 38°54′43.05″N; verbatimLongitude: 141°01′24.39″E; **Identification:** identifiedBy: Hiroshi Senou; dateIdentified: 2008; **Event:** year: 2008; month: 5; day: 2; **Record Level:** basisOfRecord: PreservedSpecimen
- **Type status:** Other material. **Occurrence:** catalogNumber: KPM-NI 21218; recordedBy: Hiroshi Senou and Takumi Senou; individualCount: 5; **Taxon:** scientificName: Rhinogobius brunneus; **Location:** country: Japan; stateProvince: Iwate; locality: irrigation pond of the Kubo River Basin; verbatimLatitude: 38°56′34.29″N; verbatimLongitude: 141°00′42.59″E; **Identification:** identifiedBy: Hiroshi Senou; dateIdentified: 2008; **Event:** year: 2008; month: 5; day: 2; **Record Level:** basisOfRecord: PreservedSpecimen
- **Type status:** Other material. **Occurrence:** catalogNumber: KPM-NI 21401; recordedBy: Hayato Takeda; individualCount: 1; **Taxon:** scientificName: Rhinogobius brunneus; **Location:** country: Japan; stateProvince: Iwate; locality: channel of rice paddy, Kubo River Basin; verbatimLatitude: 38°55′49″N; verbatimLongitude: 141°03′33″E; **Identification:** identifiedBy: Yusuke Miyazaki; dateIdentified: 2008; **Event:** year: 2008; month: 6; day: 7; **Record Level:** basisOfRecord: PreservedSpecimen
- **Type status:** Other material. **Occurrence:** catalogNumber: KPM-NI 21410; recordedBy: Yusuke Miyazaki; individualCount: 1; **Taxon:** scientificName: Rhinogobius brunneus; **Location:** country: Japan; stateProvince: Iwate; locality: irrigation pond of the Tochikura River Basin; verbatimLatitude: 38°54′35″N; verbatimLongitude: 141°01′07″E; **Identification:** identifiedBy: Yusuke Miyazaki; dateIdentified: 2008; **Event:** year: 2008; month: 6; day: 8; **Record Level:** basisOfRecord: PreservedSpecimen
- **Type status:** Other material. **Occurrence:** catalogNumber: KPM-NI 22290; recordedBy: Yusuke Miyazaki; individualCount: 1; **Taxon:** scientificName: Rhinogobius brunneus; **Location:** country: Japan; stateProvince: Iwate; locality: irrigation pond of the Ichinono River Basin; verbatimLatitude: 38°54′10.1″N; verbatimLongitude: 141°01′16.8″E; **Identification:** identifiedBy: Yusuke Miyazaki; dateIdentified: 2008; **Event:** year: 2008; month: 9; day: 16; **Record Level:** basisOfRecord: PreservedSpecimen
- **Type status:** Other material. **Occurrence:** catalogNumber: KPM-NI 22275; recordedBy: Yusuke Miyazaki; individualCount: 1; **Taxon:** scientificName: Rhinogobius brunneus; **Location:** country: Japan; stateProvince: Iwate; locality: irrigation pond of the Tochikura River Basin; verbatimLatitude: 38°54′26″N; verbatimLongitude: 141°00′52″E; **Identification:** identifiedBy: Yusuke Miyazaki; dateIdentified: 2008; **Event:** year: 2008; month: 9; day: 13; **Record Level:** basisOfRecord: PreservedSpecimen
- **Type status:** Other material. **Occurrence:** catalogNumber: KPM-NI 22302; recordedBy: Yusuke Miyazaki; individualCount: 1; **Taxon:** scientificName: Rhinogobius brunneus; **Location:** country: Japan; stateProvince: Iwate; locality: irrigation pond of the Tochikura River Basin; verbatimLatitude: 38°54′36″N; verbatimLongitude: 141°01′07″E; **Identification:** identifiedBy: Yusuke Miyazaki; dateIdentified: 2008; **Event:** year: 2008; month: 9; day: 15; **Record Level:** basisOfRecord: PreservedSpecimen
- **Type status:** Other material. **Occurrence:** catalogNumber: KPM-NI 22267; recordedBy: Yusuke Miyazaki; individualCount: 1; **Taxon:** scientificName: Rhinogobius brunneus; **Location:** country: Japan; stateProvince: Iwate; locality: irrigation pond of the Kubo River Basin; verbatimLatitude: 38°56′35″N; verbatimLongitude: 141°00′42.9″E; **Identification:** identifiedBy: Yusuke Miyazaki; dateIdentified: 2008; **Event:** year: 2008; month: 8; day: 26; **Record Level:** basisOfRecord: PreservedSpecimen
- **Type status:** Other material. **Occurrence:** catalogNumber: KPM-NI 22270; recordedBy: Yusuke Miyazaki; individualCount: 1; **Taxon:** scientificName: Rhinogobius brunneus; **Location:** country: Japan; stateProvince: Iwate; locality: irrigation pond of the Kubo River Basin; verbatimLatitude: 38°56′33.1″N; verbatimLongitude: 141°00′46.1″E; **Identification:** identifiedBy: Yusuke Miyazaki; dateIdentified: 2008; **Event:** year: 2008; month: 8; day: 26; **Record Level:** basisOfRecord: PreservedSpecimen
- **Type status:** Other material. **Occurrence:** catalogNumber: KPM-NI 22293; recordedBy: Yusuke Miyazaki; individualCount: 1; **Taxon:** scientificName: Rhinogobius brunneus; **Location:** country: Japan; stateProvince: Iwate; locality: irrigation pond of the Kubo River Basin; verbatimLatitude: 38°55′46.1″N; verbatimLongitude: 140°53′16.2″E; **Identification:** identifiedBy: Yusuke Miyazaki; dateIdentified: 2008; **Event:** year: 2008; month: 8; day: 25; **Record Level:** basisOfRecord: PreservedSpecimen
- **Type status:** Other material. **Occurrence:** catalogNumber: KPM-NI 22283; recordedBy: Yusuke Miyazaki; individualCount: 1; **Taxon:** scientificName: Rhinogobius brunneus; **Location:** country: Japan; stateProvince: Iwate; locality: irrigation pond of the Kubo River Basin; verbatimLatitude: 38°55′19.4″N; verbatimLongitude: 140°59′29.1″E; **Identification:** identifiedBy: Yusuke Miyazaki; dateIdentified: 2008; **Event:** year: 2008; month: 8; day: 27; **Record Level:** basisOfRecord: PreservedSpecimen
- **Type status:** Other material. **Occurrence:** catalogNumber: KPM-NI 22279; recordedBy: Yusuke Miyazaki; individualCount: 9; **Taxon:** scientificName: Rhinogobius brunneus; **Location:** country: Japan; stateProvince: Iwate; locality: irrigation pond of the Tochikura River Basin; verbatimLatitude: 38°54′25.7″N; verbatimLongitude: 141°02′25.8″E; **Identification:** identifiedBy: Yusuke Miyazaki; dateIdentified: 2008; **Event:** year: 2008; month: 9; day: 15; **Record Level:** basisOfRecord: PreservedSpecimen
- **Type status:** Other material. **Occurrence:** catalogNumber: KPM-NI 22289; recordedBy: Yusuke Miyazaki; individualCount: 1; **Taxon:** scientificName: Rhinogobius brunneus; **Location:** country: Japan; stateProvince: Iwate; locality: irrigation pond of the Tochikura River Basin; verbatimLatitude: 38°54′56.9″N; verbatimLongitude: 141°01′51.9″E; **Identification:** identifiedBy: Yusuke Miyazaki; dateIdentified: 2008; **Event:** year: 2008; month: 9; day: 18; **Record Level:** basisOfRecord: PreservedSpecimen
- **Type status:** Other material. **Occurrence:** catalogNumber: KPM-NI 22298; recordedBy: Yusuke Miyazaki; individualCount: 1; **Taxon:** scientificName: Rhinogobius brunneus; **Location:** country: Japan; stateProvince: Iwate; locality: irrigation pond of the Tochikura River Basin; verbatimLatitude: 38°54′25.7″N; verbatimLongitude: 141°02′14.6″E; **Identification:** identifiedBy: Yusuke Miyazaki; dateIdentified: 2008; **Event:** year: 2008; month: 9; day: 15; **Record Level:** basisOfRecord: PreservedSpecimen
- **Type status:** Other material. **Occurrence:** catalogNumber: KPM-NI 22287; recordedBy: Yusuke Miyazaki; individualCount: 1; **Taxon:** scientificName: Rhinogobius brunneus; **Location:** country: Japan; stateProvince: Iwate; locality: irrigation pond of the Kubo River Basin; verbatimLatitude: 38°55′20.2″N; verbatimLongitude: 140°59′35.7″E; **Identification:** identifiedBy: Yusuke Miyazaki; dateIdentified: 2008; **Event:** year: 2008; month: 8; day: 28; **Record Level:** basisOfRecord: PreservedSpecimen
- **Type status:** Other material. **Occurrence:** catalogNumber: KPM-NI 22291; recordedBy: Yusuke Miyazaki; individualCount: 1; **Taxon:** scientificName: Rhinogobius brunneus; **Location:** country: Japan; stateProvince: Iwate; locality: irrigation pond of the Kubo River Basin; verbatimLatitude: 38°55′49″N; verbatimLongitude: 141°01′35″E; **Identification:** identifiedBy: Yusuke Miyazaki; dateIdentified: 2008; **Event:** year: 2008; month: 8; day: 30; **Record Level:** basisOfRecord: PreservedSpecimen
- **Type status:** Other material. **Occurrence:** catalogNumber: KPM-NI 23933; recordedBy: Yusuke Miyazaki; individualCount: 1; **Taxon:** scientificName: Rhinogobius brunneus; **Location:** country: Japan; stateProvince: Iwate; locality: irrigation pond of the Tochikura River Basin; verbatimLatitude: 38°54′44″N; verbatimLongitude: 140°59′54″E; **Identification:** identifiedBy: Yusuke Miyazaki; dateIdentified: 2009; **Event:** year: 2009; month: 5; day: 19; **Record Level:** basisOfRecord: PreservedSpecimen
- **Type status:** Other material. **Occurrence:** catalogNumber: KPM-NI 23934; recordedBy: Yusuke Miyazaki; individualCount: 1; **Taxon:** scientificName: Rhinogobius brunneus; **Location:** country: Japan; stateProvince: Iwate; locality: irrigation pond of the Kubo River Basin; verbatimLatitude: 38°55′25″N; verbatimLongitude: 140°59′46″E; **Identification:** identifiedBy: Yusuke Miyazaki; dateIdentified: 2009; **Event:** year: 2009; month: 4; day: 30; **Record Level:** basisOfRecord: PreservedSpecimen
- **Type status:** Other material. **Occurrence:** catalogNumber: KPM-NI 23935; recordedBy: Yusuke Miyazaki; individualCount: 1; **Taxon:** scientificName: Rhinogobius brunneus; **Location:** country: Japan; stateProvince: Iwate; locality: irrigation pond of the Tochikura River Basin; verbatimLatitude: 38°54′23″N; verbatimLongitude: 141°00′48″E; **Identification:** identifiedBy: Yusuke Miyazaki; dateIdentified: 2009; **Event:** year: 2009; month: 5; day: 20; **Record Level:** basisOfRecord: PreservedSpecimen
- **Type status:** Other material. **Occurrence:** catalogNumber: KPM-NI 23936; recordedBy: Yusuke Miyazaki; individualCount: 1; **Taxon:** scientificName: Rhinogobius brunneus; **Location:** country: Japan; stateProvince: Iwate; locality: irrigation pond of the Kubo River Basin; verbatimLatitude: 38°55′22″N; verbatimLongitude: 140°59′46″E; **Identification:** identifiedBy: Yusuke Miyazaki; dateIdentified: 2009; **Event:** year: 2009; month: 4; day: 30; **Record Level:** basisOfRecord: PreservedSpecimen
- **Type status:** Other material. **Occurrence:** catalogNumber: KPM-NI 23937; recordedBy: Yusuke Miyazaki; individualCount: 1; **Taxon:** scientificName: Rhinogobius brunneus; **Location:** country: Japan; stateProvince: Iwate; locality: irrigation pond of the Kubo River Basin; verbatimLatitude: 38°55′25″N; verbatimLongitude: 141°04′05″E; **Identification:** identifiedBy: Yusuke Miyazaki; dateIdentified: 2009; **Event:** year: 2009; month: 5; day: 18; **Record Level:** basisOfRecord: PreservedSpecimen
- **Type status:** Other material. **Occurrence:** catalogNumber: KPM-NI 23938; recordedBy: Yusuke Miyazaki; individualCount: 1; **Taxon:** scientificName: Rhinogobius brunneus; **Location:** country: Japan; stateProvince: Iwate; locality: irrigation pond of the Kubo River Basin; verbatimLatitude: 38°56′20″N; verbatimLongitude: 141°01′06″E; **Identification:** identifiedBy: Yusuke Miyazaki; dateIdentified: 2009; **Event:** year: 2009; month: 5; day: 2; **Record Level:** basisOfRecord: PreservedSpecimen
- **Type status:** Other material. **Occurrence:** catalogNumber: KPM-NI 23939; recordedBy: Yusuke Miyazaki; individualCount: 1; **Taxon:** scientificName: Rhinogobius brunneus; **Location:** country: Japan; stateProvince: Iwate; locality: irrigation pond of the Tochikura River Basin; verbatimLatitude: 38°54′20″N; verbatimLongitude: 141°00′56″E; **Identification:** identifiedBy: Yusuke Miyazaki; dateIdentified: 2009; **Event:** year: 2009; month: 5; day: 19; **Record Level:** basisOfRecord: PreservedSpecimen
- **Type status:** Other material. **Occurrence:** catalogNumber: KPM-NI 23940; recordedBy: Yusuke Miyazaki; individualCount: 1; **Taxon:** scientificName: Rhinogobius brunneus; **Location:** country: Japan; stateProvince: Iwate; locality: irrigation pond of the Kubo River Basin; verbatimLatitude: 38°56′45″N; verbatimLongitude: 141°00′08″E; **Identification:** identifiedBy: Yusuke Miyazaki; dateIdentified: 2009; **Event:** year: 2009; month: 5; day: 2; **Record Level:** basisOfRecord: PreservedSpecimen
- **Type status:** Other material. **Occurrence:** catalogNumber: KPM-NI 23941; recordedBy: Yusuke Miyazaki; individualCount: 1; **Taxon:** scientificName: Rhinogobius brunneus; **Location:** country: Japan; stateProvince: Iwate; locality: irrigation pond of the Tochikura River Basin; verbatimLatitude: 38°54′22″N; verbatimLongitude: 141°00′45″E; **Identification:** identifiedBy: Yusuke Miyazaki; dateIdentified: 2009; **Event:** year: 2009; month: 5; day: 20; **Record Level:** basisOfRecord: PreservedSpecimen
- **Type status:** Other material. **Occurrence:** catalogNumber: KPM-NI 23942; recordedBy: Yusuke Miyazaki; individualCount: 1; **Taxon:** scientificName: Rhinogobius brunneus; **Location:** country: Japan; stateProvince: Iwate; locality: irrigation pond of the Kubo River Basin; verbatimLatitude: 38°55′08″N; verbatimLongitude: 141°01′05″E; **Identification:** identifiedBy: Yusuke Miyazaki; dateIdentified: 2009; **Event:** year: 2009; month: 5; day: 15; **Record Level:** basisOfRecord: PreservedSpecimen
- **Type status:** Other material. **Occurrence:** catalogNumber: KPM-NI 23943; recordedBy: Yusuke Miyazaki; individualCount: 1; **Taxon:** scientificName: Rhinogobius brunneus; **Location:** country: Japan; stateProvince: Iwate; locality: irrigation pond of the Kubo River Basin; verbatimLatitude: 38°55′13″N; verbatimLongitude: 141°01′10″E; **Identification:** identifiedBy: Yusuke Miyazaki; dateIdentified: 2009; **Event:** year: 2009; month: 5; day: 19; **Record Level:** basisOfRecord: PreservedSpecimen
- **Type status:** Other material. **Occurrence:** catalogNumber: KPM-NI 23944; recordedBy: Yusuke Miyazaki; individualCount: 1; **Taxon:** scientificName: Rhinogobius brunneus; **Location:** country: Japan; stateProvince: Iwate; locality: irrigation pond of the Kubo River Basin; verbatimLatitude: 38°56′38″N; verbatimLongitude: 141°00′48″E; **Identification:** identifiedBy: Yusuke Miyazaki; dateIdentified: 2009; **Event:** year: 2009; month: 5; day: 2; **Record Level:** basisOfRecord: PreservedSpecimen
- **Type status:** Other material. **Occurrence:** catalogNumber: KPM-NI 23945; recordedBy: Yusuke Miyazaki; individualCount: 1; **Taxon:** scientificName: Rhinogobius brunneus; **Location:** country: Japan; stateProvince: Iwate; locality: irrigation pond of the Kubo River Basin; verbatimLatitude: 38°56′38″N; verbatimLongitude: 141°00′48″E; **Identification:** identifiedBy: Yusuke Miyazaki; dateIdentified: 2009; **Event:** year: 2009; month: 5; day: 15; **Record Level:** basisOfRecord: PreservedSpecimen
- **Type status:** Other material. **Occurrence:** catalogNumber: KPM-NI 23946; recordedBy: Yusuke Miyazaki; individualCount: 2; **Taxon:** scientificName: Rhinogobius brunneus; **Location:** country: Japan; stateProvince: Iwate; locality: irrigation pond of the Kubo River Basin; verbatimLatitude: 38°55′09″N; verbatimLongitude: 141°01′10″E; **Identification:** identifiedBy: Yusuke Miyazaki; dateIdentified: 2009; **Event:** year: 2009; month: 5; day: 15; **Record Level:** basisOfRecord: PreservedSpecimen
- **Type status:** Other material. **Occurrence:** catalogNumber: KPM-NI 23947; recordedBy: Yusuke Miyazaki; individualCount: 1; **Taxon:** scientificName: Rhinogobius brunneus; **Location:** country: Japan; stateProvince: Iwate; locality: irrigation pond of the Kubo River Basin; verbatimLatitude: 38°55′30″N; verbatimLongitude: 141°03′24″E; **Identification:** identifiedBy: Yusuke Miyazaki; dateIdentified: 2009; **Event:** year: 2009; month: 5; day: 17; **Record Level:** basisOfRecord: PreservedSpecimen
- **Type status:** Other material. **Occurrence:** catalogNumber: KPM-NI 23948; recordedBy: Yusuke Miyazaki; individualCount: 1; **Taxon:** scientificName: Rhinogobius brunneus; **Location:** country: Japan; stateProvince: Iwate; locality: irrigation pond of the Kubo River Basin; verbatimLatitude: 38°55′33″N; verbatimLongitude: 141°01′53″E; **Identification:** identifiedBy: Yusuke Miyazaki; dateIdentified: 2009; **Event:** year: 2009; month: 8; day: 2; **Record Level:** basisOfRecord: PreservedSpecimen
- **Type status:** Other material. **Occurrence:** catalogNumber: KPM-NI 23950; recordedBy: Yusuke Miyazaki; individualCount: 1; **Taxon:** scientificName: Rhinogobius brunneus; **Location:** country: Japan; stateProvince: Iwate; locality: irrigation pond of the Kubo River Basin; verbatimLatitude: 38°55′37″N; verbatimLongitude: 141°01′49″E; **Identification:** identifiedBy: Yusuke Miyazaki; dateIdentified: 2009; **Event:** year: 2009; month: 8; day: 2; **Record Level:** basisOfRecord: PreservedSpecimen
- **Type status:** Other material. **Occurrence:** catalogNumber: KPM-NI 23949; recordedBy: Yusuke Miyazaki; individualCount: 1; **Taxon:** scientificName: Rhinogobius brunneus; **Location:** country: Japan; stateProvince: Iwate; locality: irrigation pond of the Tochikura River Basin; verbatimLatitude: 38°54′37″N; verbatimLongitude: 141°00′34″E; **Identification:** identifiedBy: Yusuke Miyazaki; dateIdentified: 2009; **Event:** year: 2009; month: 5; day: 20; **Record Level:** basisOfRecord: PreservedSpecimen
- **Type status:** Other material. **Occurrence:** catalogNumber: KPM-NI 23951; recordedBy: Yusuke Miyazaki; individualCount: 1; **Taxon:** scientificName: Rhinogobius brunneus; **Location:** country: Japan; stateProvince: Iwate; locality: irrigation pond of the Kubo River Basin; verbatimLatitude: 38°56′45″N; verbatimLongitude: 141°00′08″E; **Identification:** identifiedBy: Yusuke Miyazaki; dateIdentified: 2009; **Event:** year: 2009; month: 5; day: 2; **Record Level:** basisOfRecord: PreservedSpecimen
- **Type status:** Other material. **Occurrence:** catalogNumber: KPM-NI 23987; recordedBy: Yusuke Miyazaki; individualCount: 1; **Taxon:** scientificName: Rhinogobius brunneus; **Location:** country: Japan; stateProvince: Iwate; locality: irrigation pond of the Kubo River Basin; verbatimLatitude: 38°55′14″N; verbatimLongitude: 141°01′13″E; **Identification:** identifiedBy: Yusuke Miyazaki; dateIdentified: 2009; **Event:** year: 2009; month: 5; day: 3; **Record Level:** basisOfRecord: PreservedSpecimen
- **Type status:** Other material. **Occurrence:** catalogNumber: KPM-NI 23988; recordedBy: Yusuke Miyazaki; individualCount: 1; **Taxon:** scientificName: Rhinogobius brunneus; **Location:** country: Japan; stateProvince: Iwate; locality: irrigation pond of the Kubo River Basin; verbatimLatitude: 38°55′14″N; verbatimLongitude: 141°01′13″E; **Identification:** identifiedBy: Yusuke Miyazaki; dateIdentified: 2009; **Event:** year: 2009; month: 5; day: 15; **Record Level:** basisOfRecord: PreservedSpecimen
- **Type status:** Other material. **Occurrence:** catalogNumber: KPM-NI 24405; recordedBy: Yusuke Miyazaki; individualCount: 1; **Taxon:** scientificName: Rhinogobius brunneus; **Location:** country: Japan; stateProvince: Iwate; municipality: Dougasawa, Hagishou; verbatimLatitude: 38°56′32″N; verbatimLongitude: 141°01′57″E; **Identification:** identifiedBy: Yusuke Miyazaki; dateIdentified: 2009; **Event:** year: 2009; month: 9; day: 20; **Record Level:** basisOfRecord: PreservedSpecimen
- **Type status:** Other material. **Occurrence:** catalogNumber: KPM-NI 24407; recordedBy: Shogo Nishihara; individualCount: 1; **Taxon:** scientificName: Rhinogobius brunneus; **Location:** country: Japan; stateProvince: Iwate; locality: irrigation pond of the Tochikura River Basin; verbatimLatitude: 38°54′15″N; verbatimLongitude: 141°00′28″E; **Identification:** identifiedBy: Yusuke Miyazaki; dateIdentified: 2009; **Event:** year: 2009; month: 9; day: 21; **Record Level:** basisOfRecord: PreservedSpecimen
- **Type status:** Other material. **Occurrence:** catalogNumber: KPM-NI 24418; recordedBy: Yusuke Miyazaki; individualCount: 1; **Taxon:** scientificName: Rhinogobius brunneus; **Location:** country: Japan; stateProvince: Iwate; locality: irrigation pond of the Tochikura River Basin; verbatimLatitude: 38°54′32″N; verbatimLongitude: 141°00′50″E; **Identification:** identifiedBy: Yusuke Miyazaki; dateIdentified: 2009; **Event:** year: 2009; month: 9; day: 21; **Record Level:** basisOfRecord: PreservedSpecimen
- **Type status:** Other material. **Occurrence:** catalogNumber: KPM-NI 24463; recordedBy: Yusuke Miyazaki; individualCount: 1; **Taxon:** scientificName: Rhinogobius brunneus; **Location:** country: Japan; stateProvince: Iwate; municipality: Dougasawa, Hagishou; verbatimLatitude: 38°56′12″N; verbatimLongitude: 141°02′03″E; **Identification:** identifiedBy: Yusuke Miyazaki; dateIdentified: 2009; **Event:** year: 2009; month: 9; day: 21; **Record Level:** basisOfRecord: PreservedSpecimen
- **Type status:** Other material. **Occurrence:** catalogNumber: KPM-NI 24467; recordedBy: Yusuke Miyazaki; individualCount: 1; **Taxon:** scientificName: Rhinogobius brunneus; **Location:** country: Japan; stateProvince: Iwate; locality: irrigation pond of the Kubo River Basin; verbatimLatitude: 38°56′32″N; verbatimLongitude: 140°59′49″E; **Identification:** identifiedBy: Yusuke Miyazaki; dateIdentified: 2009; **Event:** year: 2009; month: 9; day: 22; **Record Level:** basisOfRecord: PreservedSpecimen
- **Type status:** Other material. **Occurrence:** catalogNumber: KPM-NI 24994; recordedBy: Yusuke Miyazaki; individualCount: 4; **Taxon:** scientificName: Rhinogobius brunneus; **Location:** country: Japan; stateProvince: Iwate; locality: irrigation pond of the Kubo River Basin; verbatimLatitude: 38°56′16″N; verbatimLongitude: 141°01′00″E; **Identification:** identifiedBy: Yusuke Miyazaki; dateIdentified: 2009; **Event:** year: 2008; month: 10; day: 8; **Record Level:** basisOfRecord: PreservedSpecimen
- **Type status:** Other material. **Occurrence:** catalogNumber: NSMT-P 90703; recordedBy: Yusuke Miyazaki; individualCount: 3; **Taxon:** scientificName: Rhinogobius brunneus; **Location:** country: Japan; stateProvince: Iwate; municipality: Hagishou; verbatimLatitude: 38°55′27″N; verbatimLongitude: 141°02′54″E; **Identification:** identifiedBy: Yusuke Miyazaki; dateIdentified: 2008; **Event:** year: 2008; month: 4; day: 30; **Record Level:** basisOfRecord: PreservedSpecimen
- **Type status:** Other material. **Occurrence:** catalogNumber: NSMT-P 90704; recordedBy: Yusuke Miyazaki; individualCount: 1; **Taxon:** scientificName: Rhinogobius brunneus; **Location:** country: Japan; stateProvince: Iwate; municipality: Hagishou; verbatimLatitude: 38°55′23″N; verbatimLongitude: 141°03′13″E; **Identification:** identifiedBy: Yusuke Miyazaki; dateIdentified: 2008; **Event:** year: 2008; month: 4; day: 30; **Record Level:** basisOfRecord: PreservedSpecimen
- **Type status:** Other material. **Occurrence:** catalogNumber: NSMT-P 90705; recordedBy: Yusuke Miyazaki; individualCount: 1; **Taxon:** scientificName: Rhinogobius brunneus; **Location:** country: Japan; stateProvince: Iwate; municipality: Hagishou; verbatimLatitude: 38°55′28″N; verbatimLongitude: 141°03′01″E; **Identification:** identifiedBy: Yusuke Miyazaki; dateIdentified: 2008; **Event:** year: 2008; month: 4; day: 30; **Record Level:** basisOfRecord: PreservedSpecimen
- **Type status:** Other material. **Occurrence:** catalogNumber: NSMT-P 90706; recordedBy: Yusuke Miyazaki; individualCount: 1; **Taxon:** scientificName: Rhinogobius brunneus; **Location:** country: Japan; stateProvince: Iwate; municipality: Hagishou; verbatimLatitude: 38°55′28″N; verbatimLongitude: 141°03′03″E; **Identification:** identifiedBy: Yusuke Miyazaki; dateIdentified: 2008; **Event:** year: 2008; month: 4; day: 30; **Record Level:** basisOfRecord: PreservedSpecimen
- **Type status:** Other material. **Occurrence:** catalogNumber: NSMT-P 90707; recordedBy: Yusuke Miyazaki; individualCount: 1; **Taxon:** scientificName: Rhinogobius brunneus; **Location:** country: Japan; stateProvince: Iwate; municipality: Hagishou; verbatimLatitude: 38°55′37″N; verbatimLongitude: 141°02′32″E; **Identification:** identifiedBy: Yusuke Miyazaki; dateIdentified: 2008; **Event:** year: 2008; month: 4; day: 30; **Record Level:** basisOfRecord: PreservedSpecimen
- **Type status:** Other material. **Occurrence:** catalogNumber: NSMT-P 90709; recordedBy: Yusuke Miyazaki; individualCount: 2; **Taxon:** scientificName: Rhinogobius brunneus; **Location:** country: Japan; stateProvince: Iwate; municipality: Hagishou; verbatimLatitude: 38°55′48″N; verbatimLongitude: 141°02′19″E; **Identification:** identifiedBy: Yusuke Miyazaki; dateIdentified: 2008; **Event:** year: 2008; month: 5; day: 1; **Record Level:** basisOfRecord: PreservedSpecimen
- **Type status:** Other material. **Occurrence:** catalogNumber: NSMT-P 90712; recordedBy: Yusuke Miyazaki; individualCount: 1; **Taxon:** scientificName: Rhinogobius brunneus; **Location:** country: Japan; stateProvince: Iwate; municipality: Hagishou; verbatimLatitude: 38°55′49″N; verbatimLongitude: 141°02′14″E; **Identification:** identifiedBy: Yusuke Miyazaki; dateIdentified: 2008; **Event:** year: 2008; month: 5; day: 1; **Record Level:** basisOfRecord: PreservedSpecimen
- **Type status:** Other material. **Occurrence:** catalogNumber: NSMT-P 90713; recordedBy: Yusuke Miyazaki; individualCount: 1; **Taxon:** scientificName: Rhinogobius brunneus; **Location:** country: Japan; stateProvince: Iwate; municipality: Hagishou; verbatimLatitude: 38°56′49″N; verbatimLongitude: 141°00′11″E; **Identification:** identifiedBy: Yusuke Miyazaki; dateIdentified: 2008; **Event:** year: 2008; month: 5; day: 1; **Record Level:** basisOfRecord: PreservedSpecimen
- **Type status:** Other material. **Occurrence:** catalogNumber: NSMT-P 90714; recordedBy: Yusuke Miyazaki; individualCount: 1; **Taxon:** scientificName: Rhinogobius brunneus; **Location:** country: Japan; stateProvince: Iwate; municipality: Hagishou; verbatimLatitude: 38°56′49″N; verbatimLongitude: 141°00′12″E; **Identification:** identifiedBy: Yusuke Miyazaki; dateIdentified: 2008; **Event:** year: 2008; month: 5; day: 1; **Record Level:** basisOfRecord: PreservedSpecimen
- **Type status:** Other material. **Occurrence:** catalogNumber: NSMT-P 90718; recordedBy: Yusuke Miyazaki; individualCount: 2; **Taxon:** scientificName: Rhinogobius brunneus; **Location:** country: Japan; stateProvince: Iwate; municipality: Hagishou; verbatimLatitude: 38°55′47″N; verbatimLongitude: 140°57′13″E; **Identification:** identifiedBy: Yusuke Miyazaki; dateIdentified: 2008; **Event:** year: 2008; month: 5; day: 1; **Record Level:** basisOfRecord: PreservedSpecimen
- **Type status:** Other material. **Occurrence:** catalogNumber: NSMT-P 90723; recordedBy: Yusuke Miyazaki; individualCount: 2; **Taxon:** scientificName: Rhinogobius brunneus; **Location:** country: Japan; stateProvince: Iwate; municipality: Hagishou; verbatimLatitude: 38°55′29″N; verbatimLongitude: 141°00′19″E; **Identification:** identifiedBy: Yusuke Miyazaki; dateIdentified: 2008; **Event:** year: 2008; month: 5; day: 1; **Record Level:** basisOfRecord: PreservedSpecimen
- **Type status:** Other material. **Occurrence:** catalogNumber: NSMT-P 91208; recordedBy: Yusuke Miyazaki; individualCount: 1; **Taxon:** scientificName: Rhinogobius brunneus; **Location:** country: Japan; stateProvince: Iwate; locality: Ichinono River; **Identification:** identifiedBy: Yusuke Miyazaki; dateIdentified: 2008; **Event:** year: 2008; month: 7; day: 10; **Record Level:** basisOfRecord: PreservedSpecimen
- **Type status:** Other material. **Occurrence:** catalogNumber: NSMT-P 91648; recordedBy: Yusuke Miyazaki; individualCount: 1; **Taxon:** scientificName: Rhinogobius brunneus; **Location:** country: Japan; stateProvince: Iwate; locality: irrigation pond of the Kubo River Basin; **Identification:** identifiedBy: Yusuke Miyazaki; dateIdentified: 2008; **Event:** year: 2008; month: 7; day: 12; **Record Level:** basisOfRecord: PreservedSpecimen
- **Type status:** Other material. **Occurrence:** catalogNumber: NSMT-P 96059; recordedBy: Yusuke Miyazaki; individualCount: 1; **Taxon:** scientificName: Rhinogobius brunneus; **Location:** country: Japan; stateProvince: Iwate; locality: irrigation pond of the Tochikura River Basin; verbatimLatitude: 38°54′36″N; verbatimLongitude: 141°01′07″E; **Identification:** identifiedBy: Yusuke Miyazaki; dateIdentified: 2008; **Event:** year: 2008; month: 8; day: 24; **Record Level:** basisOfRecord: PreservedSpecimen
- **Type status:** Other material. **Occurrence:** catalogNumber: NSMT-P 96061; recordedBy: Yusuke Miyazaki; individualCount: 1; **Taxon:** scientificName: Rhinogobius brunneus; **Location:** country: Japan; stateProvince: Iwate; locality: irrigation pond of the Kubo River Basin; verbatimLatitude: 38°55′03″N; verbatimLongitude: 141°00′25″E; **Identification:** identifiedBy: Yusuke Miyazaki; dateIdentified: 2008; **Event:** year: 2009; month: 4; day: 30; **Record Level:** basisOfRecord: PreservedSpecimen
- **Type status:** Other material. **Occurrence:** catalogNumber: NSMT-P 96853; recordedBy: Yusuke Miyazaki and Yoko Takata; individualCount: 1; **Taxon:** scientificName: Rhinogobius brunneus; **Location:** country: Japan; stateProvince: Iwate; locality: irrigation pond of the Kubo River Basin; **Identification:** identifiedBy: Yoko Takata; dateIdentified: 2010; **Event:** year: 2008; month: 10; day: 5; **Record Level:** basisOfRecord: PreservedSpecimen
- **Type status:** Other material. **Occurrence:** catalogNumber: NSMT-P 96854; recordedBy: Yusuke Miyazaki and Yoko Takata; individualCount: 1; **Taxon:** scientificName: Rhinogobius brunneus; **Location:** country: Japan; stateProvince: Iwate; locality: irrigation pond of the Kubo River Basin; **Identification:** identifiedBy: Yoko Takata; dateIdentified: 2010; **Event:** year: 2008; month: 10; day: 5; **Record Level:** basisOfRecord: PreservedSpecimen
- **Type status:** Other material. **Occurrence:** catalogNumber: NSMT-P 96858; recordedBy: Yusuke Miyazaki and Yoko Takata; individualCount: 1; **Taxon:** scientificName: Rhinogobius brunneus; **Location:** country: Japan; stateProvince: Iwate; municipality: Hagishou; **Identification:** identifiedBy: Yusuke Miyazaki; dateIdentified: 2008; **Event:** year: 2008; month: 10; day: 8; **Record Level:** basisOfRecord: PreservedSpecimen
- **Type status:** Other material. **Occurrence:** catalogNumber: NSMT-P 96859; recordedBy: Yusuke Miyazaki and Yoko Takata; individualCount: 2; **Taxon:** scientificName: Rhinogobius brunneus; **Location:** country: Japan; stateProvince: Iwate; municipality: Hagishou; **Identification:** identifiedBy: Yusuke Miyazaki; dateIdentified: 2008; **Event:** year: 2008; month: 10; day: 8; **Record Level:** basisOfRecord: PreservedSpecimen
- **Type status:** Other material. **Occurrence:** catalogNumber: NSMT-P 97111; recordedBy: Yusuke Miyazaki and Yoko Takata; individualCount: 4; **Taxon:** scientificName: Rhinogobius brunneus; **Location:** country: Japan; stateProvince: Iwate; locality: irrigation pond of the Kubo River Basin; **Identification:** identifiedBy: Masanori Nakae; dateIdentified: 2010; **Event:** year: 2008; month: 10; day: 5; **Record Level:** basisOfRecord: PreservedSpecimen

##### Distribution

Japan, Taiwan, Korea, China, Philippines, and Vietnam.

##### Notes

Japanese researchers have considered that the Rhinogobius brunneus complex currently includes many valid, synonymous species,and potentially several undescribed species (Akihito et al. 2002, Akihito et al. 2013). This taxon of this region matches the "*Rhinogobius* sp. OR of Akihito et al. (2002)" species complex (see Akihito et al. 2002, Akihito et al. 2013). It was recorded only from lentic waters including irrigation ponds, channels, and slow moving parts of rivers in the present study.

## Analysis

Following the literature review, 35 freshwater fish taxa, including subspecies, belonging to 12 families in eight orders were considered to occur naturally in Iwate Prefecture (Table 1). Of these, 24 species/subspecies were determined to constitute the potential species pool of the survey area following our literature review (Table 1).

Twenty freshwater fish species/subspecies belonging to 9 families in 6 orders were recorded during field surveys (Figs 3, 4, 5, 6, 7; Table 1); 12 species/subspecies belong to Cypriniformes (62% of the total), 3 to Perciformes (14%), 2 to Salmoniformes (10%), and 1 each to Petromyzontiformes, Siluriformes, and Beloniformes (5% each). At the family level, Cyprinidae had the highest number of representatives (11, 52% of the total), followed by Cobitidae and Salmonidae (2, 10% each). Petromyzontidae, Bagridae, Adrianichthyidae, Cottidae, and Gobiidae were represented by one species each (5%). Three species/subspecies, *Zacco platypus*, *Pseudogobio esocinus esocinus*, and *Oncorhynchus keta*, were not recorded upstream of the dams constructed in the lower reaches of the Kubo and Tochikura Rivers.

Among the 20 species collected in the field were nine national/prefectural red list species (*Lethenteron reissneri*, *Carassius auratus buergeri*, *Pseudorasbora pumila*, *Pseudogobio esocinus esocinus*, *Misgurnus anguillicaudatus*, *Tachysurus tokiensis*, *Oncorhynchus masou masou*, *Oryzias latipes*, and *Cottus nozawae*: Iwate Prefecture 2014; Ministry of the Environment, Japan 2013), three naturalized non-native species (*Carassius cuvieri*, *Zacco platypus*, and *Pseudorasbora parva*: Matsuzawa and Senou 2008], and three non-native invasive species (*Cyprinus rubrofuscus*, *Rhodeus ocellatus ocellatus*, and *Micropterus salmoides*: Lowe et al. 2000, Murakami and Washitani 2002) (Table 1).

The rarefaction curves of survey data from irrigation ponds and rivers are shown in Fig. 8; the expected saturation point of species richness was reached.

## Discussion

Only 13 of the 24 species/subspecies from our estimated potential species pool were collected during field surveys, including nine national/prefectural red list species (Table 1). In addition, seven non-native species, including one national/prefectural red list species and three non-native invasive species, were recorded (Table 1).

Ten species/subspecies of the potential species pool for the survey area were not recorded during field surveys (Table 1). Of these, *Lethenteron camtschaticum* (Tilesius, 1811), *Anguilla japonica* Temminck & Schlegel, 1847, *Tribolodon brandtii* (Dybowski, 1872), *Plecoglossus altivelis altivelis* (Temminck & Schlegel, 1846), *Salvelinus leucomaenis* subspp., and *Rhinogobius nagoyae* Jordan & Seale, 1906 were most likely missed due to the limited number of sampling points in the rivers. The remaining uncaptured species/subspecies in the potential species pool (i.e., *Tribolodon ezoe* Okada & Ikeda, 1937, *Lefua echigonia* Jordan & Richardson, 1907, and *Cottus pollux* Günter, 1873) were considered to have a low probability of occurrence in the study region due to a limited distribution in Tohoku region (they depend on sea currents for their migration and/or were near the boundaries of their distributional ranges) (Kawanabe et al. 2001, Ozawa et al. 2003, Nakabo 2013). It is also possiblethat our predicted species pool for the survey region was inaccurately estimateddue to a lack of data regarding biological interactions, heterogeneity of environments, and quality/quantity of the various habitats. In other words, some species included in the potential species pool might well have been excluded if we had more data on their ecology, particularly with respect to their biological interactions with other fishes. There is also the chance of local extirpation.

The possibility that insufficient sampling efforts influenced the results of the present study is supported by the rarefaction curve as described below. The curve analyzed by using the presence/absence data of the irrigation ponds suggests that no more native species will be recorded from the ponds of the region due to the curve reaching saturation (Fig. 8a). However, the rarefaction curve of the rivers suggests that 1–3 additional nativepecies could be recorded based on the curve's saturation point and its 95% CI upper bound (Fig. 8b). It is also important to state that the species richness estimates assume that our survey techniques have an equal probability of capturing every species, but this assumption is likely to be violated in practice due to the different characteristics of each species (i.e. small benthic species or large active species maybe harder to catch).

The Kubo and Tochikura Rivers are both dammed, and *Zacco platypus*, *Pseudogobio esocinus esocinus*, and *Oncorhynchus keta* were not recorded upstream of the dams. This suggests that the dams have negatively affected migratory behavior in these species (although *Z. platypus* is a naturalized non-native species).

The meta-population of *Oryzias latipes* in the study region was possibly introduced by local residents. The altitude at which it was recorded (105–160 m) is perhaps too high to consider this a natural occurrence. Populations of *O. latipes* in Kanagawa Prefecture naturally occur at a height of ~30 m above sea level (Senou unpublished), for example. Additionally, the specimens collected did not exhibit breeding condition, except at one sampling site (Miyazaki 2010). According to the local residents, these non-breeding populations probably all originated from the same source near the river system. This species is highly threatened and locally extirpated in many parts of Iwate Prefecture, including elsewhere in the river system studied here, and more generally in Japan (Ministry of the Environment, Japan 2013, Iwate Prefecture 2014). Therefore, we regard these populations of *O. latipes* as a target for ex-situ conservation because of the high vulnerability of the original populations and the less damaging ecological effects in the study region.

Although we tentatively identified all specimens of the genus *Carassius*, except for *C. cuvieri*, as *C. auratus buergeri*, the specimens display some morphological variability. One specimen (KPM-NI 23745: Fig. 3e) has 16 soft, branched dorsal-fin rays (vs. 12–14 in *C. a. b.*), and the other specimens have an angular jugular (lower jaw). Within Japanese crucian carps, the angular jugular is one of the specific characters of *Carassius auratus grandoculis* Temminck & Schlegel, 1846 (Hosoya 2013), whereas Takahashi (2003) reported that *C. a. b.* populations from irrigation ponds of Ehime Prefecture tend to have an angular jugular. Further information on the morphology, molecular biology, and biogeography of Japanese crucian carps is urgently needed, because they include national and regional red list species and/or populations (Ministry of the Environment, Japan 2013; Iwate Prefecture 2014). In fact, Dr. Nakamura, who published the current taxonomy of Japanese crucian carps (Nakamura 1969), indicated that morphological variations and distributional ranges of *C. a. b.* in northern Japan are in need of clarification (Nakamura 1969, Nakamura 1976).

Among the red list species recorded in the present study, *Lethenteron reissneri*, *Pseudorasbora pumila*, *Tachysurus tokiensis*, *Oryzias latipes*, and *Cottus nozawae* (threatened species) were rare and are considered vulnerable. Further research of these species is required, particularly with regard to their population dynamics and conservation in the Kubo-gawa Ihatov area. Similarly, the population dynamics and extended distributions of the non-native and invasive species should be studied in greater detail with a special focus on potential methods of removal. In particular: *Cyprinus rubrofuscus*, which has been reported as having highly negative effects on native macrophytes and odonates in the region (Miyazaki et al. 2010, Sekizaki et al. 2012, Yoshioka et al. 2014); *Pseudorasbora parva*, which has replaced *P. pumila* following hybridization in many localities (Konishi and Takata 2004, Konishi 2010); and *Micropterus salmoides*, which has apparently been illegally introduced by sport fishermen and/or residents (Miyazaki 2010, Tsunoda et al. 2011).

We refer the above suggestions to the focal nature restoration committee (see also Chisaka 2010). In other words, the localities of vulnerable species (i.e., *Lethenteron reissneri*, *Carassius auratus buergeri*, *Pseudorasbora pumila*, *Tachysurus tokiensis*, *Oryzias latipes*, and *Cottus nozawae*) will be preferentially conserved and that of non-native species that negatively affect sustainability of native species (i.e., *Cyprinus rubrofuscus*, *Pseudorasbora parva* and *Micropterus salmoides*) will be preferentially removed. In addition, we will further investigate suitable habitats in the target area to record the remaining uncaptured species/subspecies from the potential species pool. Therefore, the comparison of the potential species pool with the national and regional Red Lists provides useful suggestions for priority species that should be conserved or removed, and for determining further sampling sites with suitable habitat for the absent species (those listed in the potential species pool that were not yet collected in the field surveys).

The determination of the freshwater fish species pool of a given region is an essential first step in restoration ecology (Fattorini and Halle 2004, Hobbs and Norton 2004, Funk et al. 2008). When followed up with field surveys, desk surveys can lead to an improved understanding of regional biotas and their primary characteristics,permit prediction of potential extinction events caused by natural or anthropogenic factors, and provide insights regarding the geological history affecting freshwater fish distributions. Furthermore, many restoration projects are somewhat restricted by budgets, human resources, and/or methodology (e.g., Joseph et al. 2009, Carwardine et al. 2012), however, desk surveys are required as an important first step to determine the potential species pool. However, this information can be limited, as shown here, and precise information from field surveys can then feed back into improved estimates for future desk surveys and restoration ecology projects.

## Supplementary Material

XML Treatment for Lethenteron reissneri

XML Treatment for Cyprinus rubrofuscus

XML Treatment for Carassius cuvieri

XML Treatment for Carassius auratus buergeri

XML Treatment for Rhodeus ocellatus ocellatus

XML Treatment for Zacco platypus

XML Treatment for Rhynchocypris steindachneri

XML Treatment for Tribolodon hakonensis

XML Treatment for Pseudorasbora parva

XML Treatment for Pseudorasbora pumila pumila

XML Treatment for Pseudogobio esocinus esocinus

XML Treatment for Misgurnus anguillicaudatus

XML Treatment for Cobitis biwae

XML Treatment for Tachysurus tokiensis

XML Treatment for Oncorhynchus keta

XML Treatment for Oncorhynchus masou masou

XML Treatment for Oryzias latipes

XML Treatment for Micropterus salmoides

XML Treatment for Cottus nozawae

XML Treatment for Rhinogobius brunneus

## Figures and Tables

**Figure 2a.**
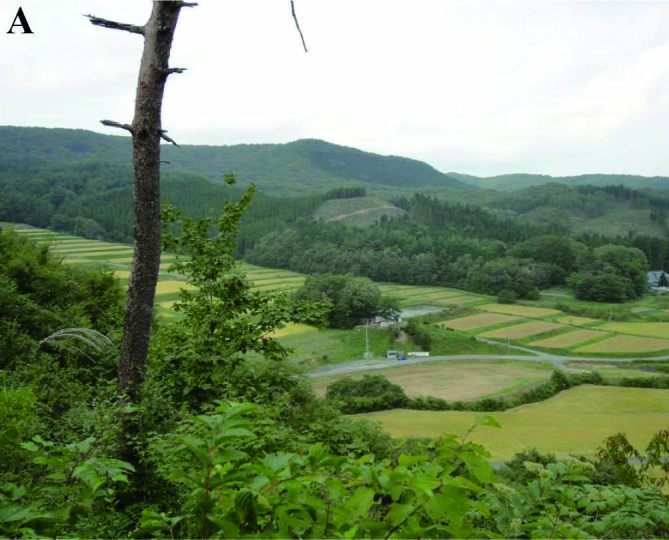
The typical landscape of the region.

**Figure 2b.**
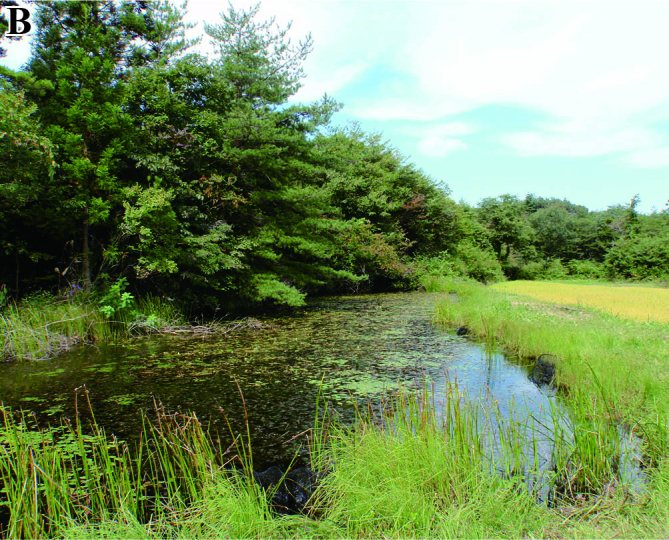
The irrigation pond where many aquatic plants, including several threatened species, occur.

**Figure 2c.**
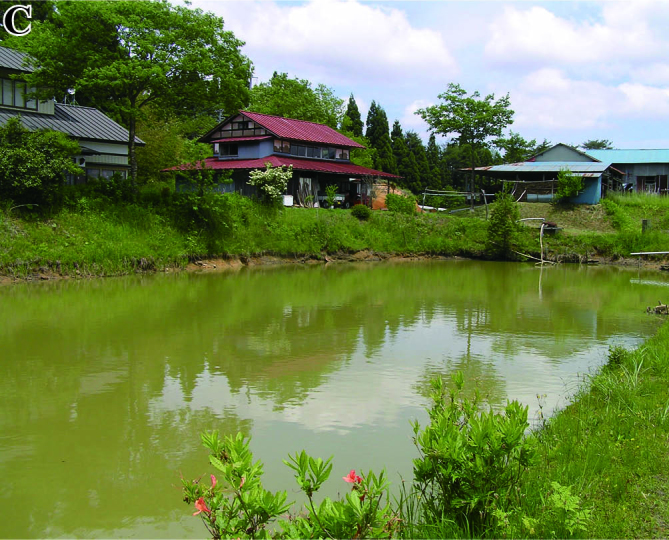
The irrigation pond that a carp population inhabits.

**Figure 2d.**
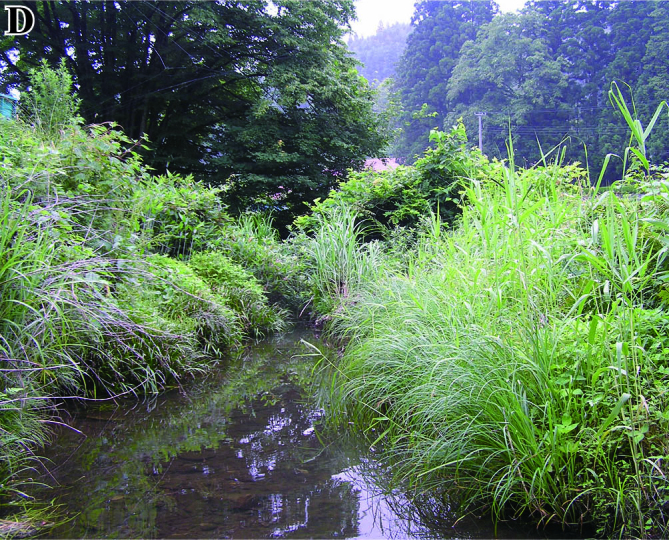
The upper reach of the Tochikura River.

**Figure 2e.**
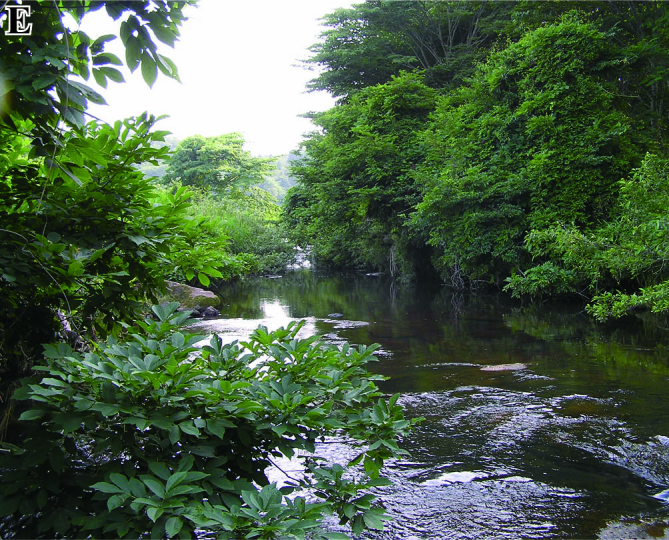
The middle reach of the Kubo River.

**Figure 2f.**
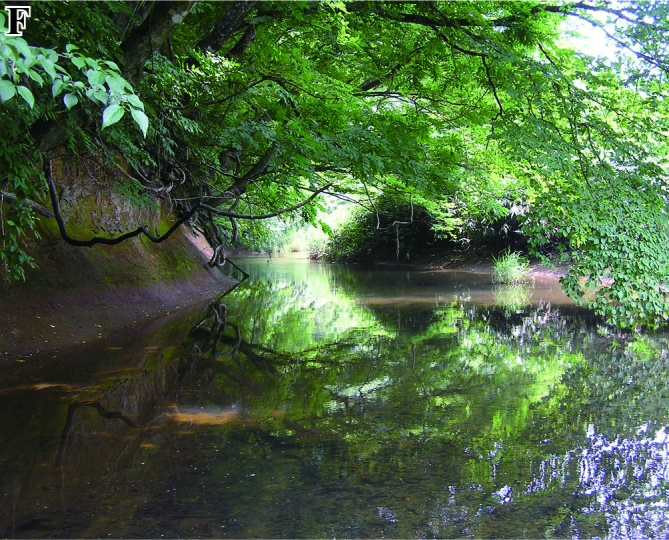
The lower reach of the Ichinono River.

**Figure 3a.**
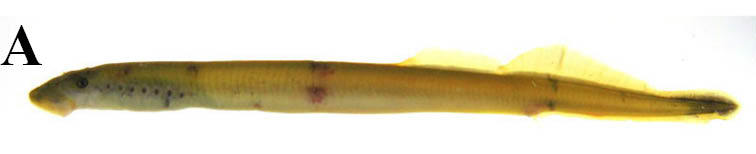
*Lethenteron reissneri*, KPM-NI 23996, 105.0 mm TL (adult).

**Figure 3b.**
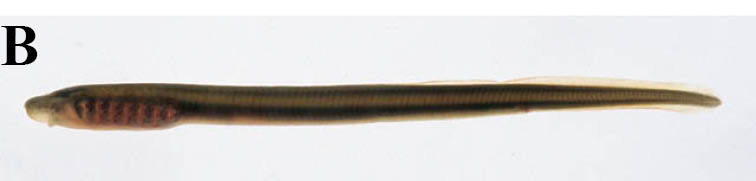
*Lethenteron reissneri*, KPM-NI 21186, 71.3 mm TL (ammocoete larva).

**Figure 3c.**
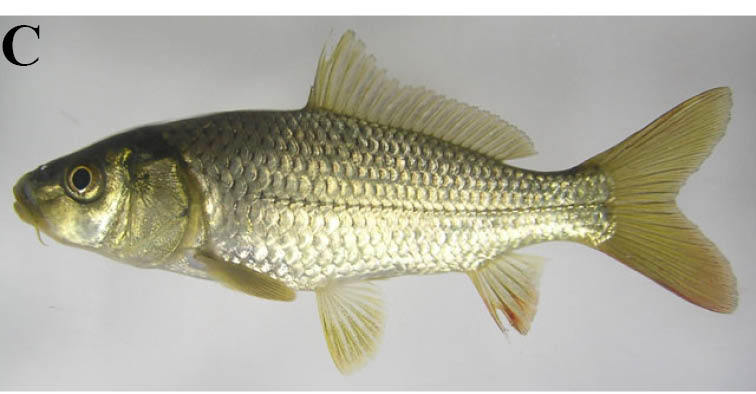
*Cyprinus rubrofuscus*, KPM-NI 22277, 108.9 mm SL.

**Figure 3d.**
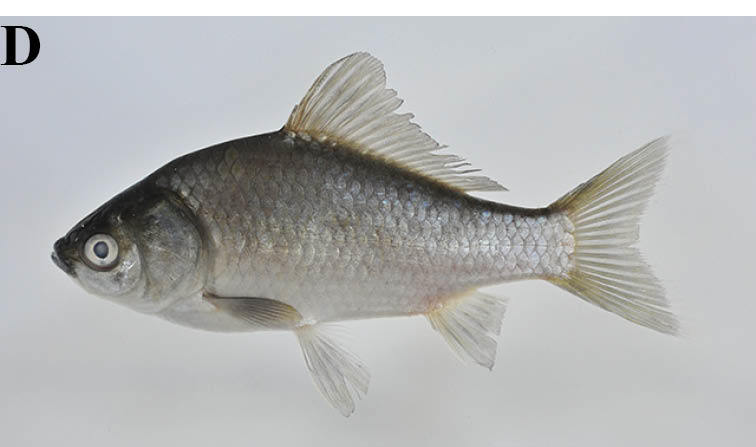
*Carassius cuvieri*, KPM-NI 30997, 127.0 mm SL.

**Figure 3e.**
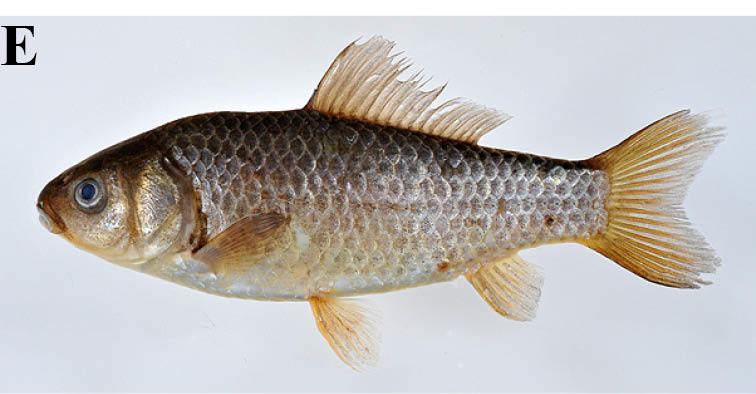
*Carassius auratus buergeri*, KPM-NI 23745, 132.7 mm SL.

**Figure 3f.**
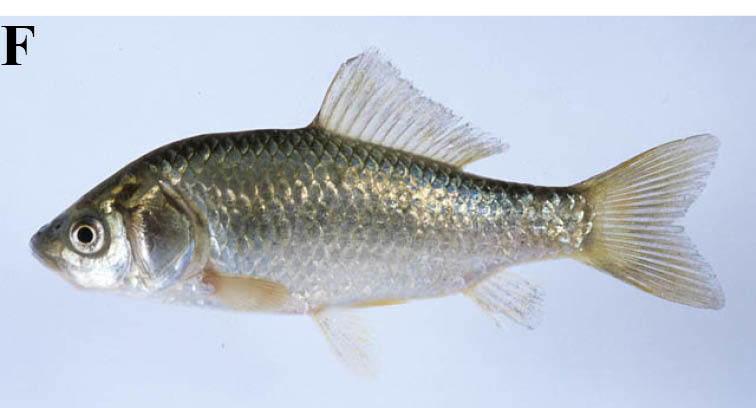
*Carassius auratus buergeri*, KPM-NI 21196, 83.6 mm SL.

**Figure 4a.**
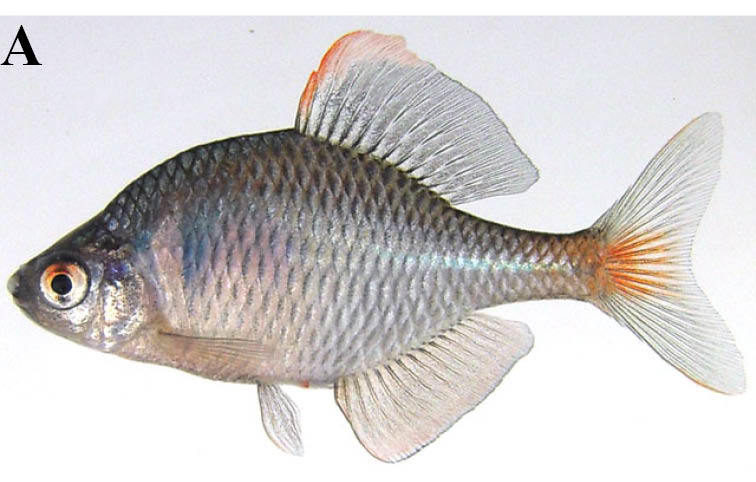
*Rhodeus ocellatus ocellatus*, KPM-NI 23983, 41.9 mm SL (male).

**Figure 4b.**
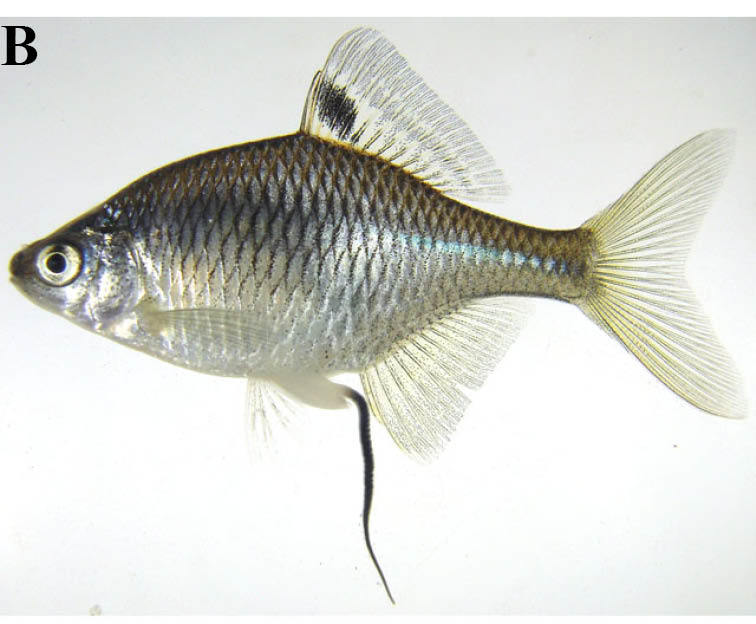
*Rhodeus ocellatus ocellatus*, KPM-NI 23982, 38.1 mm SL (female).

**Figure 4c.**
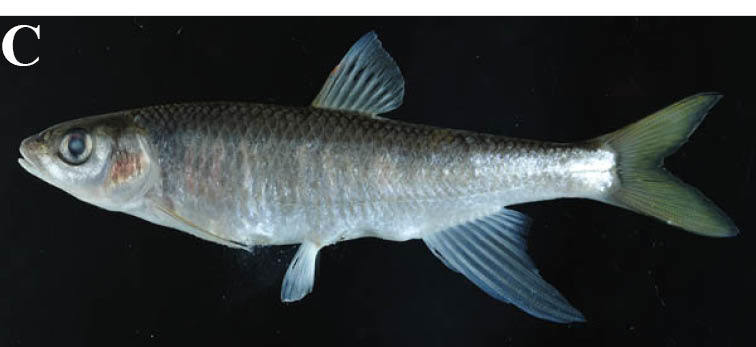
*Zacco platypus*, NSMT-P 96830, 94.1 mm SL.

**Figure 4d.**
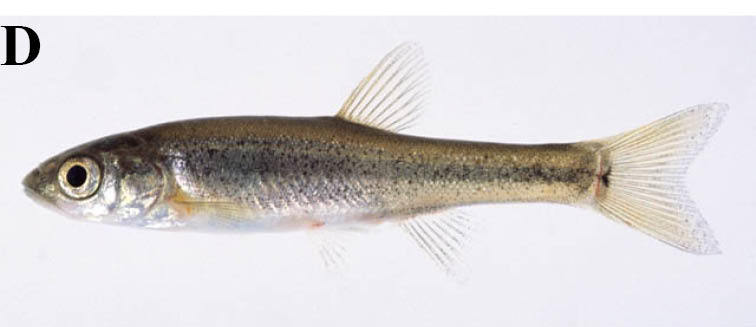
*Rhynchocypris steindachneri*, KPM-NI 21188, 35.9 mm SL.

**Figure 4e.**
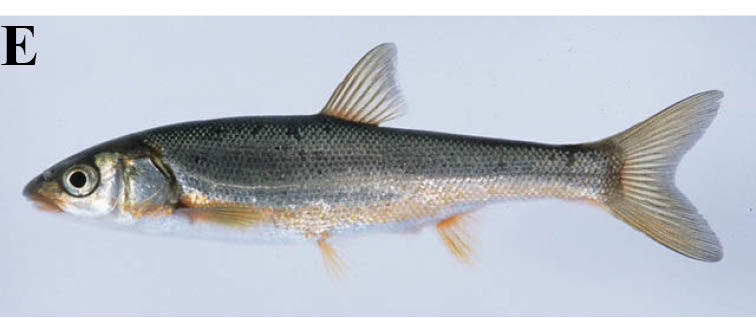
*Tribolodon hakonensis*, KPM-NI 21187, 85.3 mm SL.

**Figure 5a.**
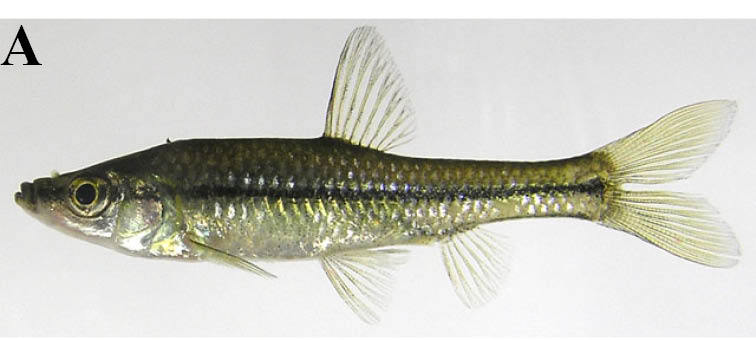
*Pseudorasbora parva*, KPM-NI22273, 46.3 mm SL.

**Figure 5b.**
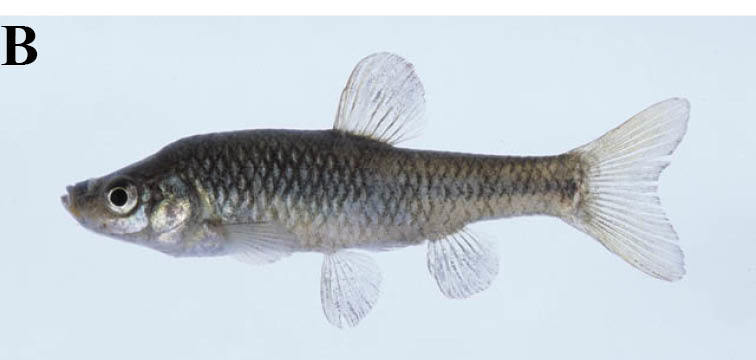
*Pseudorasbora pumila*, KPM-NI 21403, 51.5 mm SL.

**Figure 5c.**
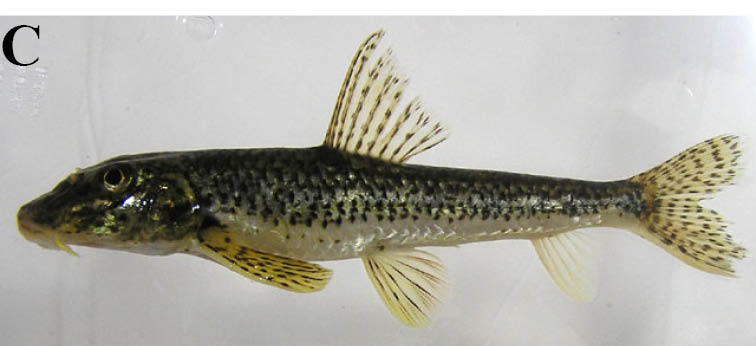
*Pseudogobio esocinus esocinus*, KPM-NI 22253, 118.6 mm SL.

**Figure 5d.**
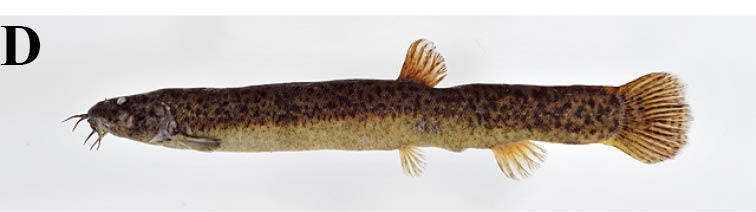
*Misgurnus anguillicaudatus*, KPM-NI 24399, 166.4 mm SL.

**Figure 5e.**
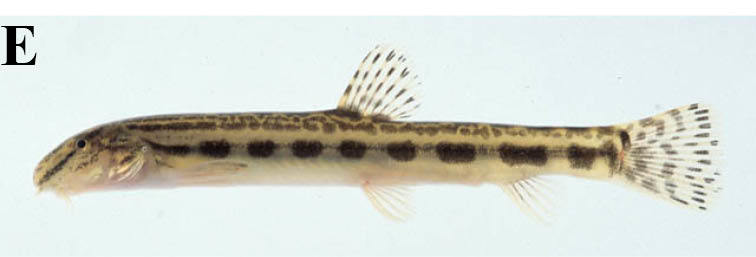
*Cobitis biwae*, KPM-NI 21190, 49.6 mm SL.

**Figure 5f.**
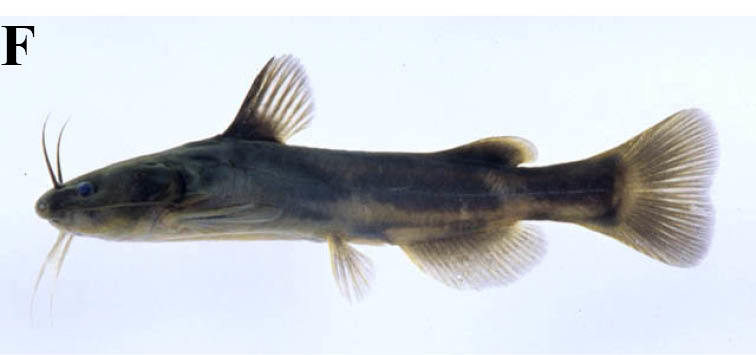
*Tachysurus tokiensis*, KPM-NI 19440, 74.4 mm SL.

**Figure 6a.**
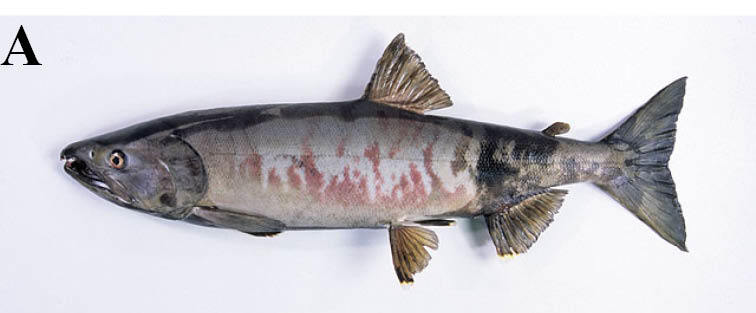
*Oncorhynchus keta*, KPM-NI 22429, 635.6 mm SL (male).

**Figure 6b.**
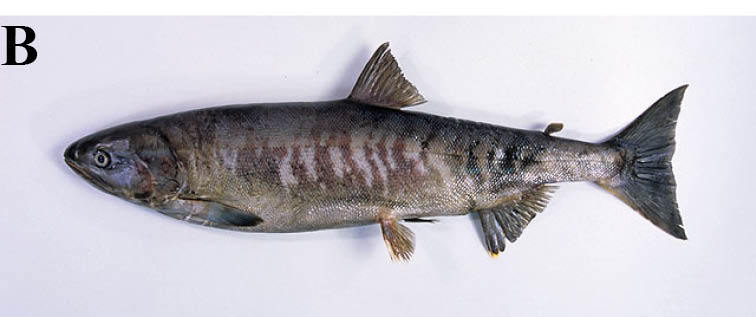
*Oncorhynchus keta*, KPM-NI 22430, 548.6 mm SL (female).

**Figure 6c.**
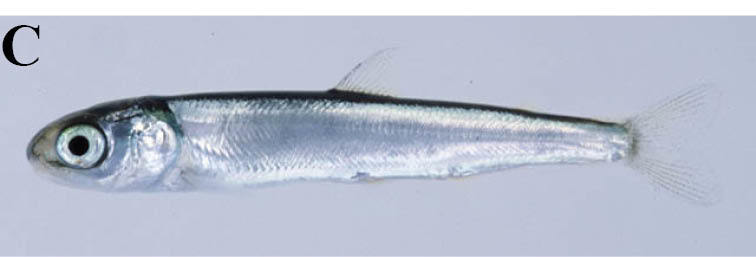
*Oncorhynchus keta*, KPM-NI 21183, 31.2 mm SL (juvenile).

**Figure 6d.**
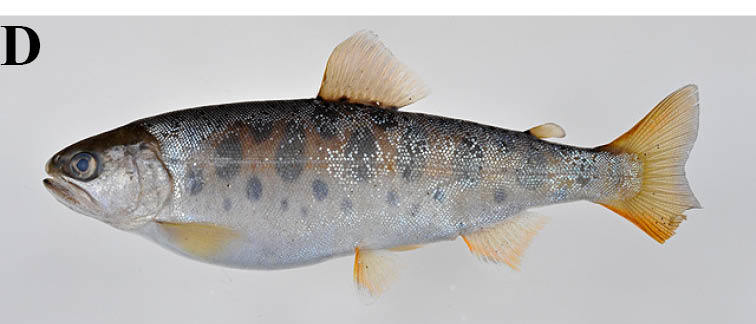
*Oncorhynchus masou masou*, KPM-NI 21181, 31.2 mm SL.

**Figure 6e.**
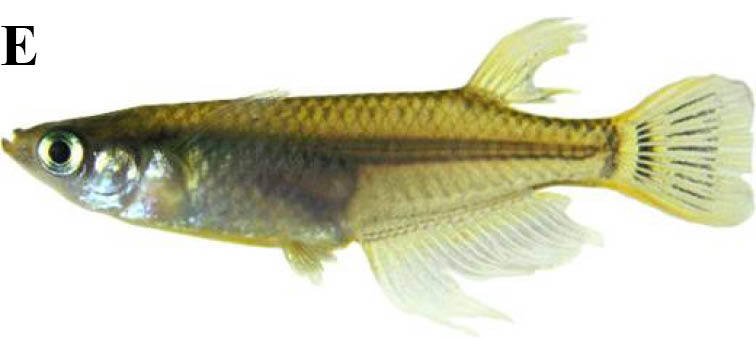
*Oryzias latipes*, KPM-NI 23979, 25.4 mm SL (male).

**Figure 6f.**
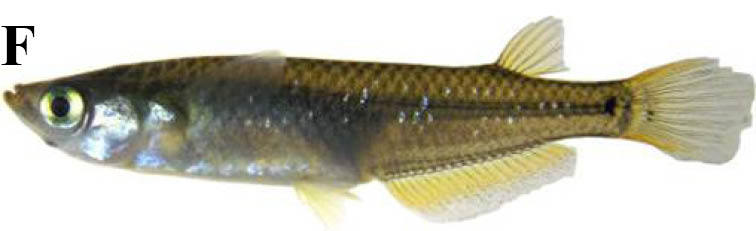
*Oryzias latipes*, KPM-NI 23981, 26.9 mm SL (female).

**Figure 7a.**
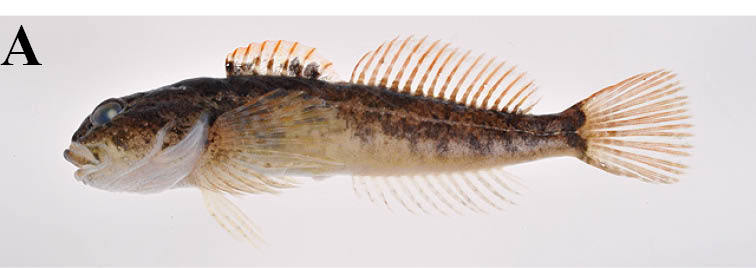
*Cottus nozawae*, KPM-NI 24408, 59.2 mm SL.

**Figure 7b.**
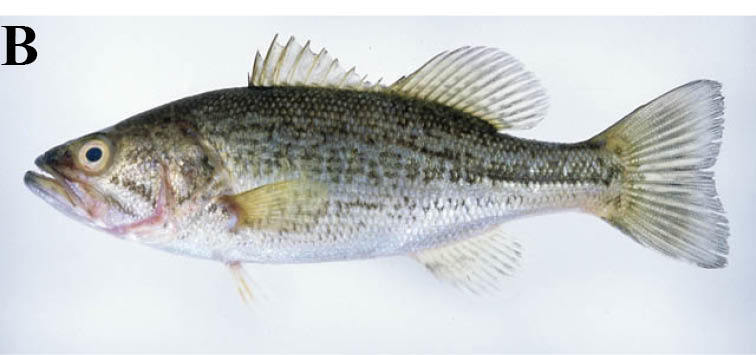
*Micropterus salmoides*, KPM-NI 21182, 122.9 mm SL.

**Figure 7c.**
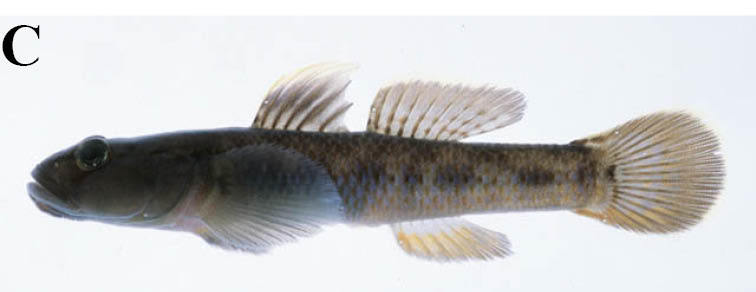
*Rhinogobius brunneus*, KPM-NI 21192, 39.0 mm SL (male).

**Figure 7d.**
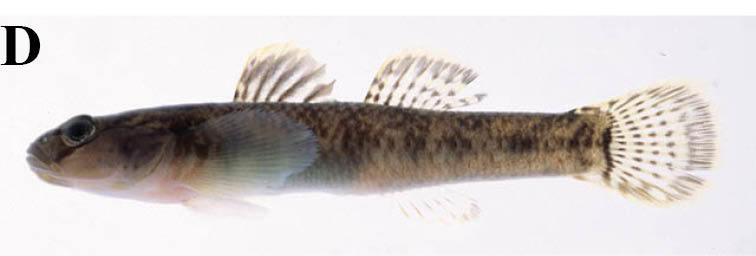
*Rhinogobius brunneus*, KPM-NI 21193, 37.9 mm SL (female).

**Figure 8a.**
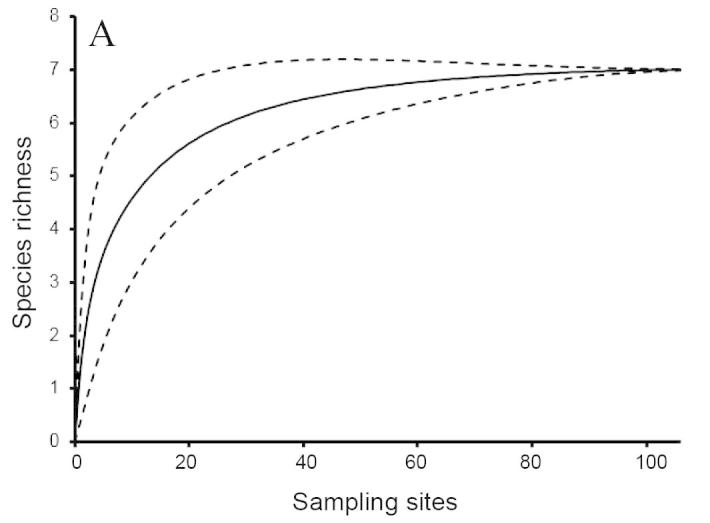
at the irrigation ponds

**Figure 8b.**
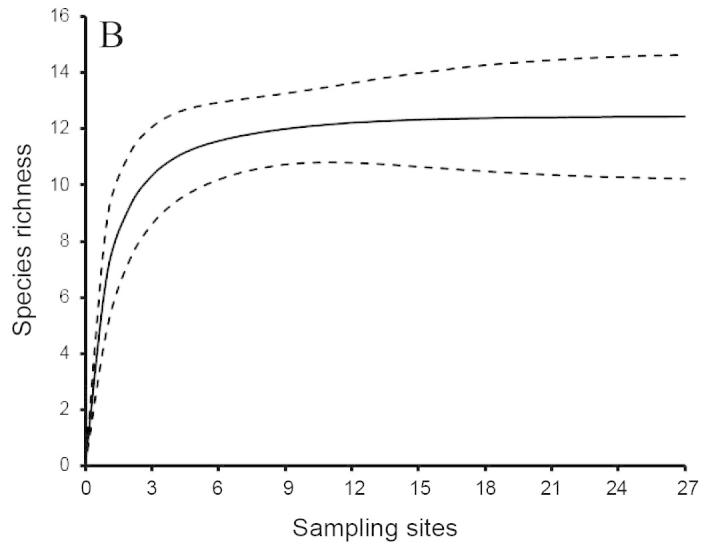
at the rivers

**Table 1.** List of fishes occurring naturally in Iwate Prefecture, northern Japan. From this list the potential species pool was determined by the literature review (ref. Kawanabe et al. 2001, Ozawa et al. 2003, Matsuzawa and Senou 2008, Kumagai and Katsushima 2012, Nakabo 2013​). CR, critically endangered; EN, endangered; VU, vulnerable; NT, near threatened; DD, data deficient; LC, least concern; LP, threatened local population; category of red lists (Ministry of the Environment, Japan 2013, Iwate Prefecture 2014). YES, NO; ^1^Listed as non-native species (Matsuzawa and Senou 2008, Nakabo 2013); ^2^Record from each study region based on the field surveys.

Taxon	Standard Japanese Name	National Red List	Prefectural Red List	Nonnative Species	Potential Species Pool	Kubo River	Tochikura River	Ichinono River	Ponds of Kubo Riv. Sys.	Ponds of Tochikura Riv. Sys.	Ponds of Ichinono Riv. Sys.	Literature
*Lethenteron camtschaticum*	Kawa-yastume	VU	CR+EN	NO	YES	NO	NO	NO	NO	NO	NO	Kawanabe et al. (2001), Nakabo and Kai (2013)
*Lethenteron reissneri*	Suna-yatsume	VU	NT	NO	YES	YES	YES	YES	NO	NO	NO	Kawanabe et al. (2001), Nakabo and Kai (2013)
*Anguilla japonica*	Nihon-unagi	EN		NO	YES	NO	NO	NO	NO	NO	NO	Kawanabe et al. (2001), Nakabo (2013)
*Cyprinus rubrofuscus*	Koi	-	-	YES	NO	YES	YES	NO	YES	YES	YES	Kawanabe et al. (2001), Ozawa et al. (2003), Matsuzawa and Senou (2008), Kumagai and Katsushima (2012), Hosoya (2013)
*Carassius cuvieri*	Gengorō-buna	-	-	YES	NO	NO	NO	NO	YES	NO	NO	Kawanabe et al. (2001), Ozawa et al. (2003), Matsuzawa and Senou (2008), Kumagai and Katsushima (2012), Hosoya (2013)
*C. auratus auratus*	Gin-buna			NO	YES	NO	NO	NO	NO	NO	NO	Kawanabe et al. (2001), Ozawa et al. (2003), Matsuzawa and Senou (2008), Kumagai and Katsushima (2012), Hosoya (2013)
*C. auratus buergeri*	Kin-buna	VU	NT	NO	YES	YES	NO	YES	YES	YES	YES	Kawanabe et al. (2001), Ozawa et al. (2003), Kumagai and Katsushima (2012), Hosoya (2013)
*Acheilognathus melanogaster*	Tanago	EN	LC	NO	NO	NO	NO	NO	NO	NO	NO	Kawanabe et al. (2001), Kumagai and Katsushima (2012), Hosoya (2013)
*A. typus*	Zeni-tanago	CR	CR+EN	NO	NO	NO	NO	NO	NO	NO	NO	Kawanabe et al. (2001), Ozawa et al. (2003), Matsuzawa and Senou (2008), Kumagai and Katsushima (2012), Hosoya (2013)
*Rhodeus ocellatus ocellatus*	Tairiku-bara-tanago	-	-	YES	NO	YES	NO	NO	YES	NO	NO	Kawanabe et al. (2001), Matsuzawa and Senou (2008), Kumagai and Katsushima (2012), Hosoya (2013)
*Zacco platypus*	Oikawa	-	-	YES	NO	YES	YES	YES	NO	NO	NO	Kawanabe et al. (2001), Ozawa et al. (2003), Matsuzawa and Senou (2008), Kumagai and Katsushima (2012), Hosoya (2013)
*Rhynchocypris steindachneri*	Abura-haya			NO	YES	YES	YES	YES	YES	YES	YES	Kawanabe et al. (2001), Kumagai and Katsushima (2012), Hosoya (2013)
*Tribolodon brandtii*	Maruta			NO	YES	NO	NO	NO	NO	NO	NO	Kawanabe et al. (2001), Hosoya (2013)
*T. ezoe*	Ezo-ugui	LP	NT	NO	YES	NO	NO	NO	NO	NO	NO	Kawanabe et al. (2001), Hosoya (2013)
*T. hakonensis*	Ugui			NO	YES	YES	YES	YES	NO	NO	NO	Kawanabe et al. (2001), Kumagai and Katsushima (2012), Hosoya (2013)
*Pseudorasbora parva*	Motsugo	-	-	YES	NO	YES	YES	NO	YES	YES	NO	Kawanabe et al. (2001), Ozawa et al. (2003), Matsuzawa and Senou (2008), Kumagai and Katsushima (2012), Hosoya (2013)
*P. pumila*	Shinai-motsugo	CR	CR+EN	NO	YES	NO	NO	NO	YES	NO	NO	Kawanabe et al. (2001), Ozawa et al. (2003), Matsuzawa and Senou (2008), Kumagai and Katsushima (2012), Hosoya (2013)
*Pseudogobio esocinus esocinus*	Kamatsuka		DD	NO	YES	NO	YES	YES	NO	NO	NO	Kawanabe et al. (2001), Ozawa et al. (2003), Matsuzawa and Senou (2008), Kumagai and Katsushima (2012), Hosoya (2013)
*Hemibarbus labeo*	Nigoi			NO	NO	NO	NO	NO	NO	NO	NO	Kawanabe et al. (2001), Matsuzawa and Senou (2008), Kumagai and Katsushima (2012), Hosoya (2013)
*Misgurnus anguillicaudatus*	Dojō	DD		NO	YES	YES	YES	YES	YES	YES	YES	Kawanabe et al. (2001), Ozawa et al. (2003), Matsuzawa and Senou (2008), Nakabo (2013)
*Cobitis biwae*	Higashi-shima-dojō			NO	YES	YES	YES	YES	YES	YES	NO	Kawanabe et al. (2001), Ozawa et al. (2003), Matsuzawa and Senou (2008), Nakabo (2013)
*Lefua echigonia*	Hotoke-dojō	EN		NO	YES	NO	NO	NO	NO	NO	NO	Kawanabe et al. (2001), Nakabo (2013)
*Tachysurus tokiensis*	Gibachi	VU		NO	YES	YES	YES	YES	YES	YES	NO	Kawanabe et al. (2001), Ozawa et al. (2003), Kumagai and Katsushima (2012), Nakabo (2013)
*Hypomesus nipponensis*	Wakasagi			NO	NO	NO	NO	NO	NO	NO	NO	Kawanabe et al. (2001), Matsuzawa and Senou (2008), Kumagai and Katsushima (2012), Nakabo (2013)
*Plecoglossus altivelis altivelis*	Ayu			NO	YES	NO	NO	NO	NO	NO	NO	Kawanabe et al. (2001), Matsuzawa and Senou (2008), Kumagai and Katsushima (2012), Nakabo (2013)
*Salvelinus leucomaenis leucomaenis*	Ame-masu, Ezo-iwana			NO	YES	NO	NO	NO	NO	NO	NO	Kawanabe et al. (2001), Matsuzawa and Senou (2008), Nakabo (2013)
*S. leucomaenis pluvinus*	Nikkō-iwana	DD		NO	YES	NO	NO	NO	NO	NO	NO	Kawanabe et al. (2001), Matsuzawa and Senou (2008), Nakabo (2013)
*Oncorhynchus keta*	Sake			NO	YES	YES	YES	YES	NO	NO	NO	Kawanabe et al. (2001), Matsuzawa and Senou (2008), Nakabo (2013)
*O. masou masou*	Sakura-masu, Yamame	NT		NO	YES	NO	YES	YES	NO	NO	NO	Kawanabe et al. (2001), Matsuzawa and Senou (2008), Nakabo (2013)
*Gasterosteus nipponicus*	Nihon-itoyo	LP	LC	NO	NO	NO	NO	NO	NO	NO	NO	Kawanabe et al. (2001), Nakabo (2013)
*Oryzias latipes*	Minami-medaka	VU	VU	YES	NO	NO	NO	NO	YES	NO	YES	Kawanabe et al. (2001), Matsuzawa and Senou (2008), Kumagai and Katsushima (2012), Nakabo (2013)
*Micropterus salmoides*	Ōkuchi-basu	-	-	YES	NO	NO	NO	NO	YES	NO	YES	Kawanabe et al. (2001), Ozawa et al. (2003), Matsuzawa and Senou (2008), Nakabo (2013)
*Cottus kazika*	Kamakiri	VU		NO	NO	NO	NO	NO	NO	NO	NO	Kawanabe et al. (2001), Nakabo (2013)
*C. pollux*	Kajika	NT	NT	NO	YES	NO	NO	NO	NO	NO	NO	Kawanabe et al. (2001), Nakabo (2013)
*C. nozawae*	Hana-kajika	LP	VU	NO	YES	YES	NO	NO	NO	NO	NO	Kawanabe et al. (2001), Nakabo (2013)
*Tridentiger brevispinis*	Numa-chichibu			NO	NO	NO	NO	NO	NO	NO	NO	Kawanabe et al. (2001), Matsuzawa and Senou (2008), Kumagai and Katsushima (2012), Nakabo (2013)
*Rhinogobius nagoyae*	Shima-yoshibobori			NO	YES	NO	NO	NO	NO	NO	NO	Kawanabe et al. (2001), Nakabo (2013)
*R. brunneus*	Tö-yoshinobori			NO	YES	NO	NO	NO	NO	NO	NO	Kawanabe et al. (2001), Ozawa et al. (2003), Matsuzawa and Senou (2008), Kumagai and Katsushima (2012), Akihito et al. (2013)
*Gymnogobius urotaenia*	Ukigori			NO	NO	NO	NO	NO	NO	NO	NO	Kawanabe et al. (2001), Ozawa et al. (2003), Kumagai and Katsushima (2012), Akihito et al. (2013)
*G. opperiens*	Shima-ukigori			NO	NO	NO	NO	NO	NO	NO	NO	Kawanabe et al. (2001), Akihito et al. (2013)
*G. castaneus*	Juzukake-haze	NT		NO	NO	NO	NO	NO	NO	NO	NO	Kawanabe et al. (2001), Akihito et al. (2013)
